# Thermal and optical behavior dataset of surfaces coated with high reflectance and common materials under different conditions, used in Brazil

**DOI:** 10.1016/j.dib.2020.105445

**Published:** 2020-03-19

**Authors:** Alvaro Caldeira e Rosa da Silva, Claudinei Rezende Calado

**Affiliations:** a Materials Engineering Department, Federal Technological Education Centre of Minas Gerais (Cefet/MG, Brazil); b Chemistry Department, Federal Technological Education Centre of Minas Gerais (Cefet/MG), Brazil

**Keywords:** Temperature data, Temperature profiles, Coatings thermal behavior, Surface reflectance index, Surface behavior, Infrared images, Heat flux behavior

## Abstract

External and internal surface temperatures values of coated steel plates and the interior air temperature of a box-like model were collected throughout the time under different conditions: an artificial infrared radiation source was placed facing the external side of the plate to promote the temperature change, the energy power was variated by changing the distance from the plate and simultaneously different airflow conditions were inputted, data was collected by making experiments using the combination of distance and airflow changes. Reflectance raw data, of the materials, was also acquired by measuring the reflectance index of the samples according to ASTM E903 “Standard Test Method for Solar Absorptance, Reflectance, and Transmittance of Materials Using Integrating Spheres”. Amplified optical images and infrared images of the samples were taken (using a “Flir e4” infrared camera) while they were under the influence of the source in the three chosen distances (60 cm, 40 cm and 20 cm). Considering that researches at this particular field lack of more direct comparisons of temperature, reflectance and heat flux behaviours; between special high reflectance coatings and common ones when the substrate is steel, when the direction of airflow is changed and when the source is an infrared one; measurements intending to give faster insights and directions were done. These insights can be used by anyone who intends to develop new coating materials and compare them with already existing ones to streamline their researches in this area. Finally, the buying costs of these coatings, similar to commercially used ones, are showed.

Specifications Table

**Table:** 

Subject	Materials Science - Surfaces, Coatings and Films.
Specific subject area	Temperature, reflectance, optical and infrared imaging of coated surfaces.
Type of data	Table
	Image
	Graph
	Figure
How data were acquired	Temperature data was collected using temperature sensors (type NTC 3950 100 K thermistors), an Arduino microcontroller and software.
	Reflectance index data was collected using a spectrophometer (Perkin Elmer Lambda 1050 spectrophotometer, equipped with a 60 mm diameter integrating sphere) and according to ASTM E903 standard (Standard Test Method for Solar Absorptance, Reflectance, and Transmittance of Materials Using Integrating Spheres.
	Amplified optical images were collected using a digital microscope.
	Infrared images were collected using a Flir e4 infrared camera (all images were taken using the same emissivity setting: emissivity of 0.85 and “shooting distance” of 1 m).
Data format	Raw temperature (°C) - time (s) excel files.
	Raw reflectance – wavelength (nm) text files.
	Raw optical image files.
	Raw infrared image files.
	Organized excel temperature (°C) versus time (s) graphs.
	Organized excel temperature (°C) versus combinations graphs.
	Organized excel reflectance index versus wavelength (nm) files.
	Analysed infrared images files.
Parameters for data collection	- For all temperature measurements: maximum temperatures values were considered when the sensors variations were less than 1 Celsius. The experiments were conducted in laboratory, the sensors fixing method and the energy source distance measurement method were the same for all experiments. The samples coating thickness were measured with a Check line, model 3000FX thickness gage and statistically analyzed to be sure that they had similar values.
	- Samples for reflectance index measurements, were made using the same application method used for making the samples for temperatures measurements. Reflectance index was measured used a calibrated equipment and according to an international standard.
	- All optical images of the samples were taken using a calibrated microscope and at a fixed distance.
	- Infrared images were taken using a calibrated infrared camera at the same fixed distance. For the analysis of the infrared images the temperature scale was the same to favor comparisons.
Description of data collection	Temperature data was collected using temperature sensors (thermistors) and an Arduino Mega 2560 microcontroller connected via USB with a computer by Arduino IDE software.
	The reflectance data was converted directly by the spectrophotometer in text files.
	The optical and infrared images were converted directly by the equipment and transferred to a computer using a USB port.
Data source location	For temperature, optical and infrared images data:
	Institution: Federal Technological Education center of Minas Gerais (CEFET-MG)
	City/Town/Region: Belo Horizonte/Minas Gerais
	Country: Brazil
	Latitude and longitude (and GPS coordinates) for collected samples/data: −19.930297, −43.978516
	For reflectance index data:
	Institution: Federal University of Goiás
	City/Town/Region: Goiania/ Goiás
	Country: Brazil
	Latitude and longitude (and GPS coordinates) for collected samples/data: −16.602564, −49.254584
Data accessibility	Repository name: Mendeley Data Repository
	Data identification number: DOI: 10.17632/gnhjwsf6jf.1
	Direct URL to data: http://dx.doi.org/10.17632/gnhjwsf6jf.2

## 1 Value of the data

• These data provide a way to compare common and high reflectance coatings under infrared energy and airspeed variations, as there are no researches based on the behavior of common and high-reflectance coatings, that are subjected to those simultaneous variations.
• These compiled data provide, at one unique place, information about various properties (normally found in separated works): surface and air temperature values, visual surface aspects, surface radiation flux behavior and reflectivity values of the materials.
• These data provide a base to access the viability of using high reflectance coatings used in Brazil in the year of 2019, based on its temperature reduction potentials.
• These data can benefit researchers that work on the development of new or advanced coatings and materials. It is also suitable for those who need information about the way a material behaves under certain temperatures or conditions, the ones who need to learn fast about these types of material and for personal that seeks practical information.
• These data can be used as a way of comparing existing and development materials, streamlining future works in this field. These can also be used to compare and evaluate results of field tests on materials in use.
• These data facilitate and speed up the development of new researches, existing ones and promote a clear understanding of the modified behavior of coated surfaces.

## 2 Data description

This works contain data obtained from temperatures measurements made by thermistors, diffuse reflectance tests, infrared thermography, optical imaging and commercial prices.

The “raw temperatures data .xlsx” files, which are the imported values measured by the thermistors, obtained via the Arduino IDE software. E.g.: “Raw temperature data 20cm_no airflow_1″ file are the data collected by the first experiment; when the radiation source was placed at 20 cm distance from the plates and no airflow was acting on the surfaces. This raw temperature data was filtered and organized; and the profile temperatures curves (Fig. 2, Fig. 3, Fig. 4, Fig. 5, Fig. 6, Fig. 7, Fig. 8, Fig. 9, Fig. 10), were made according to the sensor positions. This temperature profile curves and its tabulated data, are in another tab of the same raw file.

This raw temperature data was also organized into the propose combinations creating the graphs showed in Fig. 11.

The first graph in Fig. 11 has the temperatures for the grouped samples: e.g. the first three black colored bars are respectively the maximum temperatures for TC-01 sample for experiments made with no airflow; with lateral airflow and with frontal airflow.

The device that was built to measure the above-mentioned data can be seen at supplemental data 1.

“Raw diffuse reflectance data .csv” files, which are the data obtained directly by the spectrophotometer. This data was exported to excel software and organized by the amount of energy reflected of each wavelength, creating “Diffuse reflectance versus wavelength graphs” file and the graph showed in Fig. 12. To make things clearer; the equipment emits one type of wavelength (ultraviolet, visible and infrared) at a defined interval of time and the amount of energy reflected by the surfaces is measured using the integrating sphere. E.g.: “Raw diffuse reflectance_TC-01″ file has the reflectance test raw data for sample TC-01.

**Fig. 1:**
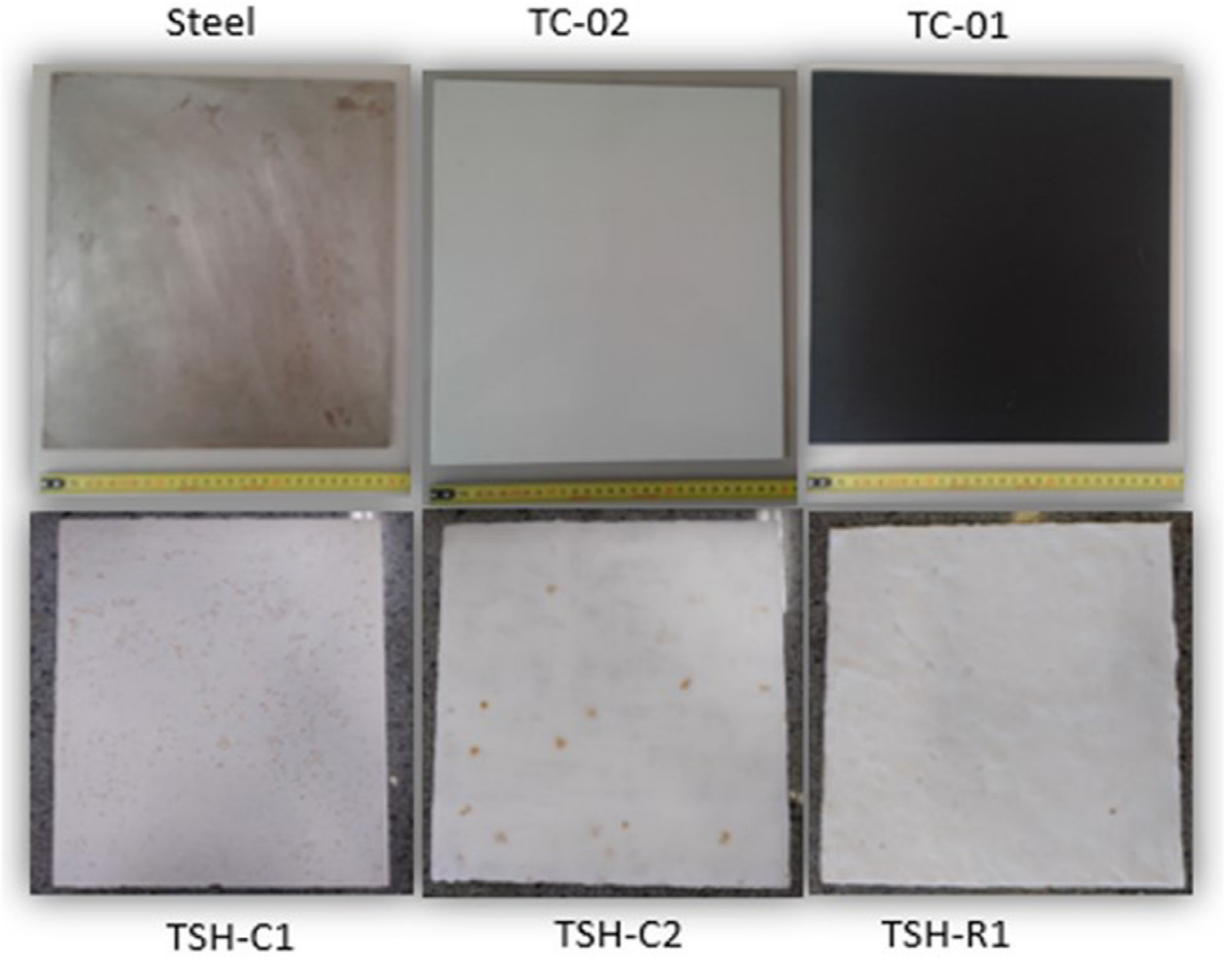
- Description and pictures of the analyzed materials.

“Optical images .jpg” files have the twenty times amplified images of the surfaces of the samples. Imagens were taken from different points of the plates to show all the aspects observed. One image of each sample is shown in Fig. 13. Table 1 has a basic description and Fig. 1 has the pictures of the samples used to measure the temperatures and to collect the thermographic images, these were taken using a normal camera.

**Table 1:** Description of the samples.

N°	Sample	Color	Average coating Thickness (µm)	Sample description
1	TC-01	Black	591.52	Common acrylic-based paint
2	TC-02	White	649.32	Common acrylic-based paint
3	TSH-C1	White	846.93	High reflectance composite paint
4	TSH-C2	White	812.17	High reflectance composite paint
5	TSH-R1	White	596.98	High reflectance composite paint

The “Raw infrared thermographic images .jpg” files are the thermographic images taken by the thermographic camera (supplemental data 6) of each material, considering only the source distance changes (no airflow was used in this test). The emissivity was changed, ranging from 0.75 to 0.90 for each distance. Then, these images were filtered and Figs. 15 to 17 were obtained using the own camera software (Flir tools).

These images were filtered to consider only a central region of the plates (to avoid errors generated by the edges and external influences). A fixed temperature scale was used to favor the comparisons. Table 2 has the maximum temperatures for each distance from the radiation source, obtained when the thermographic camera emissivity was changed.

**Table 2:** - Maximum external temperatures for the combinations: 60 cm/no airflow; 40 cm/no airflow and 20 cm/no airflow; measured using the infrared camera.

The datasheet of the temperature sensors (thermistors) used to collect the temperature data and the datasheet of the microcontroller used for their electronic control can be seen respectively in “Supplemental data 2″ and “supplemental data 3″ if necessary.

To be easy to analyze this huge amount of data, tables were made, and can be consulted in “Supplemental data 4″, with the maximum average temperatures in Celsius and Kelvin for all combinations of “distance/airflow”.

A photo of the portable microscope and its technical specifications, used to generate the amplified optical images are available in “Supplemental data 5″.

“Supplemental data 6″ has the infrared thermographic camera manual, for technical specifications consulting if needed.

“Supplemental data 7″ has a table that contains the maximum temperature values for the three distances and for the changes on the emissivity of the infrared camera.

The yellow marked values are the ones closest to the temperatures measured by the sensors. Using this table, insights about the materials emissivity can be made, but having in mind that they are approximations and this property can only be well measured with the use of more advanced techniques that are not the scope of this work.

This file also has graphs, in Celsius and Kelvin, of the maximum temperatures when the radiation source is at the distance of 20 cm from the plates.

“Supplemental data 8″ contains the tabulated data for Fig. 11 graphs. These data have the maximum temperatures for each sample and each distance from the source/airflow combination.

Table 3 has the price of the samples used in this work, considering the covering efficiency and an area of 1 m^2^. This price was converted using the price of R$1,00 (Brazilian real) equal to U$4.14 (US dollar) .

**Table 3:** - Commercial costs of the analyzed samples.

Sample	Commercial price (US$/L)	Coverage efficiency (L/m^2^)	Area (m^2^)	Cost (US$)
TC-01	165.6	0.6	1	99.36
TC-02	258.75	0.6	1	155.25
TSH-C1	322.09	0.6	1	193.26
TSH-C2	405.72	0.6	1	243.43
TSH-R1	285.25	0.6	1	171.15

*Area used as reference: 1m^2^. Considering US$1.00 = *R*$4.14 (August 2019).

## 3 Experimental design, materials, and methods

Five samples and their descriptions (Fig. 1 and Table 1) formulated and supplied by an industrial collaborator were analyzed: two common commercial acrylic based paints and three with high reflectance pigments in its compositions.

These samples were applied (on the external side only) on ABNT 1020 steel plates, with dimensions of 0.3 m high, 0.3 length and 1.5 m thick. After drying completely, they were sanded, leaving the surface as smooth as possible and keeping the thickness of the samples as close to each other, to eliminate this variable influence on the results.

Common paints were applied using the air spray method, while high reflectance ones were applied using a brush, this was made on purpose to simulate different and common in-field methods.

To be able to collect temperature data from each type of material, a device (supplemental data 1) was built. This device consisted in a box-like structure made of plywood and externally isolated with Ethylene Vinyl Acetate (EVA). One of its side was removed to place the coated steel plates. In another similar structure, an uncoated ABNT 1020 steel plate was placed and the temperature values of this plate were measured in the same points and at the same time to be used as a reference.

To collect data, thermistors (supplemental data 2), a Arduino Mega 2560 microcontroller (supplemental data 3) and the free version Arduino IDE software were used.

An artificial infrared radiation source promoted the temperature increasing. By varying the distance of this source from the plates (60, 40 and 20 cm), the amount of energy that hits these materials could be changed.

Also, the speed and directions of the air were modified. Three airflow configurations were chosen for the tests: no airflow acting, airflow with speed of 1 m/s hitting frontally the external surface of the plates and airflow with speed of 1 m/s hitting laterally the external surface of the plates. Using these configurations, we could see the influence of the convective heat coefficient in the temperatures.

Temperature measurements are a well-known technique used by many researchers: Hongbo, L., et al. (2014) [1], used sensors to measure temperature values of coatings under solar radiation, arranging the results in the form of graphs. Revel, G.M., et al. (2014) [2] used sensors to evaluate building envelope materials and Synnefa, A., Santamaouris, M., Livada, I. (2006) [3], studied reflective coatings on buildings for urban environment uses.

The experiments were conducted based on these “distance/airflow” combinations.

Each test had durations between one and a half hour and two hours, depending on the sample response to the energy input and its temperature varition during “heating” period. The procedure for each one of them was the following:

1 - The experiment began at room temperature.
2 - The radiation source (and the fan, when suitable) was turned on and temperature measurements were collected, until temperature variation was less than 1 °C (to promote temperature equalization).
3 - The radiation source was then turned off and data were collected until the period was completed.

**Fig. 2:**
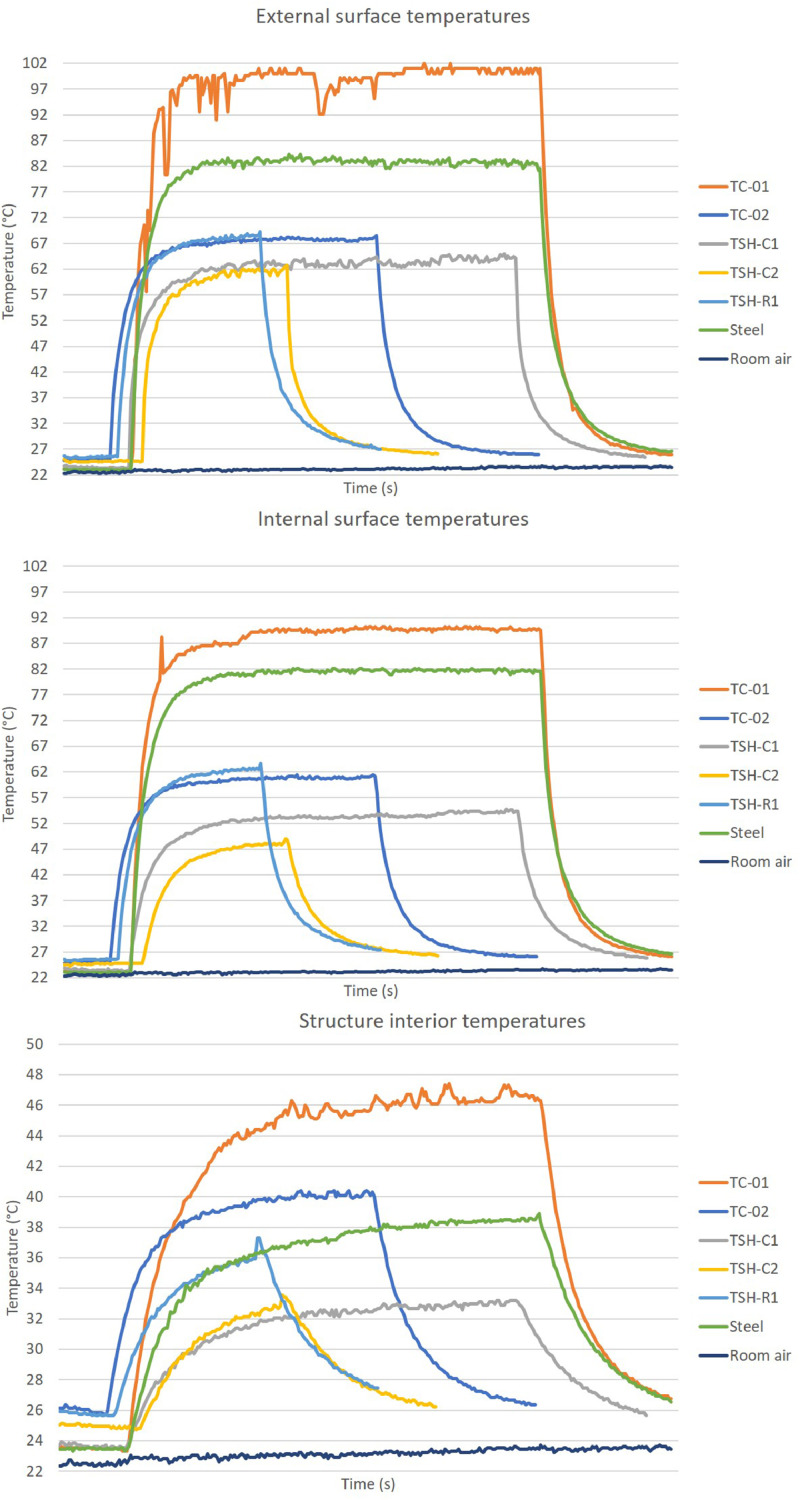
Temperature profiles of the coated samples. The graphs appear in the following order: 20 cm/no airflow.

**Fig. 3:**
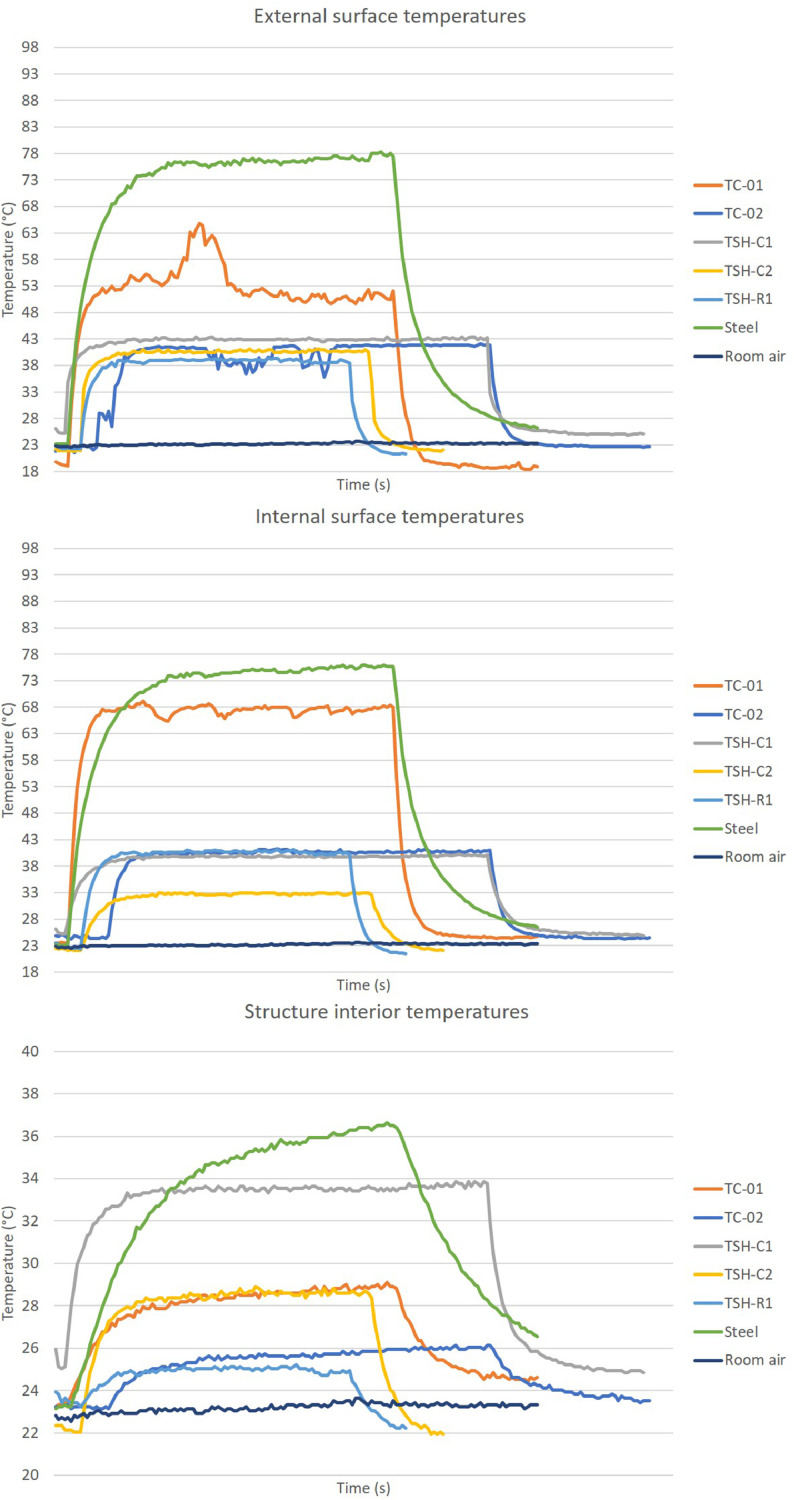
20 cm/frontal airflow.

**Fig. 4:**
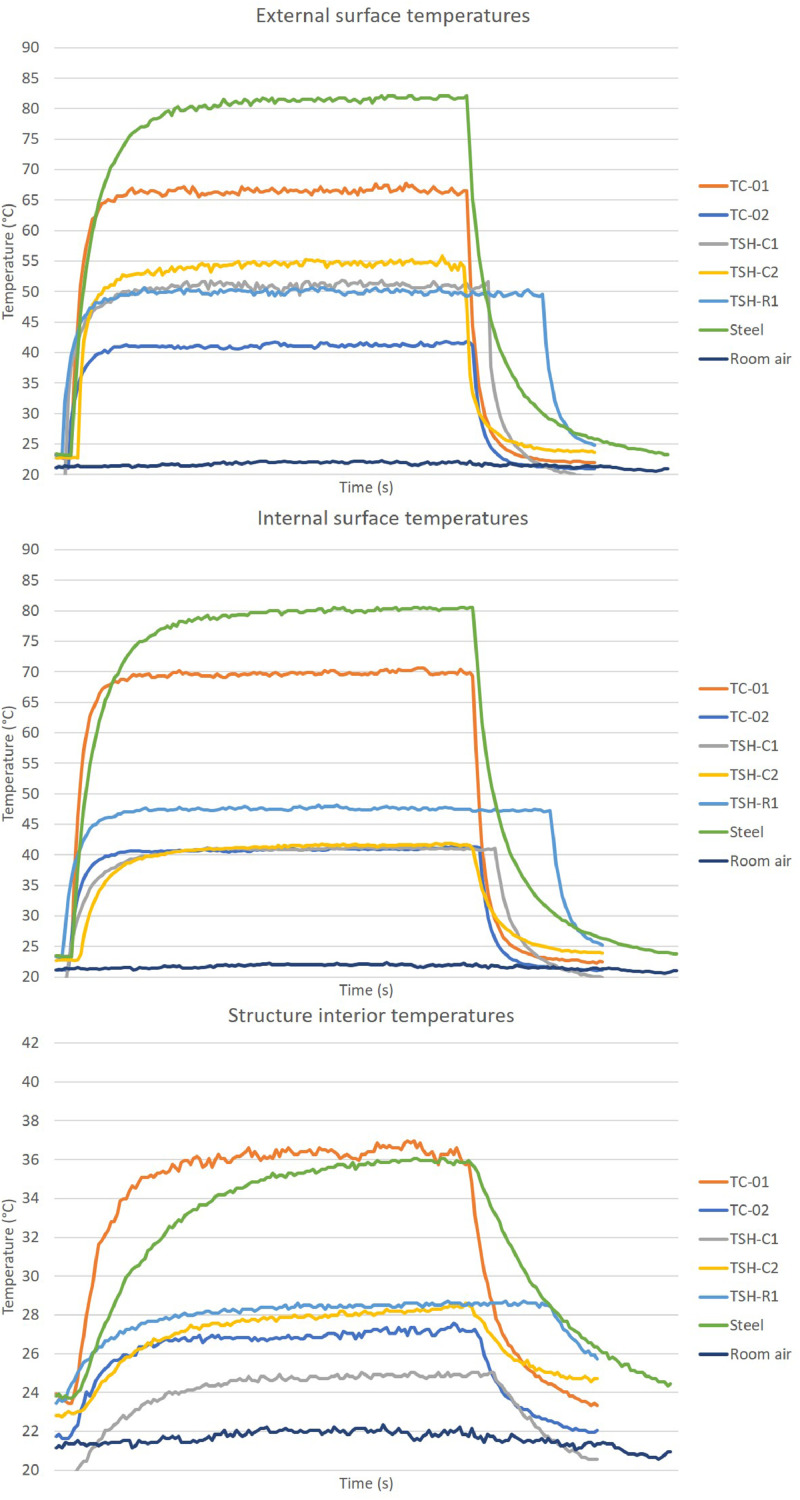
20 cm/lateral airflow.

**Fig. 5:**
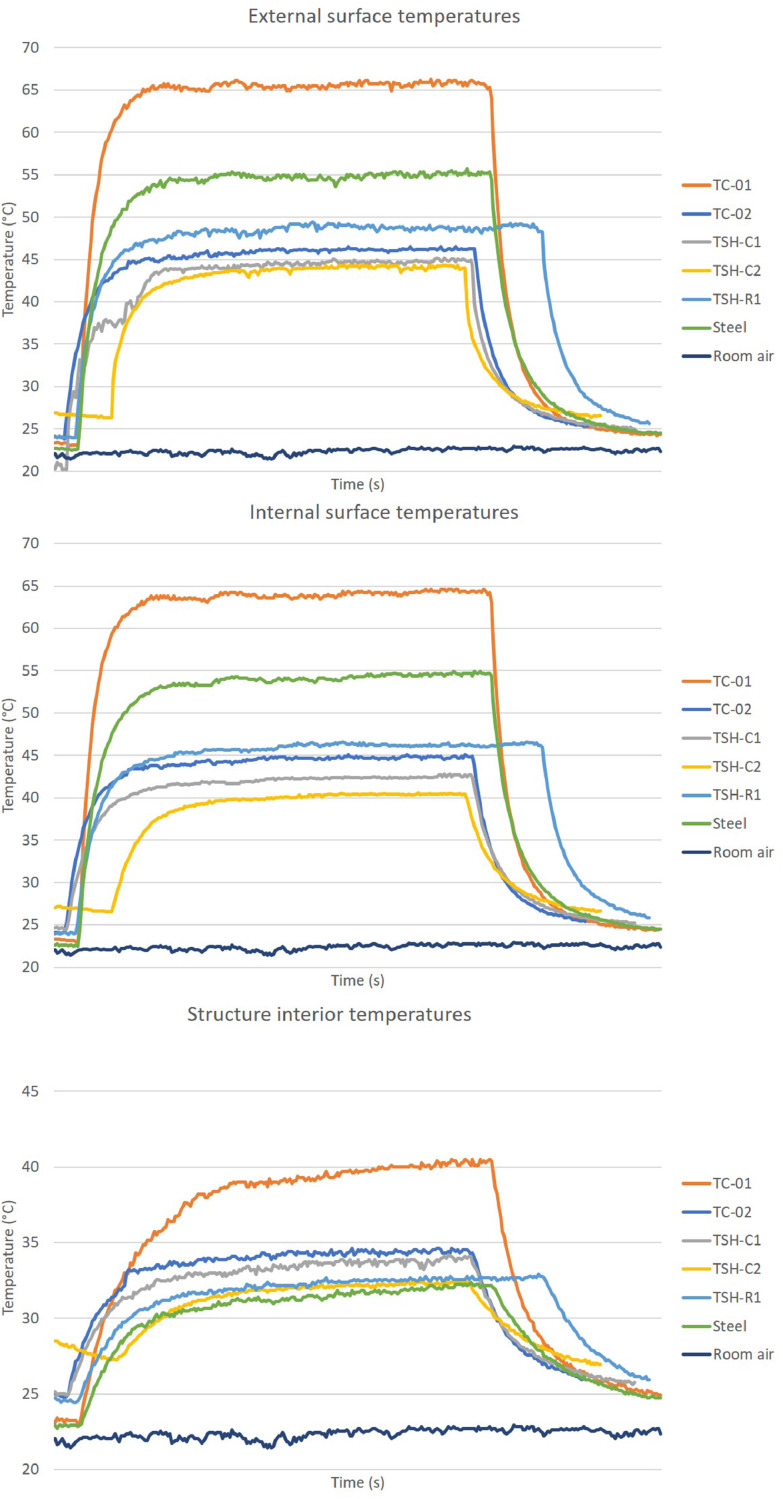
40 cm/no airflow.

**Fig. 6:**
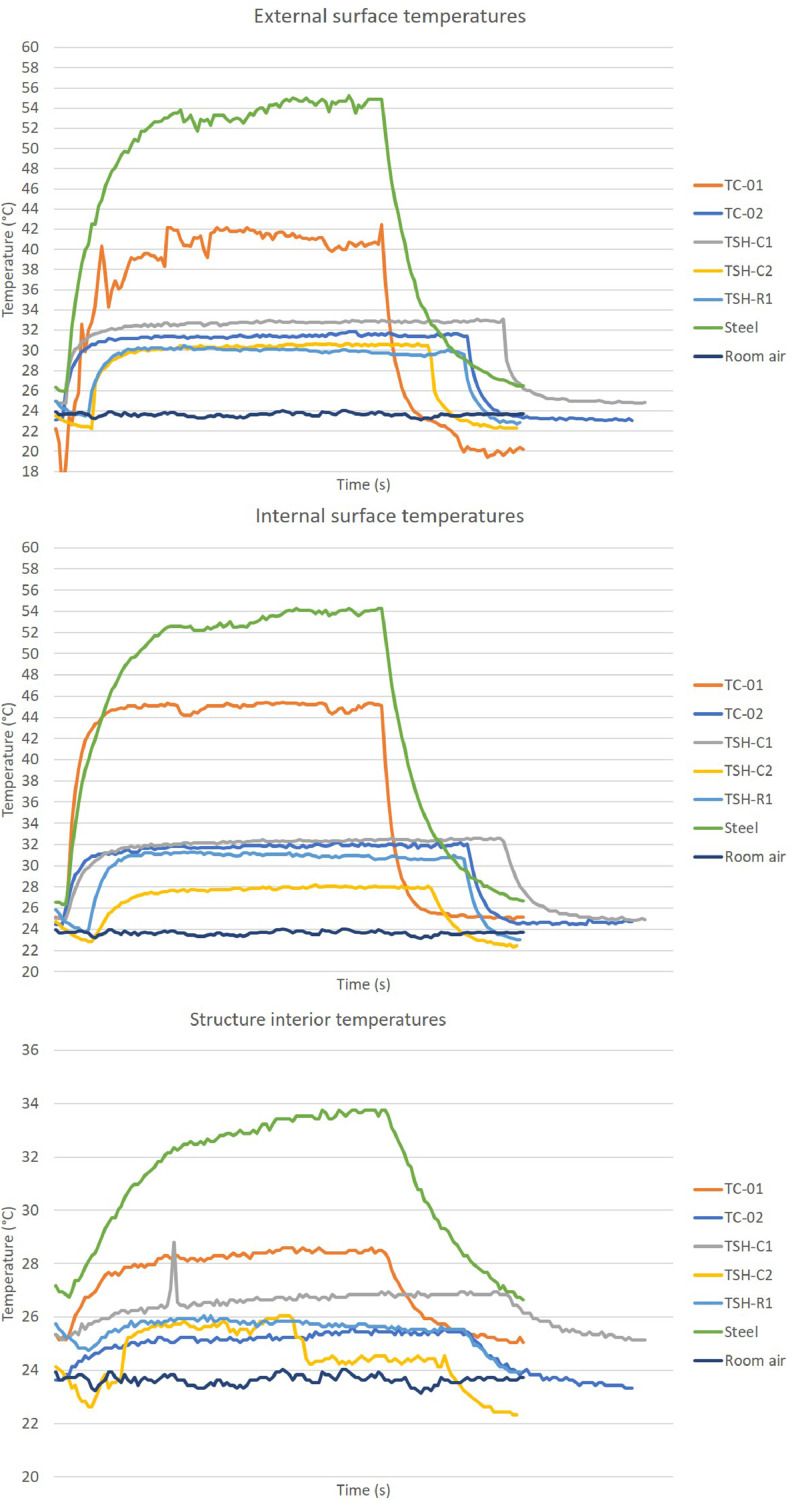
40 cm/frontal airflow.

**Fig. 7:**
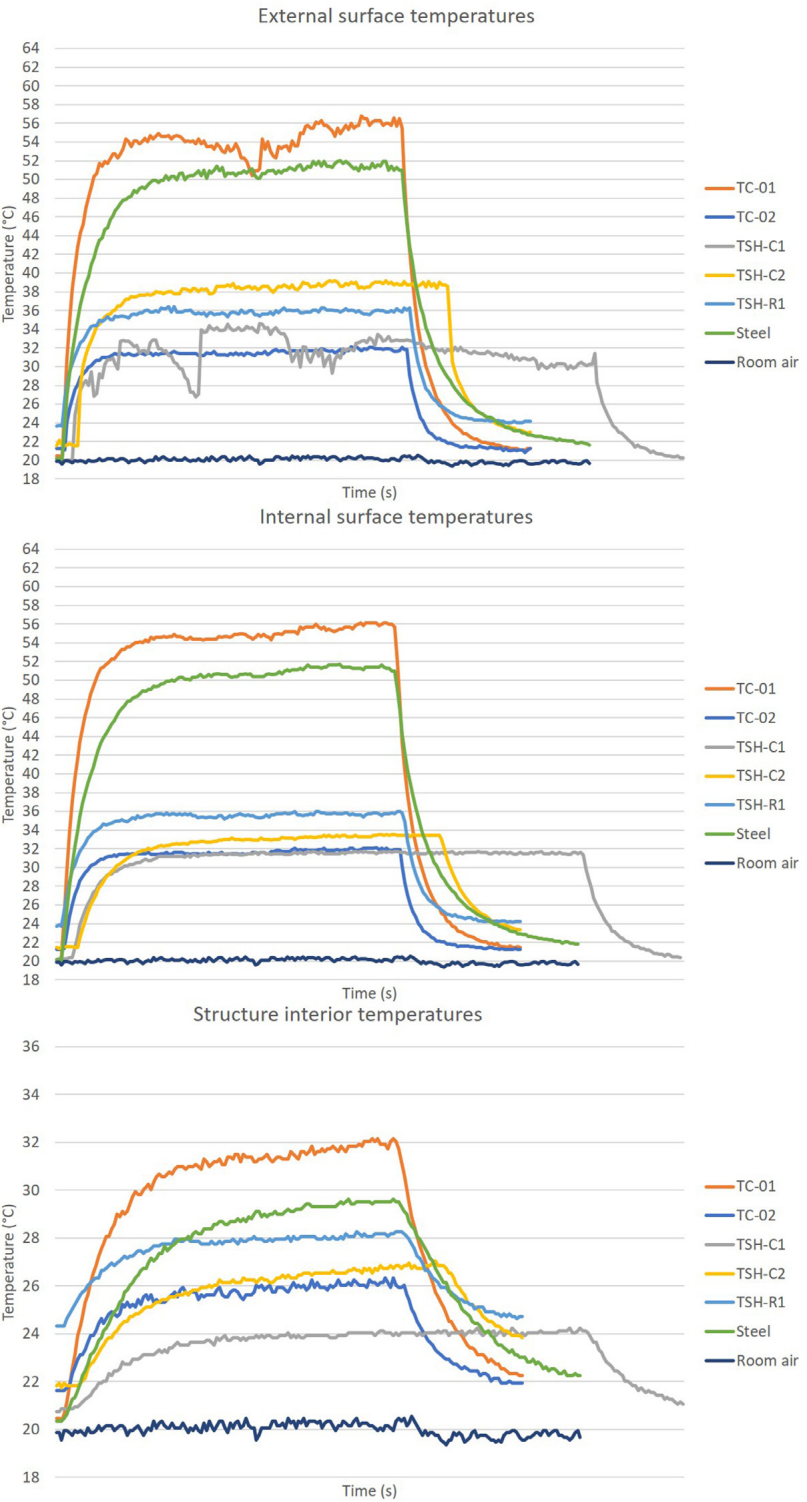
40 cm/lateral airflow.

**Fig. 8:**
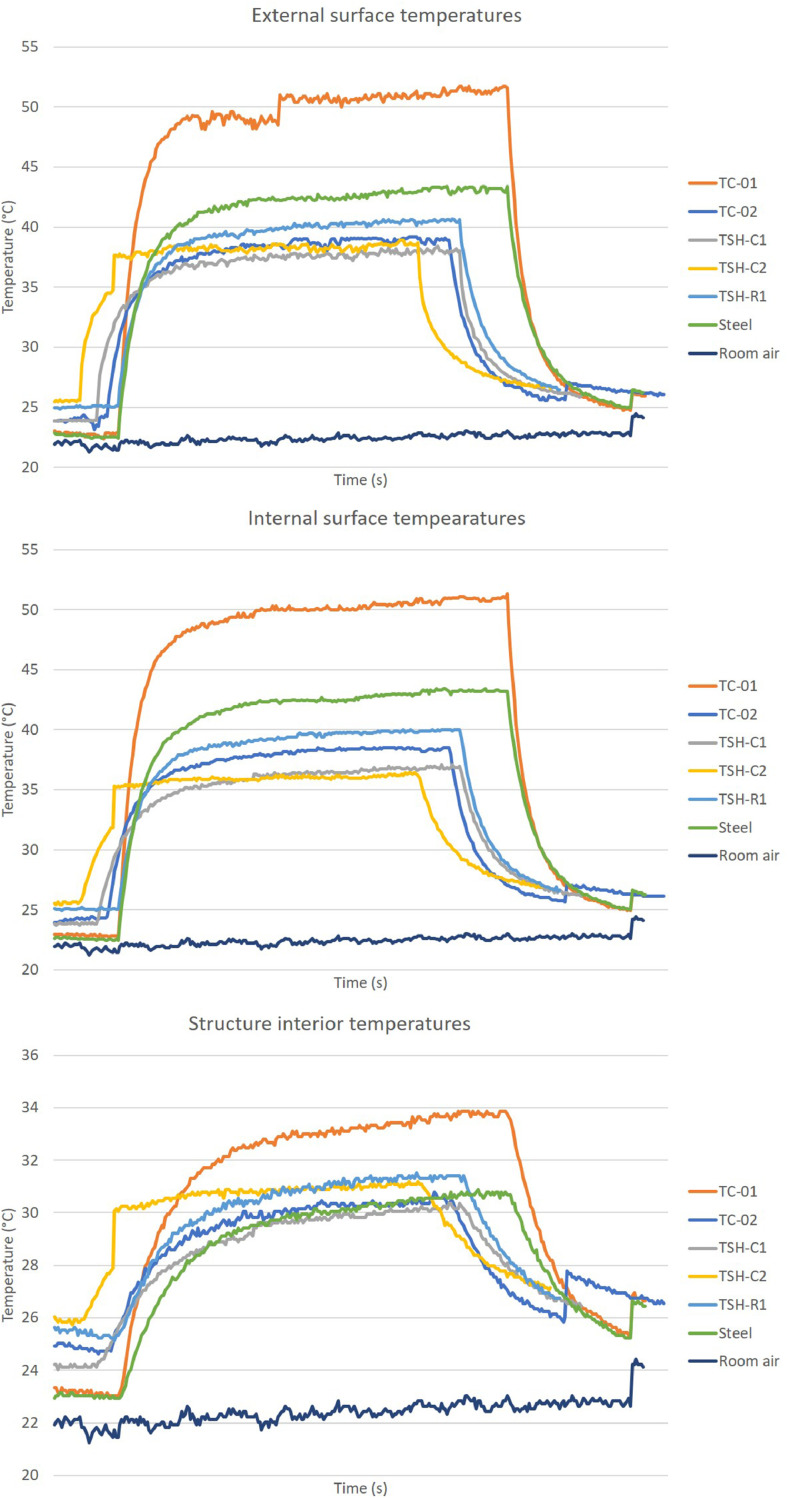
60 cm/no airflow.

**Fig. 9:**
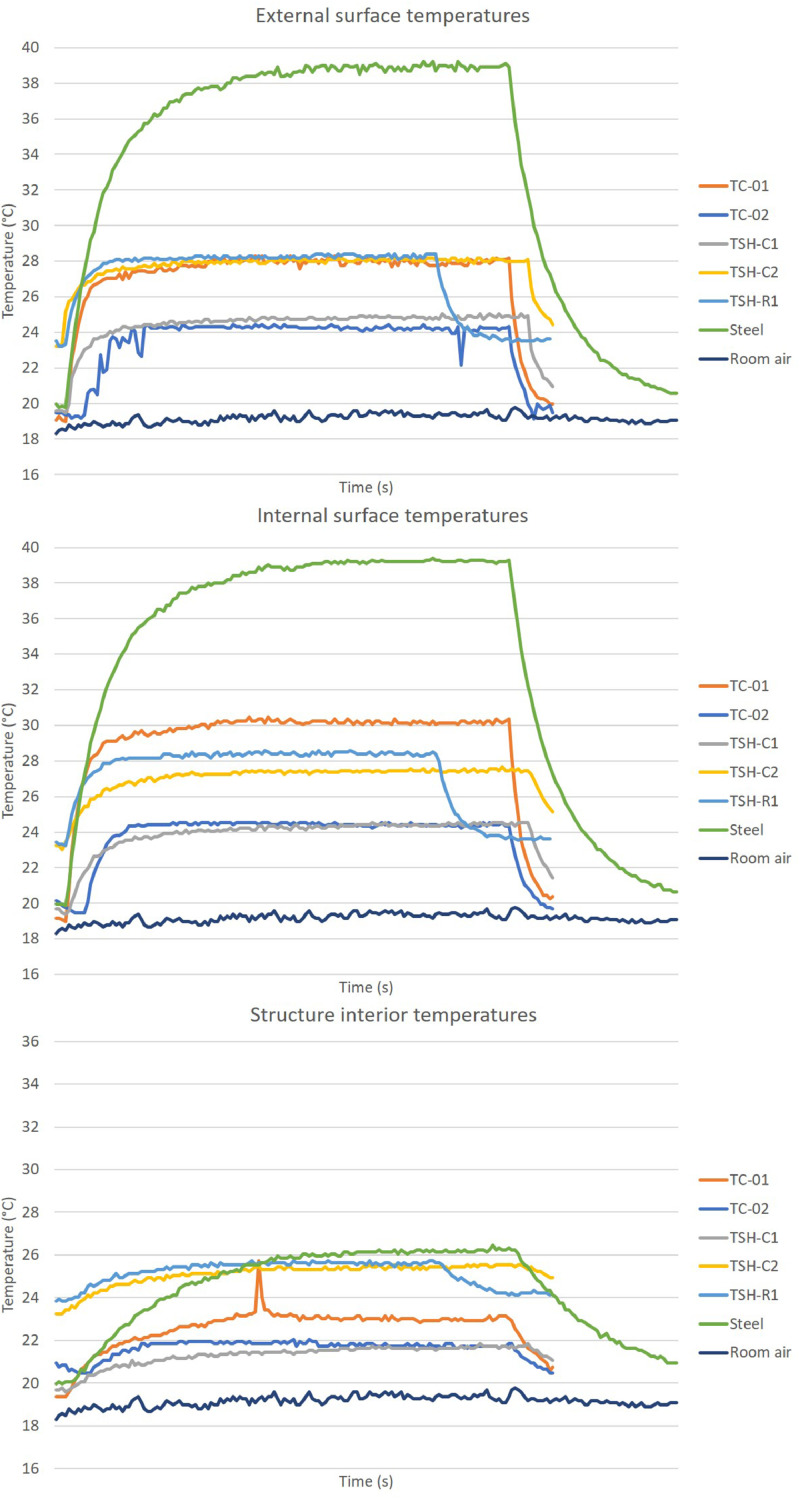
60 cm/frontal airflow.

**Fig. 10:**
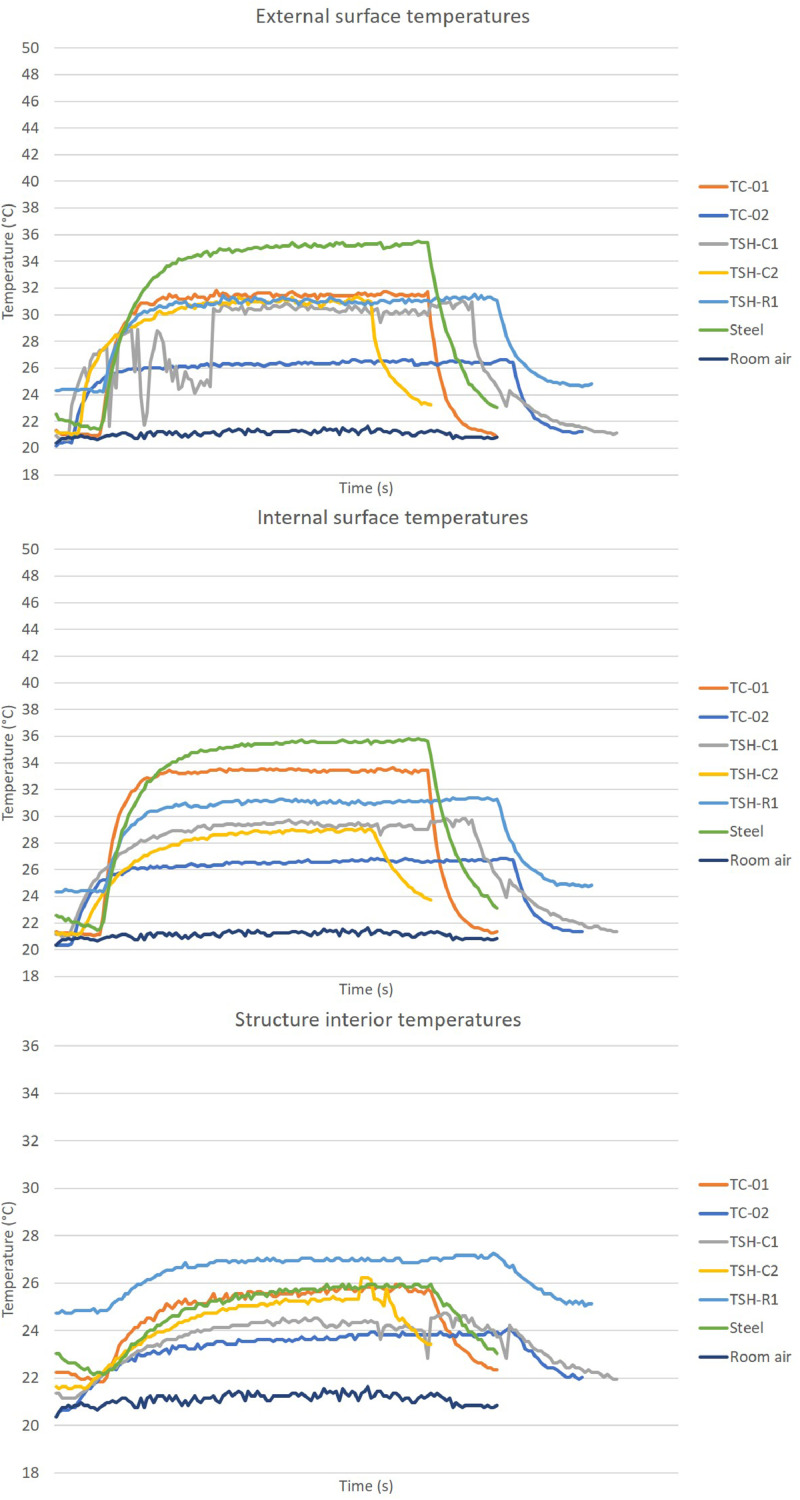
60 cm/lateral airflow.

**Fig. 11:**
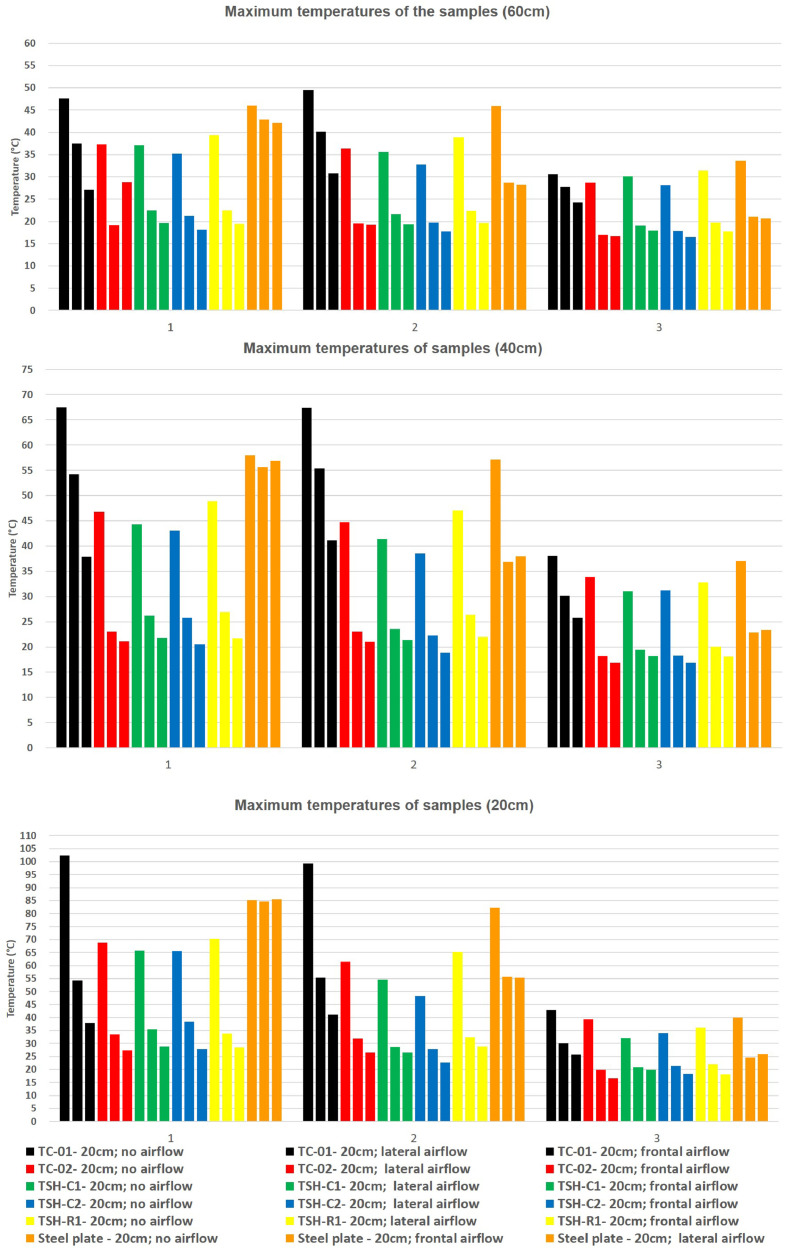
- Temperature versus distance/airflow variations. * 1- External surface temperatures; 2- Internal surface temperatures; 3- Structure interior temperatures.

Data were organized in two different ways: as temperature profile graphs (temperature versus time), (Fig. 2, Fig. 3, Fig. 4, Fig. 5, Fig. 6, Fig. 7, Fig. 8, Fig. 9, Fig. 10) according to the sensor's locations, distances and airflows; to show how temperature behaves when the material is heated and cooled. And, as temperature versus combinations graphs (Fig. 11) to see the influence of air speed and directions changes. If the temperature values were shown in Kelvin, the temperature variations would be hard to see so, the graphs were made in Celsius degree to make these variations easier to see. The maximum average temperatures regarding all combinations are in the tables of supplemental data 4 file.

The figures show the temperatures profile only for one of the two or three experiments done for each combination.

Room air curves, in each graph, refers to the ambient air and are the temperature values of the reference plate of only one of the tests performed, since observing the collected data it can be seen that the ambient temperature did not vary significantly for the same set of samples tested.

Reflectance tests are made mainly to evaluate the isolation potential of materials used to build a structure or building, the more a material reflects a type of radiation the lower is its temperature on the other side. Experiments done by Rossi, F., et al. (2016) [4] with retro-reflective materials and by Coser, E., Moritz, V.F., Krenzinger, A., Ferreira, C.A. (2015) [5] with paints with infrared reflective properties, shows the use of this technique. Some other authors: Uemoto, K.L., Sato, N.M.N., John, V.M. (2010) [6] and Synnefa, A., Santamouris, M., Akbari, H. (2007) [7], have used temperatures measurements along with reflectance test to make assessments about its efficiency.

**Fig. 12:**
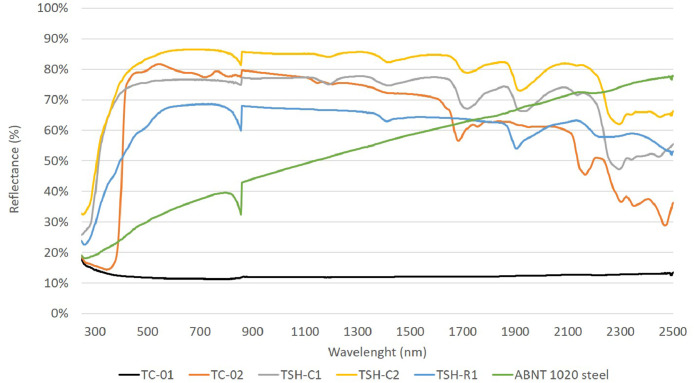
- Diffuse reflectance of all the samples for wavelengths from 250 nm to 2500 nm.

The diffuse reflectance data was collect using a Perkin Elmer Lambda 1050 spectrophotometer, equipped with a 60 mm diameter integrating sphere and according to ASTM E903 standard (Standard Test Method for Solar Absorptance, Reflectance, and Transmittance of Materials Using Integrating Spheres). These raw data were organized, and a graph of reflectance versus wavelength of the samples was created (Fig. 12).

**Fig. 13:**
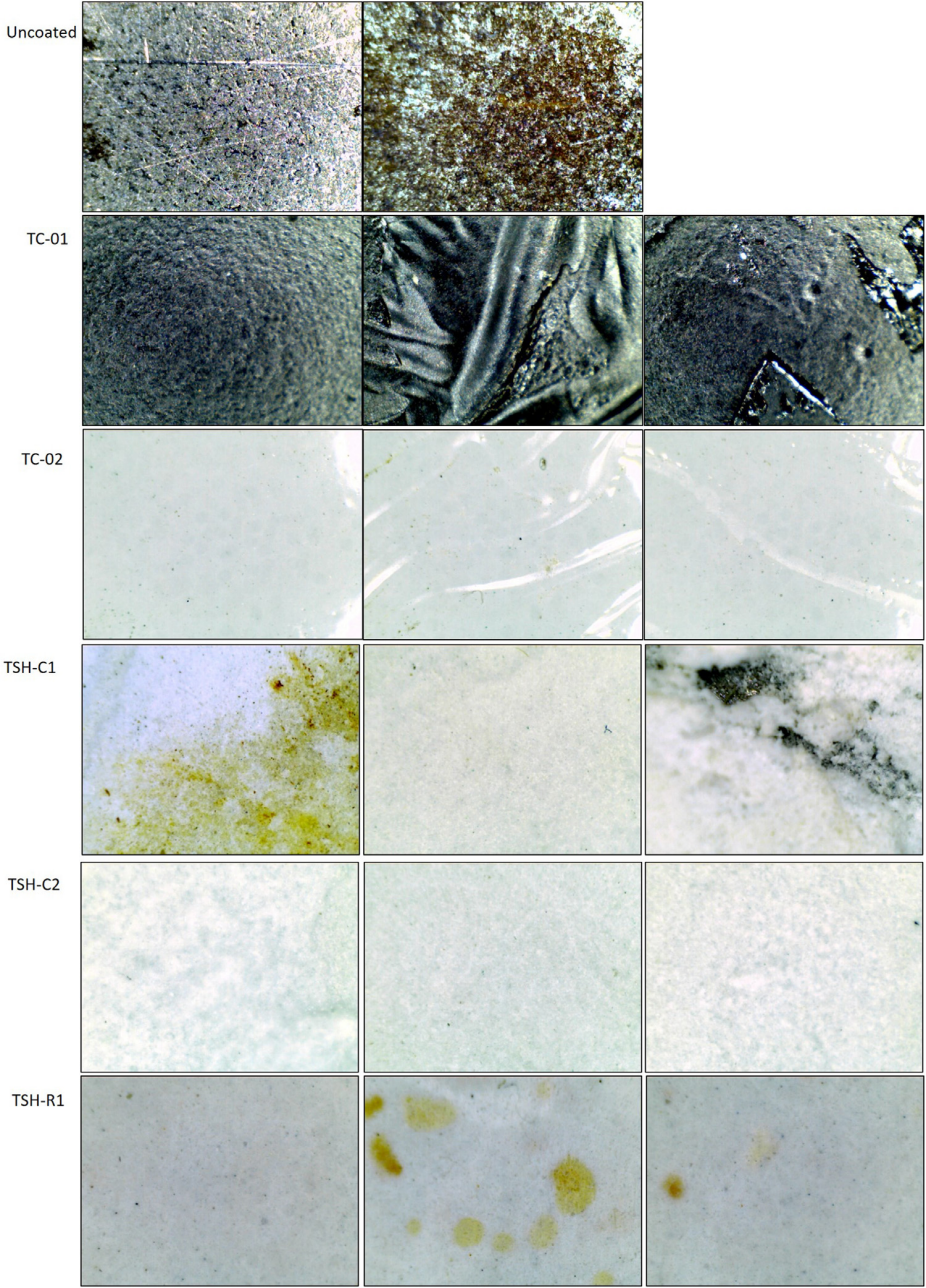
- Amplified optical imagens of the coated surfaces.

The amplified (twenty times) optical images (Fig. 13) of the coated and uncoated external surfaces, were collected using a portable digital microscope (with technical specifications describe at “supplemental data 5″) at a fixed distance from the surfaces. Those images were taken at different points of the plate to show all their aspects.

Infrared thermographic tests are used to evaluate the thermal behavior of a surface, its heat flux, to collect surface temperature values and to see possible defects. Calado, C.R., et al. [8] used it to detect manufacturing internal defects in ceramic tiles.

The thermographic images were collected using a Flir e4 infrared camera (Supplemental data 6) and all images were taken using the emissivity of 0.85 and at “shooting distance” of 1 m.

**Fig. 14:**
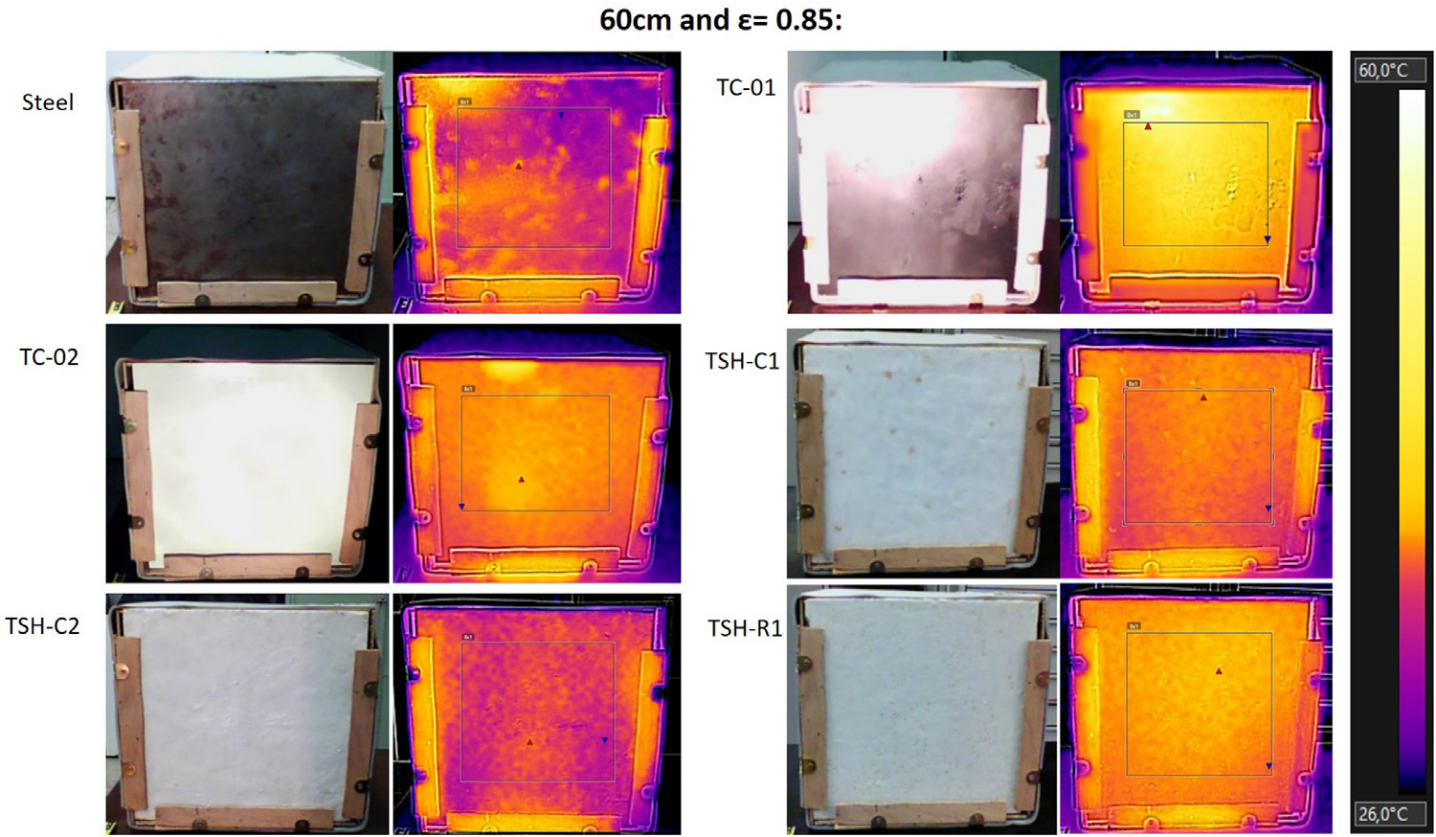
Filtered infrared images of the external surfaces for the combinations of 60 cm/no airflow;.

**Fig. 15:**
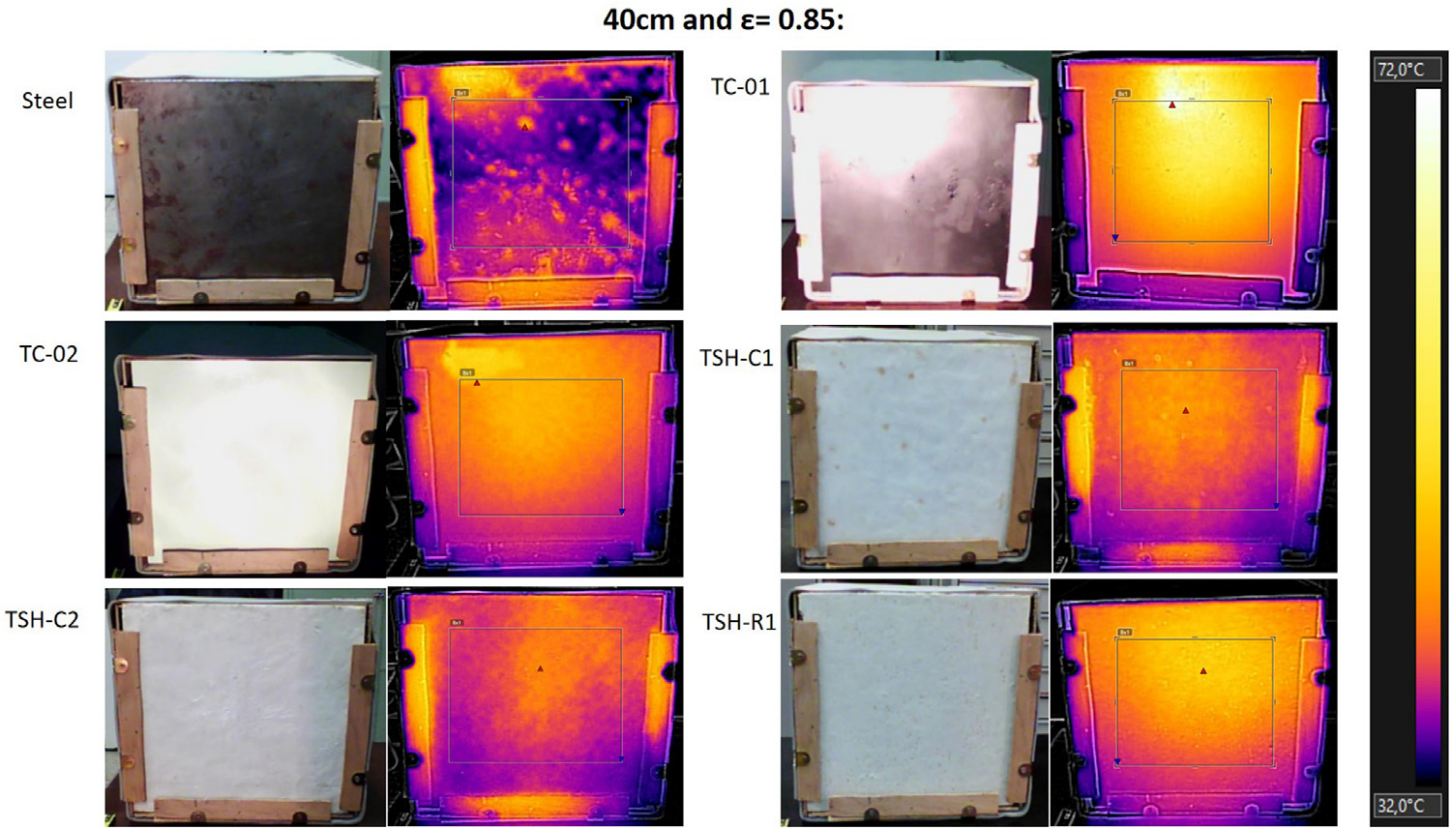
40 cm/no airflow.

**Fig. 16:**
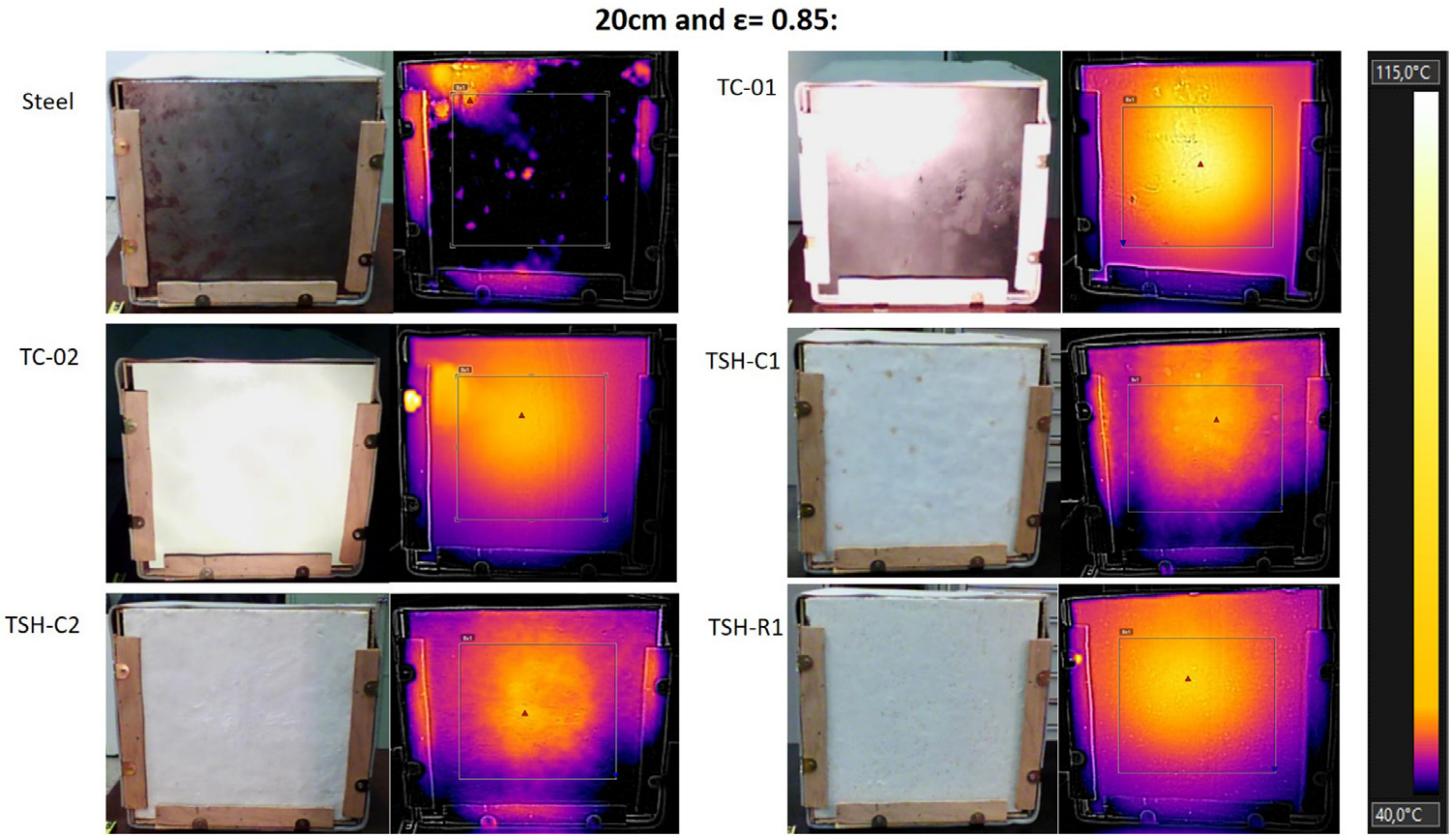
20 cm/no airflow.

Filtered thermographic images (Figs. 14, Fig. 15, Fig. 16) consist of the infrared images that shows the temperatures values and also the heat flux behavior at the surface. The central area of the plates was highlighted to eliminate the possible influence of the edges and a fixed scale for each distance was used to favor the comparisons. No airflow was acting when these measurements were made.

All thermographic images were filtered using the same temperature scale to favor comparisons. Table 2 shows the maximum external temperatures found in Celsius, for the three distances and for changes on the emissivity of the infrared camera. Supplemental data 7 shows the maximum temperatures in the absolute scale (Kelvin), when the camera emissivity was changed and the graph of the maximum temperatures of each sample when the radiation source is at 20 cm from the plate surface.

Finally, the average cost of the common and high reflectance coatings used at this work, similar to the ones found in the market of Brazil, is showed in Table 3. Most of the time, researchers do not have access to the composition of the material used due to patent issues. So, the use of experimental thermal behavior along with the coating cost or price can be an alternative to access the viability of a particular material.

## Appendix Supplementary materials

Image, image 1

Image, application 2

Image, application 3

Image, application 4

Image, application 5

Image, application 6

Image, application 7

Image, application 8

Image, application 9

Image, application 10
