# Supplementary figures and images for: Thermal and optical behavior dataset of surfaces coated with high reflectance and common materials under different conditions, used in Brazil
Source: Data Brief. 2020 Mar 19;30:105445. doi: 10.1016/j.dib.2020.105445 (PMC7132077; doi:10.1016/j.dib.2020.105445)

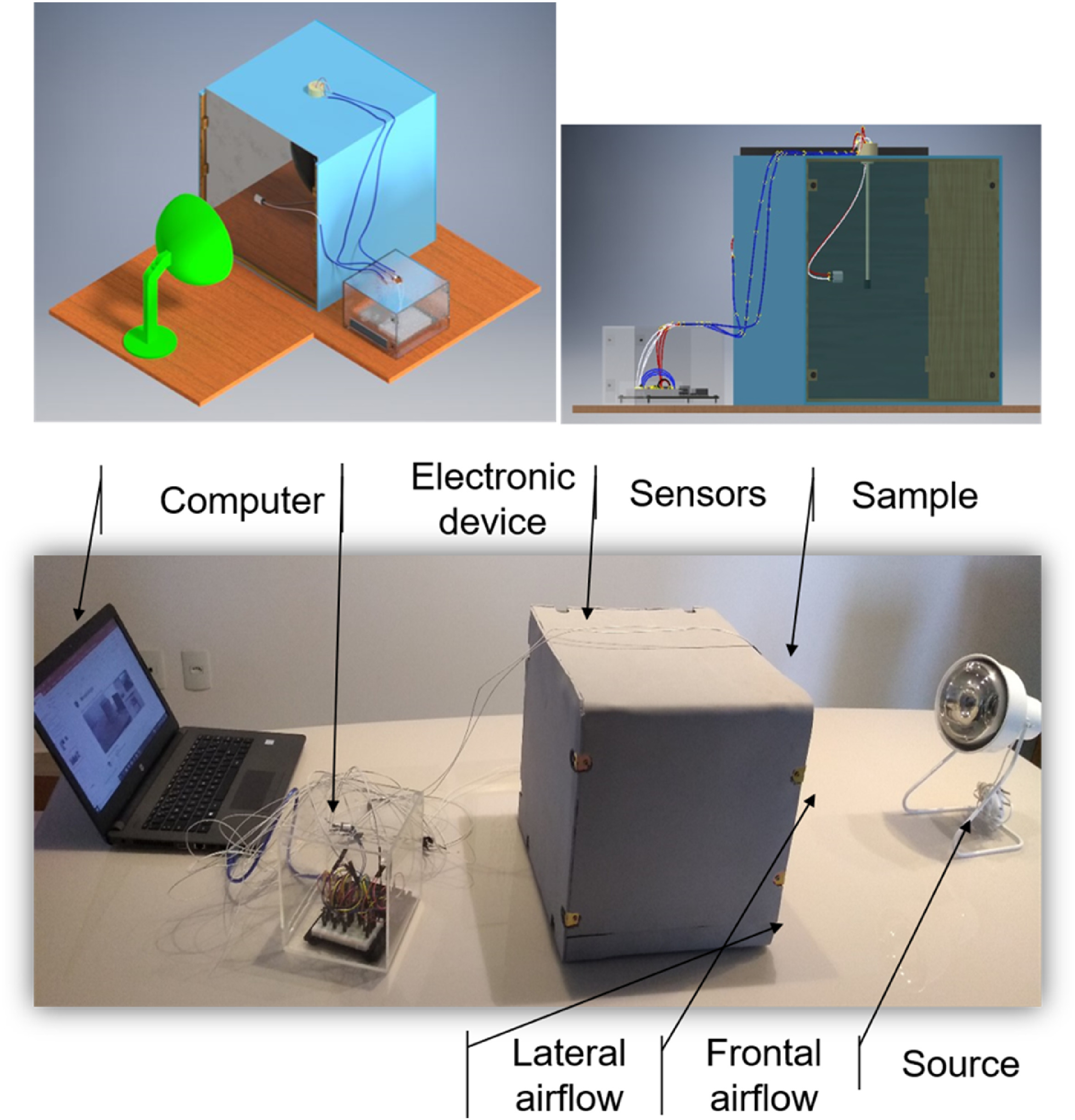

Supplement: Supplementary file 1 [file mmc1.jpg]

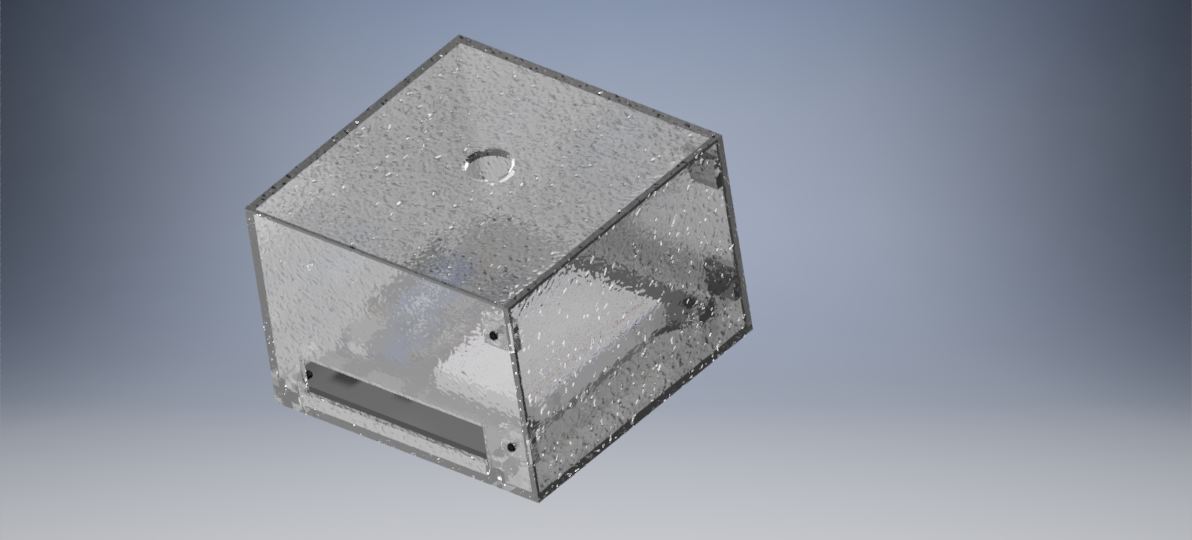

Supplement: Supplementary file 10 [file mmc10.zip › Acrylic box for electronic device protection.jpg]

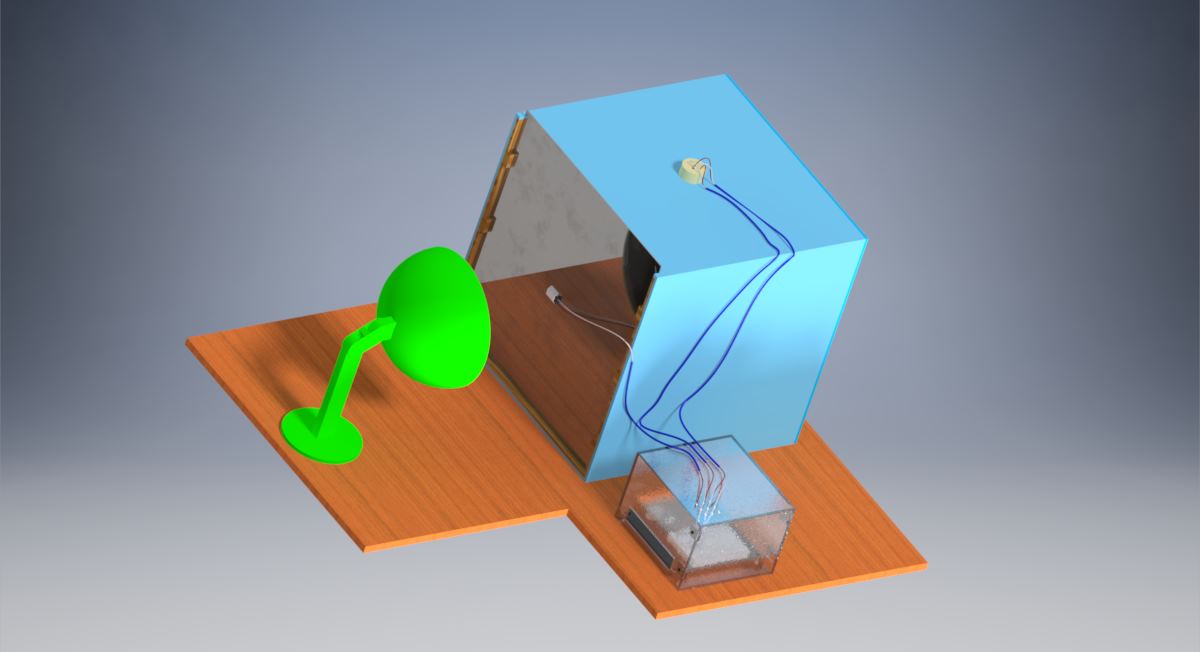

Supplement: Supplementary file 10 [file mmc10.zip › Complete prototype_3D.jpg]

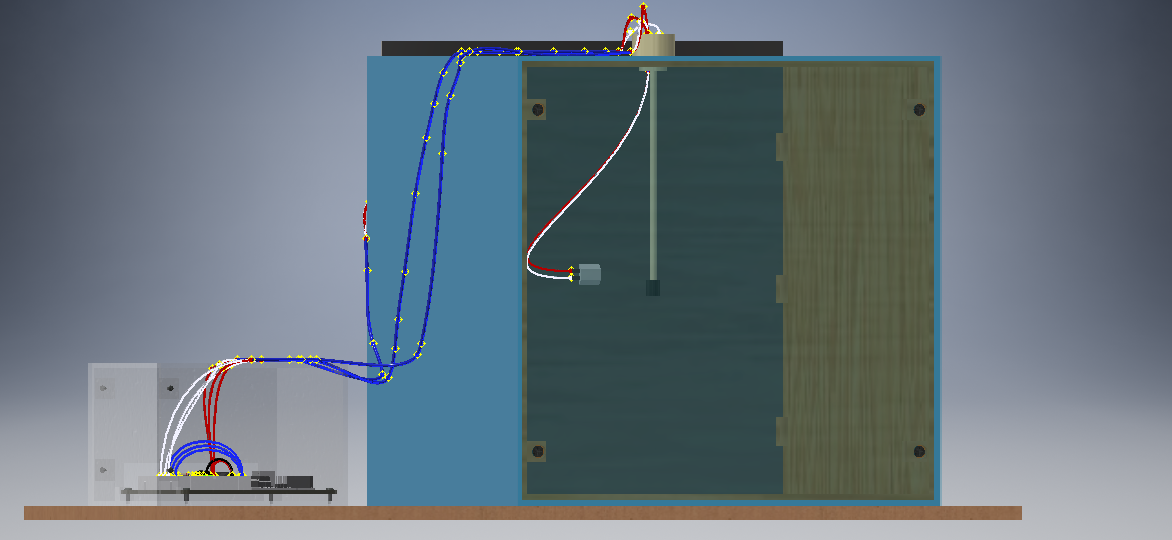

Supplement: Supplementary file 10 [file mmc10.zip › Internal 3D image of the prototype.bmp]
