# Supplementary material for: Thermal and optical behavior dataset of surfaces coated with high reflectance and common materials under different conditions, used in Brazil
Source: Data Brief. 2020 Mar 19;30:105445. doi: 10.1016/j.dib.2020.105445 (PMC7132077; doi:10.1016/j.dib.2020.105445)
Supplement: Supplementary file 2 [file mmc2.pdf]

Reprap Hotend Thermistor NTC 3950 100K with 1M Cable

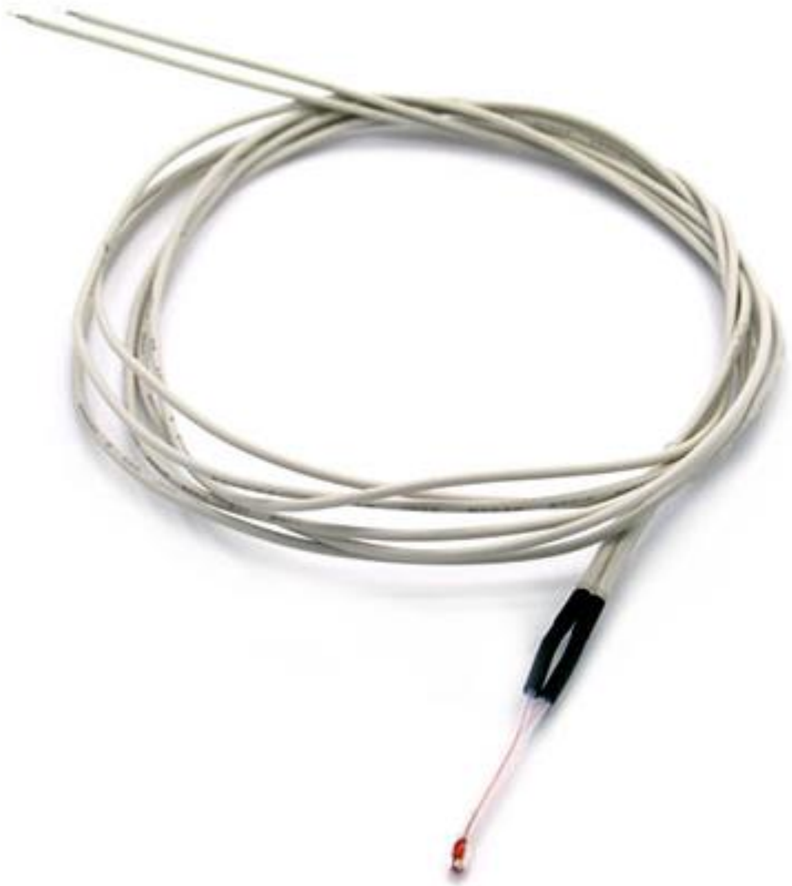

- Standard resistance: R25(25): 1.5K, 2K, 5K, 10K, 20K, 30K, 47K, 50K, 100K, 200K, 500K, etc

- Resistance tolerance: ±1%, ±2%, ±3%

- B-value R25/50: 3435K, 3600K, 3950K, 3990K, 4100K, 4200K, etc

- B-value accuracy: ±1%

- Operating temperature: -40~+300'C

- Power dissipation coefficient: >= 5 mW/'C (in static air)

- Max. rated power: 45mW

- Thermal time constant: <=7S (in static air)

- Temperature coefficient of resistance: -2~-5%/'C

- It is recommended to use: R25'C = 100K, B25/50 = 3950K±1%

Buy link: http://www.makeralot.com/reprap-hotend-thermistor-ntc-3950-100k-with-1m-cable-p176/

Reprap Hotend Thermistor NTC 3950 100K with 1M Cable

R---T TABLE

R25℃=100KΩ±1%　　　B25℃/50℃=3950±1%

T	Rmax	Rnor	Rmin	T	Rmax	Rnor	Rmin
-30	1805.1971	1733.2000	1663.6657	10	203.3838	199.9900	196.6338
-29	1693.5863	1630.4080	1569.3019	11	193.7916	190.5578	187.3599
-28	1593.9380	1534.4770	1476.9663	12	184.7142	181.6319	178.5838
-27	1500.8930	1444.9030	1390.7495	13	176.1211	173.1822	170.2759
-26	1413.9673	1361.2200	1310.2028	14	167.9835	165.1804	162.4083
-25	1329.1367	1283.0000	1238.3016	15	159.8962	157.6000	155.3211
-24	1252.8144	1209.3270	1167.1953	16	152.6170	150.4253	148.2502
-23	1181.4336	1140.4240	1100.6928	17	145.7160	143.6234	141.5466
-22	1114.6401	1075.9490	1038.4640	18	139.1712	137.1726	135.1891
-21	1052.1085	1015.5880	980.2059	19	132.9622	131.0528	129.1578
-20	990.8617	959.0500	928.1695	20	126.7793	125.2450	123.7170
-19	936.0641	906.0117	876.8390	21	121.1240	119.6582	118.1984
-18	884.6914	856.2883	828.7167	22	115.7568	114.3559	112.9608
-17	836.5067	809.6506	783.5807	23	110.6613	109.3221	107.9884
-16	791.2910	765.8865	741.2257	24	105.6340	104.5415	103.4506
-15	746.7035	724.8000	703.4662	25	101.0000	100.0000	99.0000
-14	706.3711	685.6507	665.4693	26	96.8195	95.8191	94.8195
-13	668.5024	648.8929	629.7934	27	92.8384	91.8392	90.8415
-12	632.9310	614.3649	596.2817	28	89.0074	88.0494	87.0928
-11	599.5024	581.9169	564.7888	29	85.3582	84.4395	83.5222
-10	566.5131	551.4100	536.6581	30	81.9874	81.0000	80.0167
-9	537.0073	522.6908	508.7073	31	78.5700	77.6238	76.6815
-8	509.2437	495.6674	482.4068	32	75.3161	74.4091	73.5058
-7	483.1082	470.2286	457.6486	33	72.2170	71.3472	70.4812
-6	458.4948	446.2714	434.3323	34	69.2643	68.4301	67.5994
-5	434.1442	423.7000	413.4672	35	66.5901	65.6500	64.7167
-4	411.9667	402.0560	392.3459	36	63.8857	62.9838	62.0884
-3	391.0739	381.6658	372.4482	37	61.3075	60.4420	59.5827
-2	371.3832	362.4488	353.6953	38	58.8489	58.0181	57.1933
-1	352.8178	344.3301	336.0142	39	56.5037	55.7060	54.9141
0	334.4393	327.2400	320.1638	40	54.3774	53.5000	52.6316
1	317.8826	311.0397	304.3138	41	52.2133	51.3708	50.5370
2	302.2571	295.7506	289.3553	42	50.1482	49.3391	48.5382
3	287.5046	281.3157	275.2325	43	48.1771	47.3998	46.6304
4	273.5710	267.6820	261.8936	44	46.2952	45.5483	44.8090
5	259.7533	254.8000	249.9157	45	44.5864	43.7800	42.9836
6	247.2985	242.5827	237.9326	46	42.8302	42.0555	41.2905
7	235.5234	231.0321	226.6034	47	41.1535	40.4092	39.6741
8	224.3869	220.1080	215.8887	48	39.5522	38.8369	38.1304

9	213.8504	209.7724	205.7513	49	38.0227	37.3350	36.6558

R---T TABLE

R25℃=100KΩ ±1%　　B25℃/50℃=3950±1%

T	Rmax	Rnor	Rmin	T	Rmax	Rnor	Rmin
50	36.6326	35.8999	35.1785	90	9.4159	9.1000	8.7939
51	35.3225	34.6160	33.9204	91	9.1231	8.8171	8.5205
52	34.0669	33.3855	32.7147	92	8.8410	8.5444	8.2571
53	32.8632	32.2059	31.5588	93	8.5691	8.2816	8.0031
54	31.7091	31.0748	30.4504	94	8.3069	8.0283	7.7583
55	30.6600	29.9900	29.3318	95	8.0667	7.7840	7.5105
56	29.5510	28.9053	28.2709	96	7.8282	7.5538	7.2884
57	28.4885	27.8660	27.2545	97	7.5979	7.3316	7.0740
58	27.4703	26.8700	26.2803	98	7.3756	7.1172	6.8670
59	26.4943	25.9153	25.3466	99	7.1610	6.9100	6.6672
60	25.6058	25.0000	24.4063	100	6.9643	6.7100	6.4644
61	24.6941	24.1099	23.5373	101	6.7739	6.5265	6.2876
62	23.8200	23.2565	22.7042	102	6.5896	6.3490	6.1166
63	22.9818	22.4381	21.9052	103	6.4113	6.1772	5.9511
64	22.1778	21.6531	21.1389	104	6.2388	6.0109	5.7909
65	21.4449	20.9000	20.3669	105	6.0808	5.8500	5.6274
66	20.7000	20.1741	19.6595	106	5.9075	5.6832	5.4669
67	19.9852	19.4774	18.9806	107	5.7400	5.5221	5.3119
68	19.2990	18.8087	18.3289	108	5.5780	5.3663	5.1620
69	18.6402	18.1666	17.7032	109	5.4214	5.2156	5.0171
70	18.0391	17.5500	17.0723	110	5.2777	5.0700	4.8700
71	17.4182	16.9459	16.4847	111	5.1310	4.9291	4.7346
72	16.8220	16.3659	15.9204	112	4.9892	4.7928	4.6037
73	16.2495	15.8089	15.3786	113	4.8519	4.6610	4.4771
74	15.6996	15.2739	14.8582	114	4.7192	4.5334	4.3546
75	15.1975	14.7600	14.3337	115	4.5973	4.4100	4.2299
76	14.7046	14.2813	13.8688	116	4.4729	4.2906	4.1154
77	14.2303	13.8206	13.4215	117	4.3524	4.1751	4.0046
78	13.7739	13.3774	12.9910	118	4.2358	4.0632	3.8973
79	13.3346	12.9507	12.5767	119	4.1229	3.9549	3.7934
80	12.9333	12.5400	12.1575	120	4.0192	3.8500	3.6877
81	12.5153	12.1347	11.7646	121	3.9054	3.7410	3.5833
82	12.1130	11.7447	11.3865	122	3.7954	3.6357	3.4824
83	11.7259	11.3694	11.0225	123	3.6891	3.5338	3.3848
84	11.3532	11.0080	10.6722	124	3.5862	3.4353	3.2904
85	11.0123	10.6600	10.3179	125	3.4915	3.3400	3.1947

86	10.6655	10.3243	9.9930	126	3.4027	3.2550	3.1135
87	10.3315	10.0010	9.6800	127	3.3166	3.1726	3.0346
88	10.0097	9.6895	9.3785	128	3.2330	3.0927	2.9582
89	9.6996	9.3893	9.0880	129	3.1520	3.0152	2.8841

R---T TABLE

R25℃=100KΩ ±1% **B25℃/50℃=3950±1%**

T	Rmax	Rnor	Rmin	T	Rmax	Rnor	Rmin
130	3.0776	2.9400	2.8084	170	1.1861	1.1220	1.0613
131	2.9974	2.8634	2.7353	171	1.1582	1.0956	1.0363
132	2.9198	2.7893	2.6644	172	1.1310	1.0699	1.0120
133	2.8445	2.7173	2.5957	173	1.1046	1.0449	0.9884
134	2.7715	2.6476	2.5291	174	1.0790	1.0206	0.9654
135	2.7043	2.5800	2.4613	175	1.0551	0.9970	0.9419
136	2.6355	2.5144	2.3987	176	1.0326	0.9757	0.9218
137	2.5688	2.4507	2.3380	177	1.0107	0.9550	0.9023
138	2.5041	2.3890	2.2791	178	0.9893	0.9348	0.8832
139	2.4413	2.3291	2.2220	179	0.9685	0.9151	0.8646
140	2.3834	2.2710	2.1637	180	0.9493	0.8960	0.8457
141	2.3231	2.2135	2.1089	181	0.9271	0.8750	0.8258
142	2.2645	2.1577	2.0557	182	0.9056	0.8547	0.8067
143	2.2077	2.1035	2.0041	183	0.8846	0.8349	0.7880
144	2.1525	2.0510	1.9541	184	0.8643	0.8157	0.7699
145	2.1016	2.0000	1.9031	185	0.8453	0.7970	0.7514
146	2.0504	1.9513	1.8567	186	0.8279	0.7806	0.7360
147	2.0007	1.9040	1.8117	187	0.8110	0.7646	0.7209
148	1.9524	1.8580	1.7680	188	0.7944	0.7490	0.7062
149	1.9055	1.8134	1.7255	189	0.7783	0.7338	0.6918
150	1.8624	1.7700	1.6822	190	0.7633	0.7190	0.6771
151	1.8222	1.7319	1.6459	191	0.7463	0.7029	0.6619
152	1.7832	1.6947	1.6106	192	0.7297	0.6873	0.6472
153	1.7451	1.6586	1.5763	193	0.7136	0.6721	0.6329
154	1.7080	1.6233	1.5428	194	0.6979	0.6574	0.6191
155	1.6739	1.5890	1.5083	195	0.6835	0.6430	0.6049
156	1.6349	1.5520	1.4732	196	0.6698	0.6302	0.5928
157	1.5970	1.5160	1.4391	197	0.6566	0.6177	0.5811
158	1.5602	1.4811	1.4059	198	0.6436	0.6055	0.5696
159	1.5244	1.4471	1.3736	199	0.6309	0.5936	0.5584
160	1.4914	1.4140	1.3406	200	0.6192	0.5820	0.5469
161	1.4568	1.3812	1.3095	201	0.6082	0.5717	0.5373
162	1.4233	1.3494	1.2793	202	0.5976	0.5617	0.5279

163	1.3906	1.3184	1.2500	203	0.5872	0.5519	0.5187
164	1.3588	1.2883	1.2214	204	0.5769	0.5423	0.5096
165	1.3294	1.2590	1.1923	205	0.5677	0.5330	0.5004
166	1.2988	1.2301	1.1649	206	0.5565	0.5225	0.4905
167	1.2691	1.2019	1.1382	207	0.5455	0.5122	0.4808
168	1.2402	1.1745	1.1123	208	0.5349	0.5022	0.4715
169	1.2121	1.1479	1.0871	209	0.5245	0.4925	0.4624

R---T TABLE

R25℃=100KΩ ±1% **B25℃/50℃=3950±1%**

T	Rmax	Rnor	Rmin	T	Rmax	Rnor	Rmin
210	0.5149	0.4830	0.4529	250	0.2471	0.2300	0.2141
211	0.5046	0.4733	0.4438	251	0.2430	0.2262	0.2106
212	0.4945	0.4639	0.4350	252	0.2388	0.2223	0.2070
213	0.4847	0.4547	0.4264	253	0.2348	0.2186	0.2035
214	0.4751	0.4457	0.4180	254	0.2309	0.2150	0.2002
215	0.4664	0.4370	0.4095	255	0.2273	0.2114	0.1966
216	0.4572	0.4284	0.4014	256	0.2235	0.2079	0.1934
217	0.4482	0.4200	0.3935	257	0.2199	0.2045	0.1902
218	0.4395	0.4118	0.3859	258	0.2162	0.2011	0.1871
219	0.4309	0.4038	0.3784	259	0.2127	0.1978	0.1840
220	0.4230	0.3960	0.3707	260	0.2094	0.1946	0.1808
221	0.4149	0.3884	0.3636	261	0.2060	0.1914	0.1779
222	0.4070	0.3810	0.3566	262	0.2026	0.1883	0.1750
223	0.3994	0.3739	0.3500	263	0.1994	0.1853	0.1722
224	0.3918	0.3668	0.3434	264	0.1962	0.1823	0.1694
225	0.3850	0.3600	0.3366	265	0.1932	0.1794	0.1666
226	0.3778	0.3533	0.3304	266	0.1901	0.1765	0.1639
227	0.3708	0.3467	0.3242	267	0.1871	0.1737	0.1613
228	0.3639	0.3403	0.3182	268	0.1842	0.1710	0.1588
229	0.3573	0.3341	0.3124	269	0.1813	0.1683	0.1563
230	0.3510	0.3280	0.3065	270	0.1785	0.1656	0.1536
231	0.3446	0.3220	0.3009	271	0.1757	0.1630	0.1512
232	0.3383	0.3161	0.2953	272	0.1729	0.1604	0.1488
233	0.3322	0.3104	0.2900	273	0.1702	0.1579	0.1465
234	0.3262	0.3048	0.2848	274	0.1676	0.1555	0.1443
235	0.3206	0.2993	0.2794	275	0.1652	0.1531	0.1419
236	0.3149	0.2940	0.2744	276	0.1626	0.1507	0.1397
237	0.3094	0.2888	0.2696	277	0.1601	0.1484	0.1375
238	0.3038	0.2836	0.2647	278	0.1576	0.1461	0.1354
239	0.2984	0.2786	0.2601	279	0.1553	0.1439	0.1334

240	0.2934	0.2737	0.2553	280	0.1530	0.1417	0.1312
241	0.2883	0.2689	0.2508	281	0.1508	0.1396	0.1293
242	0.2832	0.2642	0.2464	282	0.1485	0.1375	0.1273
243	0.2783	0.2596	0.2421	283	0.1462	0.1354	0.1254
244	0.2735	0.2551	0.2379	284	0.1441	0.1334	0.1235
245	0.2690	0.2507	0.2337	285	0.1420	0.1314	0.1216
246	0.2644	0.2464	0.2296	286	0.1400	0.1295	0.1198
247	0.2599	0.2422	0.2257	287	0.1378	0.1275	0.1180
248	0.2554	0.2380	0.2218	288	0.1359	0.1257	0.1163
249	0.2511	0.2340	0.2181	289	0.1338	0.1238	0.1145

R---T TABLE

R25℃=100KΩ ±1%　　　　**B25℃/50℃=3950±1%**

T	Rmax	Rnor	Rmin
290	0.1320	0.1220	0.1128
291	0.1301	0.1202	0.1111
292	0.1282	0.1185	0.1095
293	0.1264	0.1168	0.1080
294	0.1245	0.1151	0.1064
295	0.1228	0.1134	0.1047
296	0.1211	0.1118	0.1033
297	0.1193	0.1102	0.1018
298	0.1176	0.1086	0.1003
299	0.1160	0.1071	0.0989
300	0.1145	0.1056	0.0974
