# Supplementary material for: Thermal and optical behavior dataset of surfaces coated with high reflectance and common materials under different conditions, used in Brazil
Source: Data Brief. 2020 Mar 19;30:105445. doi: 10.1016/j.dib.2020.105445 (PMC7132077; doi:10.1016/j.dib.2020.105445)
Supplement: Supplementary file 3 [file mmc3.pdf]

Arduino MEGA 2560

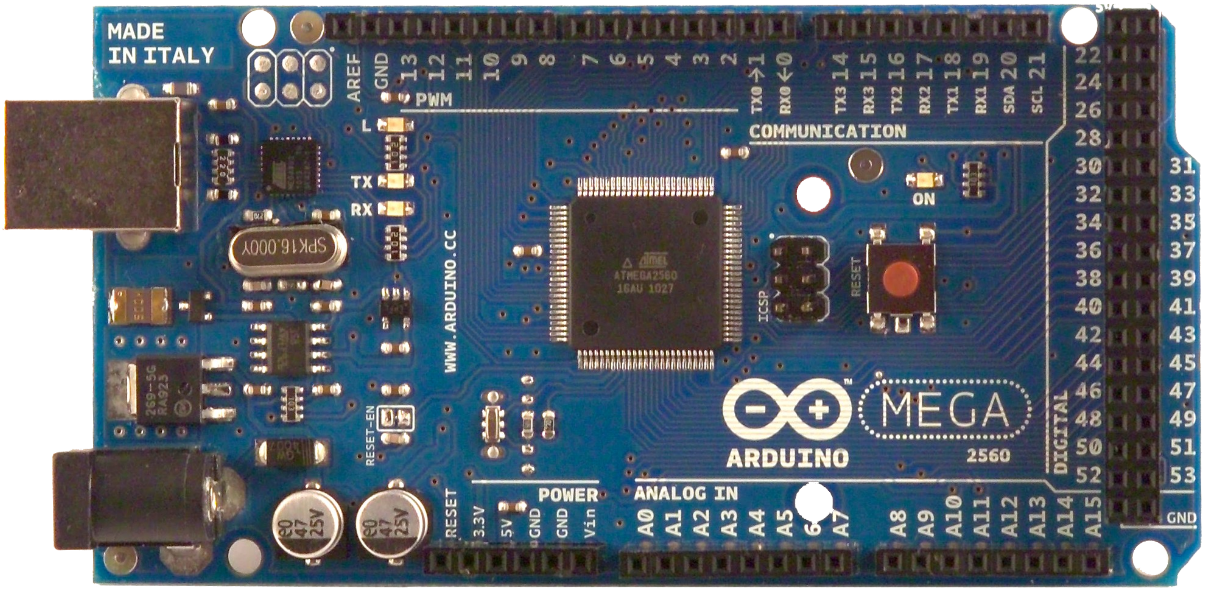

Product Overview

The Arduino Mega 2560 is a microcontroller board based on the ATmega2560 (datasheet). It has 54 digital input/output pins (of which 14 can be used as PWM outputs), 16 analog inputs, 4 UARTs (hardware serial ports), a 16 MHz crystal oscillator, a USB connection, a power jack, an ICSP header, and a reset button. It contains everything needed to support the microcontroller; simply connect it to a computer with a USB cable or power it with a AC-to-DC adapter or battery to get started. The Mega is compatible with most shields designed for the Arduino Duemilanove or Diecimila.

Index

Technical Specifications — Page 2

How to use Arduino
Programming Enviroment, Basic Tutorials — Page 6

Terms & Conditions — Page 7

Enviromental Policies
half sqm of green via Impatto Zero® — Page 7

radiospares RADIONICS ALLIED ELECTRONICS

Technical Specification

EAGLE files: arduino-mega2560-reference-design.zip Schematic: arduino-mega2560-schematic.pdf

Summary

Microcontroller	ATmega2560
Operating Voltage	5V
Input Voltage (recommended)	7-12V
Input Voltage (limits)	6-20V
Digital I/O Pins	54 (of which 14 provide PWM output)
Analog Input Pins	16
DC Current per I/O Pin	40 mA
DC Current for 3.3V Pin	50 mA
Flash Memory	256 KB of which 8 KB used by bootloader
SRAM	8 KB
EEPROM	4 KB
Clock Speed	16 MHz

the board

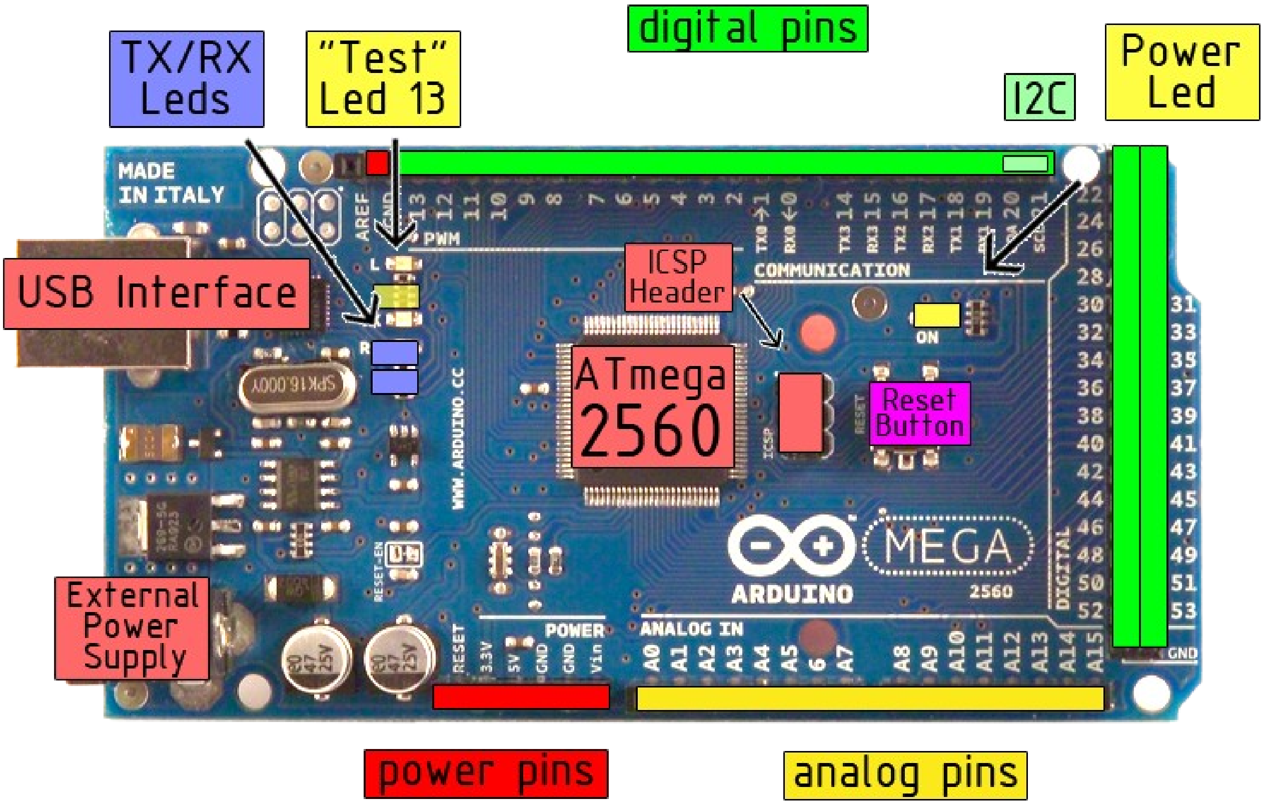

Power

The Arduino Mega2560 can be powered via the USB connection or with an external power supply. The power source is selected automatically. External (non-USB) power can come either from an AC-to-DC adapter (wall-wart) or battery. The adapter can be connected by plugging a 2.1mm center-positive plug into the board's power jack. Leads from a battery can be inserted in the Gnd and Vin pin headers of the POWER connector.

The board can operate on an external supply of 6 to 20 volts. If supplied with less than 7V, however, the 5V pin may supply less than five volts and the board may be unstable. If using more than 12V, the voltage regulator may overheat and damage the board. The recommended range is 7 to 12 volts.

The Mega2560 differs from all preceding boards in that it does not use the FTDI USB-to-serial driver chip. Instead, it features the Atmega8U2 programmed as a USB-to-serial converter.

The power pins are as follows:

- **VIN.** The input voltage to the Arduino board when it's using an external power source (as opposed to 5 volts from the USB connection or other regulated power source). You can supply voltage through this pin, or, if supplying voltage via the power jack, access it through this pin.
- **5V.** The regulated power supply used to power the microcontroller and other components on the board. This can come either from VIN via an on-board regulator, or be supplied by USB or another regulated 5V supply.
- **3V3.** A 3.3 volt supply generated by the on-board regulator. Maximum current draw is 50 mA.
- **GND.** Ground pins.

Memory

The ATmega2560 has 256 KB of flash memory for storing code (of which 8 KB is used for the bootloader), 8 KB of SRAM and 4 KB of EEPROM (which can be read and written with the EEPROM library).

Input and Output

Each of the 54 digital pins on the Mega can be used as an input or output, using pinMode(), digitalWrite(), and digitalRead() functions. They operate at 5 volts. Each pin can provide or receive a maximum of 40 mA and has an internal pull-up resistor (disconnected by default) of 20-50 kOhms. In addition, some pins have specialized functions:

- **Serial: 0 (RX) and 1 (TX); Serial 1: 19 (RX) and 18 (TX); Serial 2: 17 (RX) and 16 (TX); Serial 3: 15 (RX) and 14 (TX).** Used to receive (RX) and transmit (TX) TTL serial data. Pins 0 and 1 are also connected to the corresponding pins of the ATmega8U2 USB-to-TTL Serial chip .
- **External Interrupts: 2 (interrupt 0), 3 (interrupt 1), 18 (interrupt 5), 19 (interrupt 4), 20 (interrupt 3), and 21 (interrupt 2).** These pins can be configured to trigger an interrupt on a low value, a rising or falling edge, or a change in value. See the attachInterrupt() function for details.
- **PWM: 0 to 13.** Provide 8-bit PWM output with the analogWrite() function.
- **SPI: 50 (MISO), 51 (MOSI), 52 (SCK), 53 (SS).** These pins support SPI communication, which, although provided by the underlying hardware, is not currently included in the Arduino language. The SPI pins are also broken out on the ICSP header, which is physically compatible with the Duemilanove and Diecimila.
- **LED: 13.** There is a built-in LED connected to digital pin 13. When the pin is HIGH value, the LED is on, when the pin is LOW, it's off.
- **I^2C: 20 (SDA) and 21 (SCL).** Support I^2C (TWI) communication using the Wire library (documentation on the Wiring website). Note that these pins are not in the same location as the I^2C pins on the Duemilanove.

The Mega2560 has 16 analog inputs, each of which provide 10 bits of resolution (i.e. 1024 different values). By default they measure from ground to 5 volts, though is it possible to change the upper end of their range using the AREF pin and analogReference() function.

There are a couple of other pins on the board:

- **AREF.** Reference voltage for the analog inputs. Used with analogReference().
- **Reset.** Bring this line LOW to reset the microcontroller. Typically used to add a reset button to shields which block the one on the board.

Communication

The Arduino Mega2560 has a number of facilities for communicating with a computer, another Arduino, or other microcontrollers. The ATmega2560 provides four hardware UARTs for TTL (5V) serial communication. An ATmega8U2 on the board channels one of these over USB and provides a virtual com port to software on the computer (Windows machines will need a .inf file, but OSX and Linux machines will recognize the board as a COM port automatically. The Arduino software includes a serial monitor which allows simple textual data to be sent to and from the board. The RX and TX LEDs on the board will flash when data is being transmitted via the ATmega8U2 chip and USB connection to the computer (but not for serial communication on pins 0 and 1).

A SoftwareSerial library allows for serial communication on any of the Mega's digital pins.

The ATmega2560 also supports I2C (TWI) and SPI communication. The Arduino software includes a Wire library to simplify use of the I2C bus; see the documentation on the Wiring website for details. To use the SPI communication, please see the ATmega2560 datasheet.

Programming

The Arduino Mega2560 can be programmed with the Arduino software (download). For details, see the reference and tutorials.

The Atmega2560 on the Arduino Mega comes preburned with a bootloader that allows you to upload new code to it without the use of an external hardware programmer. It communicates using the original STK500 protocol (reference, C header files).

You can also bypass the bootloader and program the microcontroller through the ICSP (In-Circuit Serial Programming) header; see these instructions for details.

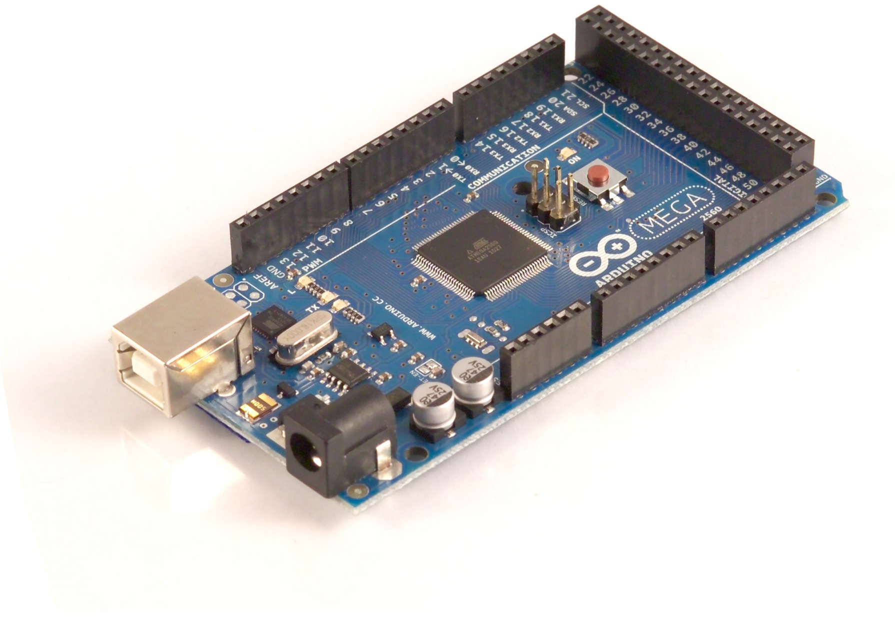

RS
radiospares
RADIONICS
ALLIED ELECTRONICS
AN ELECTROCOMPONENTS COMPANY

Automatic (Software) Reset

Rather then requiring a physical press of the reset button before an upload, the Arduino Mega2560 is designed in a way that allows it to be reset by software running on a connected computer. One of the hardware flow control lines (DTR) of the ATmega8U2 is connected to the reset line of the ATmega2560 via a 100 nanofarad capacitor. When this line is asserted (taken low), the reset line drops long enough to reset the chip. The Arduino software uses this capability to allow you to upload code by simply pressing the upload button in the Arduino environment. This means that the bootloader can have a shorter timeout, as the lowering of DTR can be well-coordinated with the start of the upload.

This setup has other implications. When the Mega2560 is connected to either a computer running Mac OS X or Linux, it resets each time a connection is made to it from software (via USB). For the following half-second or so, the bootloader is running on the Mega2560. While it is programmed to ignore malformed data (i.e. anything besides an upload of new code), it will intercept the first few bytes of data sent to the board after a connection is opened. If a sketch running on the board receives one-time configuration or other data when it first starts, make sure that the software with which it communicates waits a second after opening the connection and before sending this data.

The Mega contains a trace that can be cut to disable the auto-reset. The pads on either side of the trace can be soldered together to re-enable it. It's labeled "RESET-EN". You may also be able to disable the auto-reset by connecting a 110 ohm resistor from 5V to the reset line; see this forum thread for details.

USB Overcurrent Protection

The Arduino Mega has a resettable polyfuse that protects your computer's USB ports from shorts and overcurrent. Although most computers provide their own internal protection, the fuse provides an extra layer of protection. If more than 500 mA is applied to the USB port, the fuse will automatically break the connection until the short or overload is removed.

Physical Characteristics and Shield Compatibility

The maximum length and width of the Mega PCB are 4 and 2.1 inches respectively, with the USB connector and power jack extending beyond the former dimension. Three screw holes allow the board to be attached to a surface or case. Note that the distance between digital pins 7 and 8 is 160 mil (0.16"), not an even multiple of the 100 mil spacing of the other pins.

The Mega is designed to be compatible with most shields designed for the Diecimila or Duemilanove. Digital pins 0 to 13 (and the adjacent AREF and GND pins), analog inputs 0 to 5, the power header, and ICSP header are all in equivalent locations. Further the main UART (serial port) is located on the same pins (0 and 1), as are external interrupts 0 and 1 (pins 2 and 3 respectively). SPI is available through the ICSP header on both the Mega and Duemilanove / Diecimila. **Please note that I²C is not located on the same pins on the Mega (20 and 21) as the Duemilanove / Diecimila (analog inputs 4 and 5).**

How to use Arduino

Arduino can sense the environment by receiving input from a variety of sensors and can affect its surroundings by controlling lights, motors, and other actuators. The microcontroller on the board is programmed using the Arduino programming language (based on Wiring) and the Arduino development environment (based on Processing). Arduino projects can be stand-alone or they can communicate with software on running on a computer (e.g. Flash, Processing, MaxMSP).

Arduino is a cross-platoform program. You'll have to follow different instructions for your personal OS. Check on the Arduino site for the latest instructions. *http://arduino.cc/en/Guide/HomePage*

Linux Install

Windows Install

Mac Install

Once you have downloaded/unzipped the arduino IDE, you can Plug the Arduino to your PC via USB cable.

Blink led

Now you're actually ready to "burn" your first program on the arduino board. To select "blink led", the physical translation of the well known programming "hello world", select

File>Sketchbook> Arduino-0017>Examples> Digital>Blink

Once you have your skecth you'll see something very close to the screenshot on the right.

In **Tools>Board** select MEGA

Now you have to go to **Tools>SerialPort** and select the right serial port, the one arduino is attached to.

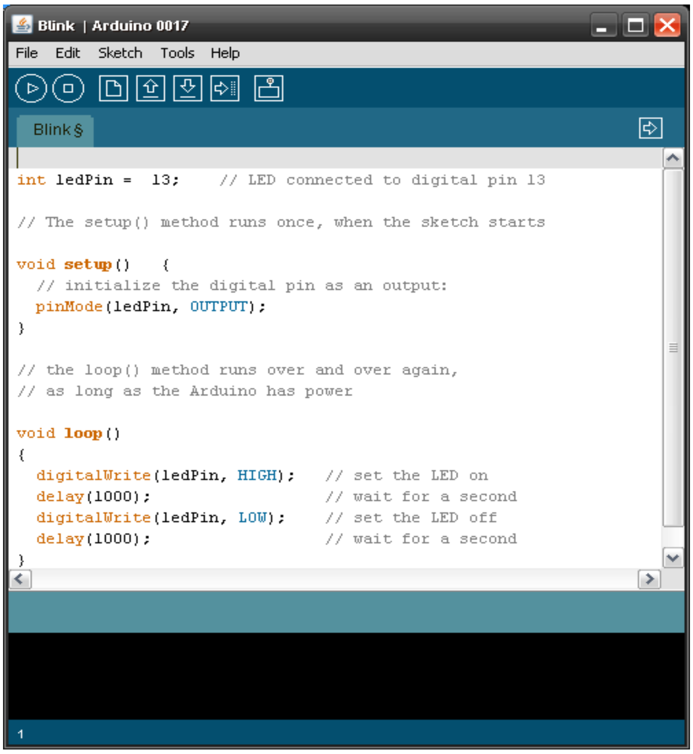

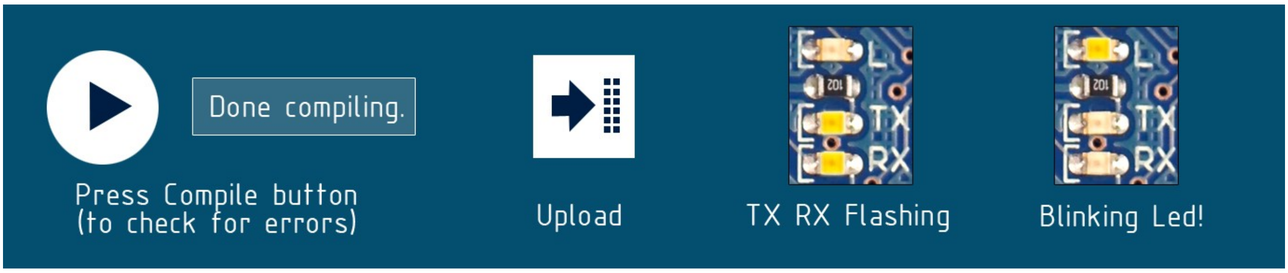

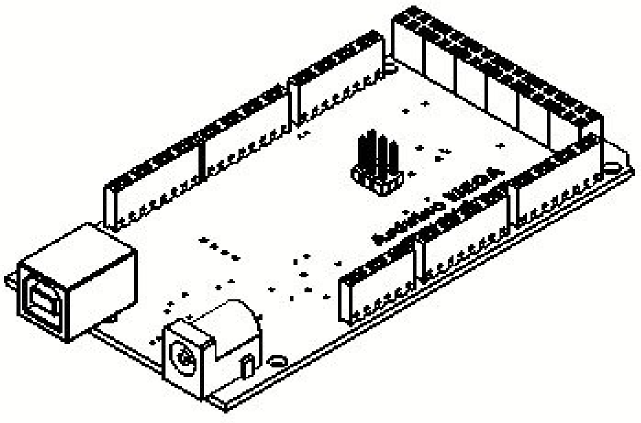

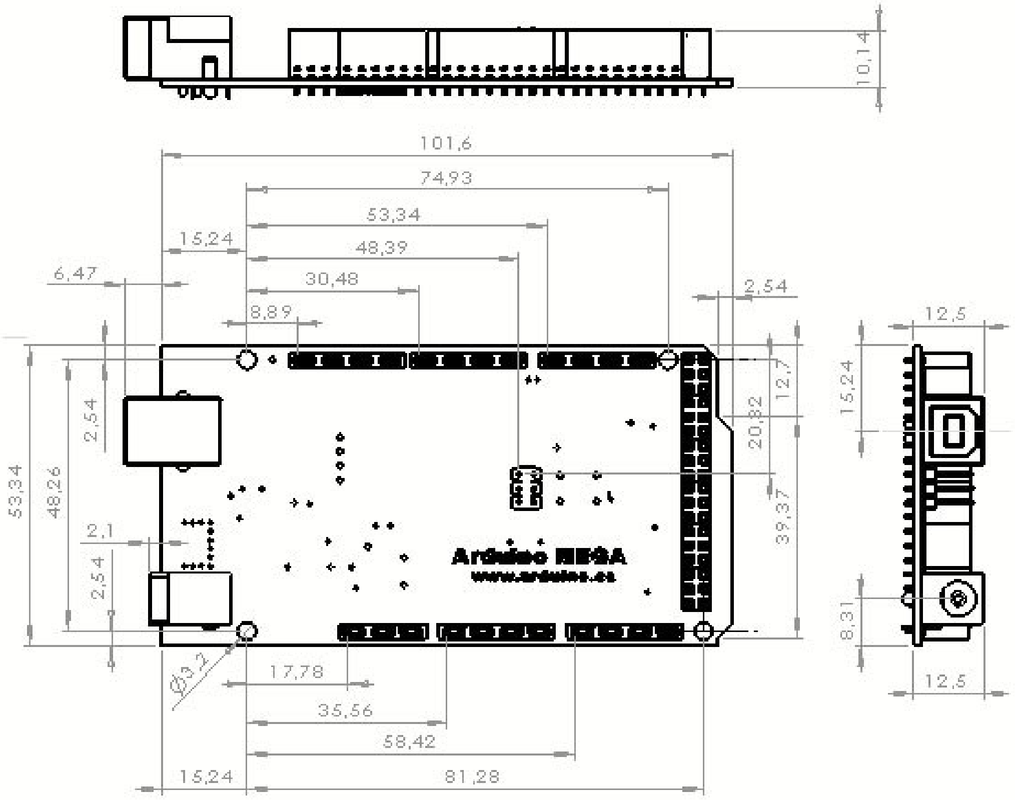

RS radiospares RADIONICS ALLIED ELECTRONICS AN ELECTROCOMPONENTS COMPANY

Terms & Conditions

1. Warranties

1.1 The producer warrants that its products will conform to the Specifications. This warranty lasts for one (1) years from the date of the sale. The producer shall not be liable for any defects that are caused by neglect, misuse or mistreatment by the Customer, including improper installation or testing, or for any products that have been altered or modified in any way by a Customer. Moreover, The producer shall not be liable for any defects that result from Customer's design, specifications or instructions for such products. Testing and other quality control techniques are used to the extent the producer deems necessary.

1.2 If any products fail to conform to the warranty set forth above, the producer's sole liability shall be to replace such products. The producer's liability shall be limited to products that are determined by the producer not to conform to such warranty. If the producer elects to replace such products, the producer shall have a reasonable time to replacements. Replaced products shall be warranted for a new full warranty period.

1.3 EXCEPT AS SET FORTH ABOVE, PRODUCTS ARE PROVIDED "AS IS" AND "WITH ALL FAULTS." THE PRODUCER DISCLAIMS ALL OTHER WARRANTIES, EXPRESS OR IMPLIED, REGARDING PRODUCTS, INCLUDING BUT NOT LIMITED TO, ANY IMPLIED WARRANTIES OF MERCHANTABILITY OR FITNESS FOR A PARTICULAR PURPOSE

1.4 Customer agrees that prior to using any systems that include the producer products, Customer will test such systems and the functionality of the products as used in such systems. The producer may provide technical, applications or design advice, quality characterization, reliability data or other services. Customer acknowledges and agrees that providing these services shall not expand or otherwise alter the producer's warranties, as set forth above, and no additional obligations or liabilities shall arise from the producer providing such services.

1.5 The Arduino™ products are not authorized for use in safety-critical applications where a failure of the product would reasonably be expected to cause severe personal injury or death. Safety-Critical Applications include, without limitation, life support devices and systems, equipment or systems for the operation of nuclear facilities and weapons systems. Arduino™ products are neither designed nor intended for use in military or aerospace applications or environments and for automotive applications or environment. Customer acknowledges and agrees that any such use of Arduino™ products which is solely at the Customer's risk, and that Customer is solely responsible for compliance with all legal and regulatory requirements in connection with such use.

1.6 Customer acknowledges and agrees that it is solely responsible for compliance with all legal, regulatory and safety-related requirements concerning its products and any use of Arduino™ products in Customer's applications, notwithstanding any applications-related information or support that may be provided by the producer.

2. Indemnification

The Customer acknowledges and agrees to defend, indemnify and hold harmless the producer from and against any and all third-party losses, damages, liabilities and expenses it incurs to the extent directly caused by: (i) an actual breach by a Customer of the representation and warranties made under this terms and conditions or (ii) the gross negligence or willful misconduct by the Customer.

3. Consequential Damages Waiver

In no event the producer shall be liable to the Customer or any third parties for any special, collateral, indirect, punitive, incidental, consequential or exemplary damages in connection with or arising out of the products provided hereunder, regardless of whether the producer has been advised of the possibility of such damages. This section will survive the termination of the warranty period.

4. Changes to specifications

The producer may make changes to specifications and product descriptions at any time, without notice. The Customer must not rely on the absence or characteristics of any features or instructions marked "reserved" or "undefined." The producer reserves these for future definition and shall have no responsibility whatsoever for conflicts or incompatibilities arising from future changes to them. The product information on the Web Site or Materials is subject to change without notice. Do not finalize a design with this information.

Enviromental Policies

The producer of Arduino™ has joined the Impatto Zero® policy of LifeGate.it. For each Arduino board produced is created / looked after half squared Km of Costa Rica's forest's.
