# Supplementary material for: Thermal and optical behavior dataset of surfaces coated with high reflectance and common materials under different conditions, used in Brazil
Source: Data Brief. 2020 Mar 19;30:105445. doi: 10.1016/j.dib.2020.105445 (PMC7132077; doi:10.1016/j.dib.2020.105445)
Supplement: Supplementary file 6 [file mmc6.pdf]

User’s manual
FLIR Ex series

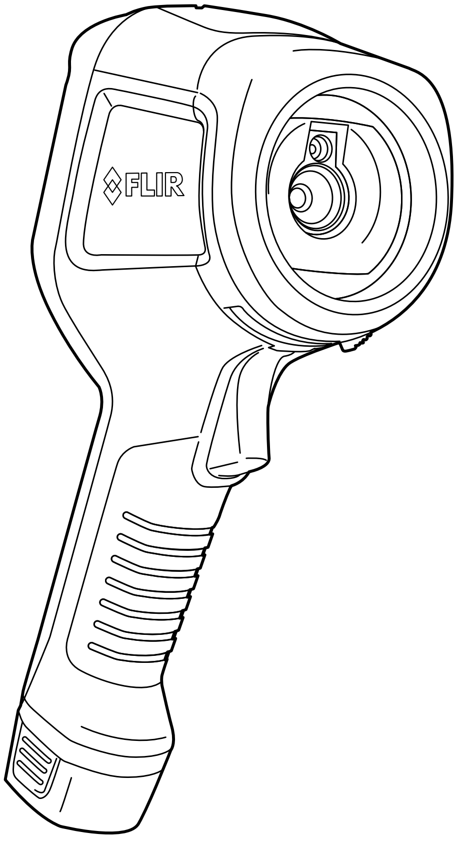

User’s manual FLIR Ex series

Table of contents

1 Disclaimers ... 1
- 1.1 Legal disclaimer ... 1
- 1.2 Usage statistics ... 1
- 1.3 Changes to registry ... 1
- 1.4 U.S. Government Regulations ... 1
- 1.5 Copyright ... 1
- 1.6 Quality assurance ... 1
- 1.7 Patents ... 1
- 1.8 EULA Terms ... 1
- 1.9 EULA Terms ... 2

2 Safety information ... 3

3 Notice to user ... 7
- 3.1 User-to-user forums ... 7
- 3.2 Calibration ... 7
- 3.3 Accuracy ... 7
- 3.4 Disposal of electronic waste ... 7
- 3.5 Training ... 7
- 3.6 Documentation updates ... 7
- 3.7 Important note about this manual ... 8
- 3.8 Note about authoritative versions ... 8

4 Customer help ... 9
- 4.1 General ... 9
- 4.2 Submitting a question ... 9
- 4.3 Downloads ... 10

5 Quick Start Guide ... 11
- 5.1 Procedure ... 11

6 List of accessories and services ... 12

7 Description ... 13
- 7.1 Camera parts ... 13
  - 7.1.1 Figure ... 13
  - 7.1.2 Explanation ... 13
- 7.2 Keypad ... 14
  - 7.2.1 Figure ... 14
  - 7.2.2 Explanation ... 14
- 7.3 Connectors ... 15
  - 7.3.1 Figure ... 15
  - 7.3.2 Explanation ... 15
- 7.4 Screen elements ... 15
  - 7.4.1 Figure ... 15
  - 7.4.2 Explanation ... 15

8 Operation ... 17
- 8.1 Charging the battery ... 17
  - 8.1.1 Charging the battery using the FLIR power supply ... 17
  - 8.1.2 Charging the battery using the FLIR stand-alone battery charger. ... 17
  - 8.1.3 Charging the battery using a USB cable ... 17
- 8.2 Turning on and turning off the camera ... 18
- 8.3 Saving an image ... 18
  - 8.3.1 General ... 18
  - 8.3.2 Image capacity ... 18

8.3.3 Naming convention 18
8.3.4 Procedure 18
8.4 Recalling an image 18
8.4.1 General 18
8.4.2 Procedure 18
8.5 Deleting an image 19
8.5.1 General 19
8.5.2 Procedure 19
8.6 Deleting all images 19
8.6.1 General 19
8.6.2 Procedure 19
8.7 Measuring a temperature using a spotmeter 19
8.7.1 General 19
8.7.2 Procedure 19
8.8 Measuring the hottest temperature within an area 20
8.8.1 General 20
8.8.2 Procedure 20
8.9 Measuring the coldest temperature within an area 20
8.9.1 General 20
8.9.2 Procedure 20
8.10 Hiding measurement tools 20
8.10.1 Procedure 20
8.11 Changing the color palette 20
8.11.1 General 20
8.11.2 Procedure 20
8.12 Working with color alarms 21
8.12.1 General 21
8.12.2 Image examples 21
8.12.3 Procedure 21
8.13 Changing image mode 22
8.13.1 General 22
8.13.2 Procedure 23
8.14 Changing the temperature scale mode 23
8.14.1 General 23
8.14.2 When to use *Manual* mode 23
8.14.3 Procedure 24
8.15 Setting the emissivity as a surface property 25
8.15.1 General 25
8.15.2 Procedure 25
8.16 Setting the emissivity as a custom material 25
8.16.1 General 25
8.16.2 Procedure 25
8.17 Changing the emissivity as a custom value 26
8.17.1 General 26
8.17.2 Procedure 26
8.18 Changing the reflected apparent temperature 26
8.18.1 General 26
8.18.2 Procedure 26
8.19 Changing the distance between the object and the camera 27
8.19.1 General 27
8.19.2 Procedure 27

8.20 Performing a non-uniformity correction (NUC) 27
8.20.1 What is a non-uniformity correction? 27
8.20.2 When to perform a non-uniformity correction? 27
8.20.3 Procedure 27
8.21 Configuring Wi-Fi 27
8.21.1 Setting up a peer-to-peer connection (most common use) 28
8.21.2 Connecting the camera to a wireless local area network (less common use) 28
8.22 Changing the settings 28
8.22.1 General 28
8.22.2 Procedure 29
8.23 Updating the camera 29
8.23.1 General 29
8.23.2 Procedure 30

9 Technical data 31
9.1 Online field-of-view calculator 31
9.2 Note about technical data 31
9.3 Note about authoritative versions 31
9.4 FLIR E4 32
9.5 FLIR E4 (incl. Wi-Fi) 35
9.6 FLIR E5 38
9.7 FLIR E5 (incl. Wi-Fi) 41
9.8 FLIR E6 45
9.9 FLIR E6 (incl. Wi-Fi) 48
9.10 FLIR E8 51
9.11 FLIR E8 (incl. Wi-Fi) 54

10 Mechanical drawings 58

11 CE Declaration of conformity 61

12 Cleaning the camera 63
12.1 Camera housing, cables, and other items 63
12.1.1 Liquids 63
12.1.2 Equipment 63
12.1.3 Procedure 63
12.2 Infrared lens 63
12.2.1 Liquids 63
12.2.2 Equipment 63
12.2.3 Procedure 63

13 Application examples 65
13.1 Moisture & water damage 65
13.1.1 General 65
13.1.2 Figure 65
13.2 Faulty contact in socket 65
13.2.1 General 65
13.2.2 Figure 66
13.3 Oxidized socket 66
13.3.1 General 66
13.3.2 Figure 66
13.4 Insulation deficiencies 67
13.4.1 General 67

13.4.2 Figure ... 67
13.5 Draft ... 68
13.5.1 General ... 68
13.5.2 Figure ... 68
14 About FLIR Systems ... 70
14.1 More than just an infrared camera ... 71
14.2 Sharing our knowledge ... 72
14.3 Supporting our customers ... 72
15 Terms, laws, and definitions ... 73
16 Thermographic measurement techniques ... 75
16.1 Introduction ... 75
16.2 Emissivity ... 75
16.2.1 Finding the emissivity of a sample ... 75
16.3 Reflected apparent temperature ... 79
16.4 Distance ... 79
16.5 Relative humidity ... 79
16.6 Other parameters ... 79
17 About calibration ... 80
17.1 Introduction ... 80
17.2 Definition—what is calibration? ... 80
17.3 Camera calibration at FLIR Systems ... 80
17.4 The differences between a calibration performed by a user and that performed directly at FLIR Systems ... 81
17.5 Calibration, verification and adjustment ... 81
17.6 Non-uniformity correction ... 82
17.7 Thermal image adjustment (thermal tuning) ... 82
18 History of infrared technology ... 83
19 Theory of thermography ... 86
19.1 Introduction ... 86
19.2 The electromagnetic spectrum ... 86
19.3 Blackbody radiation ... 87
19.3.1 Planck’s law ... 88
19.3.2 Wien’s displacement law ... 89
19.3.3 Stefan-Boltzmann's law ... 90
19.3.4 Non-blackbody emitters ... 91
19.4 Infrared semi-transparent materials ... 93
20 The measurement formula ... 94
21 Emissivity tables ... 98
21.1 References ... 98
21.2 Tables ... 98

1 Disclaimers

1.1 Legal disclaimer

All products manufactured by FLIR Systems are warranted against defective materials and workmanship for a period of one (1) year from the delivery date of the original purchase, provided such products have been under normal storage, use and service, and in accordance with FLIR Systems instruction.

Uncooled handheld infrared cameras manufactured by FLIR Systems are warranted against defective materials and workmanship for a period of two (2) years from the delivery date of the original purchase, provided such products have been under normal storage, use and service, and in accordance with FLIR Systems instruction, and provided that the camera has been registered within 60 days of original purchase.

Detectors for uncooled handheld infrared cameras manufactured by FLIR Systems are warranted against defective materials and workmanship for a period of ten (10) years from the delivery date of the original purchase, provided such products have been under normal storage, use and service, and in accordance with FLIR Systems instruction, and provided that the camera has been registered within 60 days of original purchase.

Products which are not manufactured by FLIR Systems but included in systems delivered by FLIR Systems to the original purchaser, carry the warranty, if any, of the particular supplier only. FLIR Systems has no responsibility whatsoever for such products.

The warranty extends only to the original purchaser and is not transferable. It is not applicable to any product which has been subjected to misuse, neglect, accident or abnormal conditions of operation. Expendable parts are excluded from the warranty.

In the case of a defect in a product covered by this warranty the product must not be further used in order to prevent additional damage. The purchaser shall promptly report any defect to FLIR Systems or this warranty will not apply.

FLIR Systems will, at its option, repair or replace any such defective product free of charge if, upon inspection, it proves to be defective in material or workmanship and provided that it is returned to FLIR Systems within the said one-year period.

FLIR Systems has no other obligation or liability for defects than those set forth above.

No other warranty is expressed or implied. FLIR Systems specifically disclaims the implied warranties of merchantability and fitness for a particular purpose.

FLIR Systems shall not be liable for any direct, indirect, special, incidental or consequential loss or damage, whether based on contract, tort or any other legal theory.

This warranty shall be governed by Swedish law.

Any dispute, controversy or claim arising out of or in connection with this warranty, shall be finally settled by arbitration in accordance with the Rules of the Arbitration Institute of the Stockholm Chamber of Commerce. The place of arbitration shall be Stockholm. The language to be used in the arbitral proceedings shall be English.

1.2 Usage statistics

FLIR Systems reserves the right to gather anonymous usage statistics to help maintain and improve the quality of our software and services.

1.3 Changes to registry

The registry entry HKEY_LOCAL_MACHINE\SYSTEM\CurrentControlSet\Control\Lsa\LmCompatibilityLevel will be automatically changed to level 2 if the FLIR Camera Monitor service detects a FLIR camera connected to the computer with a USB cable. The modification will only be executed if the camera device implements a remote network service that supports network logons.

1.4 U.S. Government Regulations

This product may be subject to U.S. Export Regulations. Please send any inquiries to exportquestions@flir.com.

1.5 Copyright

© 2016, FLIR Systems, Inc. All rights reserved worldwide. No parts of the software including source code may be reproduced, transmitted, transcribed or translated into any language or computer language in any form or by any means, electronic, magnetic, optical, manual or otherwise, without the prior written permission of FLIR Systems.

The documentation must not, in whole or part, be copied, photocopied, reproduced, translated or transmitted to any electronic medium or machine readable form without prior consent, in writing, from FLIR Systems.

Names and marks appearing on the products herein are either registered trademarks or trademarks of FLIR Systems and/or its subsidiaries. All other trademarks, trade names or company names referenced herein are used for identification only and are the property of their respective owners.

1.6 Quality assurance

The Quality Management System under which these products are developed and manufactured has been certified in accordance with the ISO 9001 standard.

FLIR Systems is committed to a policy of continuous development; therefore we reserve the right to make changes and improvements on any of the products without prior notice.

1.7 Patents

000439161; 000653423; 000726344; 000859020; 001707738; 001707746; 001707787; 001776519; 001954074; 002021543; 002021543-0002; 002058180; 002249953; 002531178; 002816785; 002816793; 011200326; 014347553; 057692; 061609; 07002405; 100414275; 101796816; 101796817; 101796818; 102334141; 1062100; 11063060001; 11517895; 1226865; 12300216; 12300224; 1285345; 1299699; 1325808; 1336775; 1391114; 1402918; 1404291; 1411581; 1415075; 1421497; 1458284; 1678485; 1732314; 17399650; 1880950; 1886650; 2007301511414; 2007303395047; 2008301285812; 2009301900619; 20100060357; 2010301761271; 2010301761303; 2010301761572; 2010305959313; 2011304423549; 2012304717443; 2012306207318; 2013302676195; 2015202354035; 2015304259171; 204465713; 204967995; 2106017; 2107799; 2115696; 2172004; 2315433; 2381417; 2794760001; 3006596; 3006597; 303330211; 4358936; 483782; 484155; 4889913; 4937897; 4995790001; 5177595; 540838; 579475; 584755; 599392; 60122153; 6020040116815; 602006006500.0; 6020080347796; 6020110003453; 615113; 615116; 664580; 664581; 665004; 665440; 67023029; 6707044; 677298; 68657; 69036179; 70022216; 70028915; 70028923; 70057990; 7034300; 710424; 7110035; 7154093; 7157705; 718801; 723605; 7237946; 7312822; 7332716; 7336823; 734803; 7544944; 7606484; 7634157; 7667198; 7809258; 7826736; 8018649; 8153971; 8212210; 8289372; 8340414; 8354639; 8384783; 8520970; 8565547; 8595689; 8599262; 8654239; 8680468; 8803093; 8823803; 8853631; 8933403; 9171361; 9191583; 9279728; 9280812; 9338352; 9423940; 9471970; 9595087; D549758.

1.8 EULA Terms

- You have acquired a device (“INFRARED CAMERA”) that includes software licensed by FLIR Systems AB from Microsoft Licensing, GP or its affiliates (“MS”). Those installed software products of MS origin, as well as associated media, printed materials, and “online” or electronic documentation (“SOFTWARE”) are protected by international intellectual property laws and treaties. The SOFTWARE is licensed, not sold. All rights reserved.
- IF YOU DO NOT AGREE TO THIS END USER LICENSE AGREEMENT (“EULA”), DO NOT USE THE DEVICE OR COPY THE SOFTWARE. INSTEAD, PROMPTLY CONTACT FLIR Systems AB FOR INSTRUCTIONS ON RETURN OF THE UNUSED DEVICE(S) FOR A REFUND. **ANY USE OF THE SOFTWARE, INCLUDING BUT NOT LIMITED TO USE ON THE DEVICE, WILL CONSTITUTE YOUR AGREEMENT TO THIS EULA (OR RATIFICATION OF ANY PREVIOUS CONSENT).**
- **GRANT OF SOFTWARE LICENSE.** This EULA grants you the following license:
  - You may use the SOFTWARE only on the DEVICE.
  - **NOT FAULT TOLERANT.** THE SOFTWARE IS NOT FAULT TOLERANT. FLIR Systems AB HAS INDEPENDENTLY DETERMINED HOW TO USE THE SOFTWARE IN THE DEVICE, AND MS HAS RELIED UPON FLIR Systems AB TO CONDUCT SUFFICIENT TESTING TO DETERMINE THAT THE SOFTWARE IS SUITABLE FOR SUCH USE.
  - **NO WARRANTIES FOR THE SOFTWARE.** THE SOFTWARE is provided “AS IS” and with all faults. THE ENTIRE RISK AS TO SATISFACTORY QUALITY, PERFORMANCE, ACCURACY, AND EFFORT (INCLUDING LACK OF NEGLIGENCE) IS WITH YOU. ALSO, THERE IS NO WARRANTY AGAINST INTERFERENCE WITH YOUR ENJOYMENT OF THE SOFTWARE OR AGAINST INFRINGEMENT. **IF YOU HAVE RECEIVED ANY WARRANTIES REGARDING THE DEVICE OR THE SOFTWARE, THOSE WARRANTIES DO NOT ORIGINATE FROM, AND ARE NOT BINDING ON, MS.**
  - No Liability for Certain Damages. **EXCEPT AS PROHIBITED BY LAW, MS SHALL HAVE NO LIABILITY FOR ANY INDIRECT, SPECIAL, CONSEQUENTIAL OR INCIDENTAL DAMAGES ARISING FROM OR IN CONNECTION WITH THE USE OR PERFORMANCE OF THE SOFTWARE. THIS LIMITATION SHALL APPLY EVEN IF ANY REMEDY FAILS OF ITS ESSENTIAL PURPOSE. IN NO EVENT SHALL MS BE LIABLE FOR ANY AMOUNT IN EXCESS OF U.S. TWO HUNDRED FIFTY DOLLARS (U.S.$250.00).**
  - **Limitations on Reverse Engineering, Decompilation, and Disassembly.** You may not reverse engineer, decompile, or disassemble the SOFTWARE, except and only to the extent that such activity is expressly permitted by applicable law notwithstanding this limitation.
  - **SOFTWARE TRANSFER ALLOWED BUT WITH RESTRICTIONS.** You may permanently transfer rights under this EULA only as part of a permanent sale or transfer of the Device, and only if the recipient agrees to this EULA. If the SOFTWARE is an upgrade, any transfer must also include all prior versions of the SOFTWARE.
  - **EXPORT RESTRICTIONS.** You acknowledge that SOFTWARE is subject to U.S. export jurisdiction. You agree to comply with all applicable international and national laws that apply to the SOFTWARE, including the U.S. Export Administration Regulations, as well as end-user, end-use and destination restrictions issued by U.S. and other governments. For additional information see http://www.microsoft.com/exporting/.

1.9 EULA Terms

Qt4 Core and Qt4 GUI, Copyright ©2013 Nokia Corporation and FLIR Systems AB. This Qt library is a free software; you can redistribute it and/or modify it under the terms of the GNU Lesser General Public License as published by the Free Software Foundation; either version 2.1 of the License, or (at your option) any later version. This library is distributed in the hope that it will be useful, but WITHOUT ANY WARRANTY; without even the implied warranty of MERCHANTABILITY or FITNESS FOR A PARTICULAR PURPOSE. See the GNU Lesser General Public License, http://www.gnu.org/licenses/lgpl-2.1.html. The source code for the libraries Qt4 Core and Qt4 GUI may be requested from FLIR Systems AB.

2 Safety information

⚠ **WARNING**

Applicability: Class B digital devices.

This equipment has been tested and found to comply with the limits for a Class B digital device, pursuant to Part 15 of the FCC Rules. These limits are designed to provide reasonable protection against harmful interference in a residential installation. This equipment generates, uses and can radiate radio frequency energy and, if not installed and used in accordance with the instructions, may cause harmful interference to radio communications. However, there is no guarantee that interference will not occur in a particular installation. If this equipment does cause harmful interference to radio or television reception, which can be determined by turning the equipment off and on, the user is encouraged to try to correct the interference by one or more of the following measures:

- Reorient or relocate the receiving antenna.
- Increase the separation between the equipment and receiver.
- Connect the equipment into an outlet on a circuit different from that to which the receiver is connected.
- Consult the dealer or an experienced radio/TV technician for help.

⚠ **WARNING**

Applicability: Digital devices subject to 15.19/RSS-210.

NOTICE: This device complies with Part 15 of the FCC Rules and with RSS-210 of Industry Canada. Operation is subject to the following two conditions:

1. this device may not cause harmful interference, and
2. this device must accept any interference received, including interference that may cause undesired operation.

⚠ **WARNING**

Applicability: Digital devices subject to 15.21.

NOTICE: Changes or modifications made to this equipment not expressly approved by FLIR Systems may void the FCC authorization to operate this equipment.

⚠ **WARNING**

Applicability: Digital devices subject to 2.1091/2.1093/OET Bulletin 65.

Radiofrequency radiation exposure Information: The radiated output power of the device is below the FCC/IC radio frequency exposure limits. Nevertheless, the device shall be used in such a manner that the potential for human contact during normal operation is minimized.

⚠ **WARNING**

Applicability: Cameras with one or more batteries.

Do not disassemble or do a modification to the battery. The battery contains safety and protection devices which, if damage occurs, can cause the battery to become hot, or cause an explosion or an ignition.

⚠ **WARNING**

Applicability: Cameras with one or more batteries.

If there is a leak from the battery and you get the fluid in your eyes, do not rub your eyes. Flush well with water and immediately get medical care. The battery fluid can cause injury to your eyes if you do not do this.

⚠ **WARNING**

Applicability: Cameras with one or more batteries.

Do not continue to charge the battery if it does not become charged in the specified charging time. If you continue to charge the battery, it can become hot and cause an explosion or ignition. Injury to persons can occur.

WARNING

Applicability: Cameras with one or more batteries.

Only use the correct equipment to remove the electrical power from the battery. If you do not use the correct equipment, you can decrease the performance or the life cycle of the battery. If you do not use the correct equipment, an incorrect flow of current to the battery can occur. This can cause the battery to become hot, or cause an explosion. Injury to persons can occur.

WARNING

Make sure that you read all applicable MSDS (Material Safety Data Sheets) and warning labels on containers before you use a liquid. The liquids can be dangerous. Injury to persons can occur.

CAUTION

Do not point the infrared camera (with or without the lens cover) at strong energy sources, for example, devices that cause laser radiation, or the sun. This can have an unwanted effect on the accuracy of the camera. It can also cause damage to the detector in the camera.

CAUTION

Do not use the camera in temperatures more than +50°C (+122°F), unless other information is specified in the user documentation or technical data. High temperatures can cause damage to the camera.

CAUTION

Applicability: Cameras with one or more batteries.

Do not attach the batteries directly to a car’s cigarette lighter socket, unless FLIR Systems supplies a specific adapter to connect the batteries to a cigarette lighter socket. Damage to the batteries can occur.

CAUTION

Applicability: Cameras with one or more batteries.

Do not connect the positive terminal and the negative terminal of the battery to each other with a metal object (such as wire). Damage to the batteries can occur.

CAUTION

Applicability: Cameras with one or more batteries.

Do not get water or salt water on the battery, or permit the battery to become wet. Damage to the batteries can occur.

CAUTION

Applicability: Cameras with one or more batteries.

Do not make holes in the battery with objects. Damage to the battery can occur.

CAUTION

Applicability: Cameras with one or more batteries.

Do not hit the battery with a hammer. Damage to the battery can occur.

CAUTION

Applicability: Cameras with one or more batteries.

Do not put your foot on the battery, hit it or cause shocks to it. Damage to the battery can occur.

CAUTION

Applicability: Cameras with one or more batteries.

Do not put the batteries in or near a fire, or into direct sunlight. When the battery becomes hot, the built-in safety equipment becomes energized and can stop the battery charging procedure. If the battery becomes hot, damage can occur to the safety equipment and this can cause more heat, damage or ignition of the battery.

CAUTION

Applicability: Cameras with one or more batteries.

Do not put the battery on a fire or increase the temperature of the battery with heat. Damage to the battery and injury to persons can occur.

CAUTION

Applicability: Cameras with one or more batteries.

Do not put the battery on or near fires, stoves, or other high-temperature locations. Damage to the battery and injury to persons can occur.

CAUTION

Applicability: Cameras with one or more batteries.

Do not solder directly onto the battery. Damage to the battery can occur.

CAUTION

Applicability: Cameras with one or more batteries.

Do not use the battery if, when you use, charge, or put the battery in storage, there is an unusual smell from the battery, the battery feels hot, changes color, changes shape, or is in an unusual condition. Speak with your sales office if one or more of these problems occurs. Damage to the battery and injury to persons can occur.

CAUTION

Applicability: Cameras with one or more batteries.

Only use a specified battery charger when you charge the battery. Damage to the battery can occur if you do not do this.

CAUTION

Applicability: Cameras with one or more batteries.

Only use a specified battery for the camera. Damage to the camera and the battery can occur if you do not do this.

CAUTION

Applicability: Cameras with one or more batteries.

The temperature range through which you can charge the battery is 0°C to +45°C (+32°F to +113°F). If you charge the battery at temperatures out of this range, it can cause the battery to become hot or to break. It can also decrease the performance or the life cycle of the battery.

CAUTION

Applicability: Cameras with one or more batteries.

The temperature range through which you can remove the electrical power from the battery is -15°C to +50°C (+5°F to +122°F), unless other information is specified in the user documentation or technical data. If you operate the battery out of this temperature range, it can decrease the performance or the life cycle of the battery.

CAUTION

Applicability: Cameras with one or more batteries.

When the battery is worn, apply insulation to the terminals with adhesive tape or equivalent materials before you discard it. Damage to the battery and injury to persons can occur if you do not do this.

CAUTION

Applicability: Cameras with one or more batteries.

Remove any water or moisture on the battery before you install it. Damage to the battery can occur if you do not do this.

CAUTION

Do not apply solvents or equivalent liquids to the camera, the cables, or other items. Damage to the battery and injury to persons can occur.

CAUTION

Be careful when you clean the infrared lens. The lens has an anti-reflective coating which is easily damaged. Damage to the infrared lens can occur.

CAUTION

Do not use too much force to clean the infrared lens. This can cause damage to the anti-reflective coating.

Note The encapsulation rating is only applicable when all the openings on the camera are sealed with their correct covers, hatches, or caps. This includes the compartments for data storage, batteries, and connectors.

3 Notice to user

3.1 User-to-user forums

Exchange ideas, problems, and infrared solutions with fellow thermographers around the world in our user-to-user forums. To go to the forums, visit:

http://forum.infraredtraining.com/

3.2 Calibration

We recommend that you send in the camera for calibration once a year. Contact your local sales office for instructions on where to send the camera.

3.3 Accuracy

For very accurate results, we recommend that you wait 5 minutes after you have started the camera before measuring a temperature.

3.4 Disposal of electronic waste

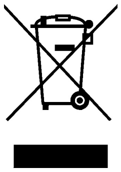

As with most electronic products, this equipment must be disposed of in an environmentally friendly way, and in accordance with existing regulations for electronic waste.

Please contact your FLIR Systems representative for more details.

3.5 Training

To read about infrared training, visit:

- http://www.infraredtraining.com
- http://www.irtraining.com
- http://www.irtraining.eu

3.6 Documentation updates

Our manuals are updated several times per year, and we also issue product-critical notifications of changes on a regular basis.

To access the latest manuals, translations of manuals, and notifications, go to the Download tab at:

http://support.flir.com

It only takes a few minutes to register online. In the download area you will also find the latest releases of manuals for our other products, as well as manuals for our historical and obsolete products.

3.7 Important note about this manual

FLIR Systems issues generic manuals that cover several cameras within a model line.

This means that this manual may contain descriptions and explanations that do not apply to your particular camera model.

3.8 Note about authoritative versions

The authoritative version of this publication is English. In the event of divergences due to translation errors, the English text has precedence.

Any late changes are first implemented in English.

4 Customer help

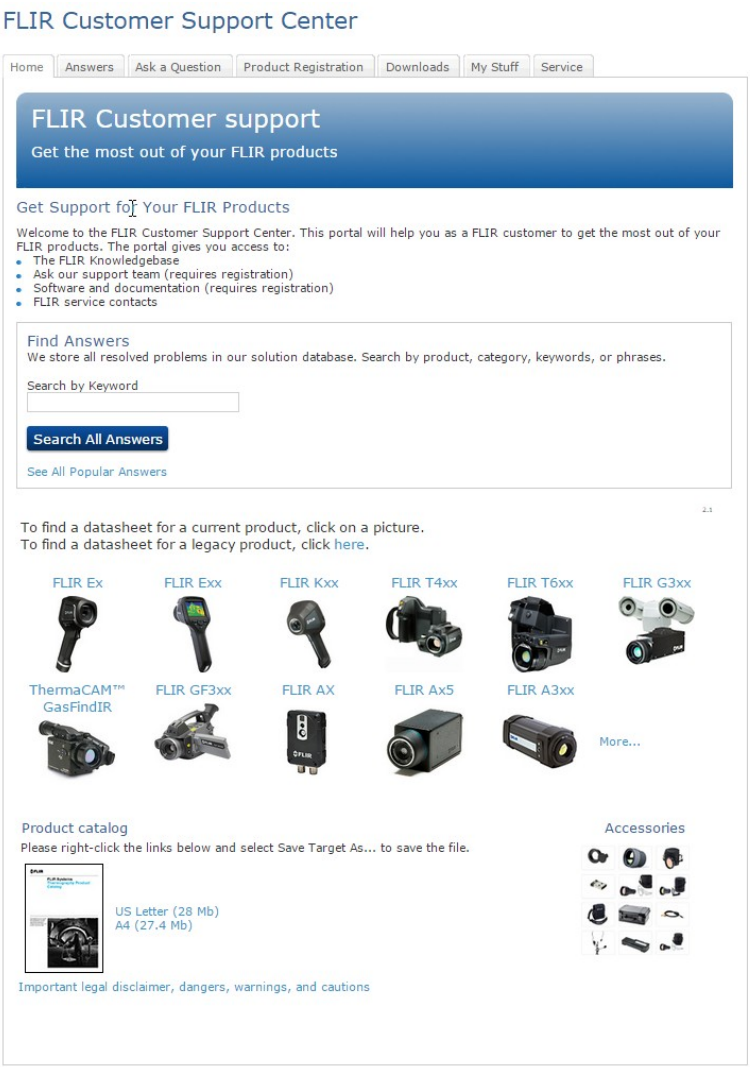

4.1 General

For customer help, visit:

http://support.flir.com

4.2 Submitting a question

To submit a question to the customer help team, you must be a registered user. It only takes a few minutes to register online. If you only want to search the knowledgebase for existing questions and answers, you do not need to be a registered user.

When you want to submit a question, make sure that you have the following information to hand:

- The camera model

- The camera serial number
- The communication protocol, or method, between the camera and your device (for example, SD card reader, HDMI, Ethernet, USB, or FireWire)
- Device type (PC/Mac/iPhone/iPad/Android device, etc.)
- Version of any programs from FLIR Systems
- Full name, publication number, and revision number of the manual

4.3 Downloads

On the customer help site you can also download the following, when applicable for the product:

- Firmware updates for your infrared camera.
- Program updates for your PC/Mac software.
- Freeware and evaluation versions of PC/Mac software.
- User documentation for current, obsolete, and historical products.
- Mechanical drawings (in *.dxf and *.pdf format).
- Cad data models (in *.stp format).
- Application stories.
- Technical datasheets.
- Product catalogs.

5 Quick Start Guide

5.1 Procedure

Follow this procedure:

1. Charge the battery. You can do this in three different ways:
   - Charge the battery using the FLIR stand-alone battery charger.
   - Charge the battery using the FLIR power supply.
   - Charge the battery using a USB cable connected to a computer.

   Note Charging the camera using a USB cable connected to a computer takes *considerably* longer than using the FLIR power supply or the FLIR stand-alone battery charger.
2. Push the On/off button ⏼ to turn on the camera.
3. Open the lens cap by pushing the lens cap lever.
4. Aim the camera toward your target of interest.
5. Pull the trigger to save an image.

(Optional steps)

6. Install FLIR Tools on your computer.
7. Start FLIR Tools.
8. Connect the camera to your computer, using the USB cable.
9. Import the images into FLIR Tools.
10. Create a PDF report in FLIR Tools.

6 List of accessories and services

Product name	Part number
Battery	T198530
Battery charger incl power supply	T198531
Car charger	T198532
FLIR Tools+ (license only)	T198583
Hard transport case FLIR Ex-series	T198528
One year extended warranty for Ex or ix series	T199806
Pouch FLIR Ex and ix series	T198529
Power supply USB-micro	T198534
Tool belt	T911093
USB cable Std A <-> Micro B	T198533

Note FLIR Systems reserves the right to discontinue models, parts or accessories, and other items, or to change specifications at any time without prior notice.

7 Description

7.1 Camera parts

7.1.1 Figure

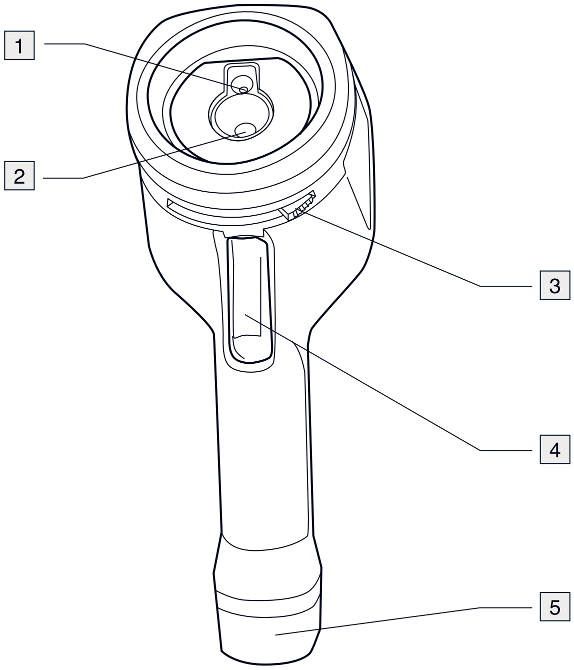

7.1.2 Explanation

1. Digital camera lens.
2. Infrared lens.
3. Lever to open and close the lens cap.
4. Trigger to save images.
5. Battery.

7.2 Keypad

7.2.1 Figure

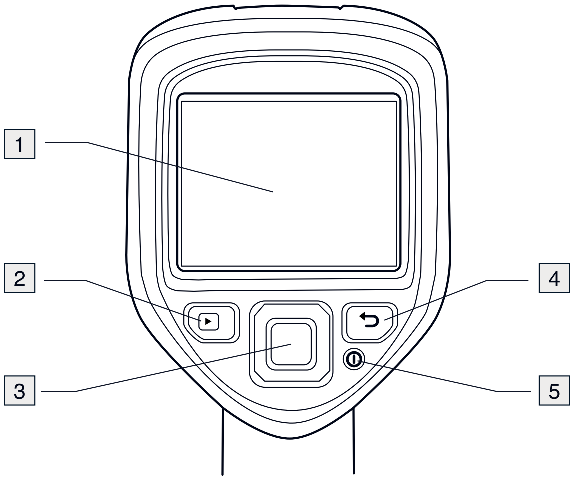

7.2.2 Explanation

1. Camera screen.
2. Archive button ▶.
   Function:
   - Push to open the image archive.
3. Navigation pad.
   Function:
   - Push left/right or up/down to navigate in menus, submenus, and dialog boxes.
   - Push the center to confirm.
4. Cancel button ↩.
   Function:
   - Push to cancel a choice.
   - Push to go back into the menu system.
5. On/off button ⏻
   Function:
   - Push the ⏻ button to turn on the camera.
   - Push and hold the ⏻ button for less than 5 seconds to put the camera in standby mode. The camera then automatically turns off after 48 hours.
   - Push and hold the ⏻ button for more than 10 seconds to turn off the camera.

7.3 Connectors

7.3.1 Figure

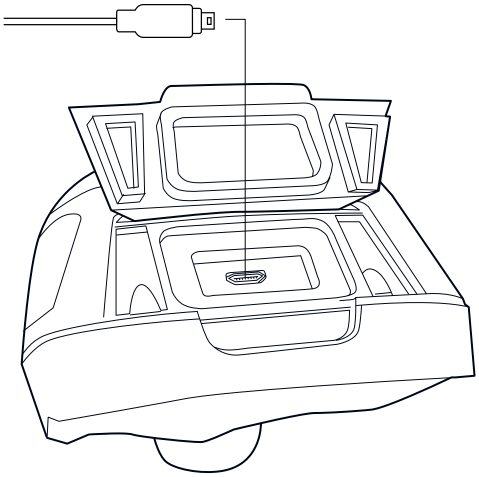

7.3.2 Explanation

The purpose of this USB mini-B connector is the following:

- Charging the battery using the FLIR power supply.
- Charging the battery using a USB cable connected to a computer.

  Note Charging the camera using a USB cable connected to a computer takes *considerably* longer than using the FLIR power supply or the FLIR stand-alone battery charger.
- Moving images from the camera to a computer for further analysis in FLIR Tools.

  Note Install FLIR Tools on your computer before you move the images.

7.4 Screen elements

7.4.1 Figure

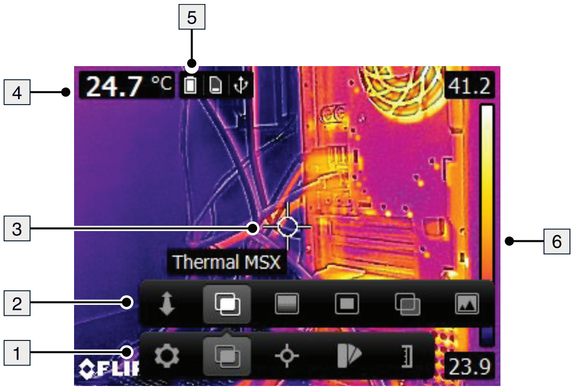

7.4.2 Explanation

1. Main menu toolbar.
2. Submenu toolbar.

3. Spotmeter.
4. Result table.
5. Status icons.
6. Temperature scale.

8 Operation

8.1 Charging the battery

WARNING
Make sure that you install the socket-outlet near the equipment and that it is easy to get access to.

8.1.1 Charging the battery using the FLIR power supply

Follow this procedure:

1. Connect the power supply to a wall outlet.
2. Connect the power supply cable to the USB connector on the camera.

NOTE
The charging time for a fully depleted battery is 2 hours.

8.1.2 Charging the battery using the FLIR stand-alone battery charger.

Follow this procedure:

1. Connect the stand-alone battery charger to a wall outlet.
2. Remove the battery from the camera.

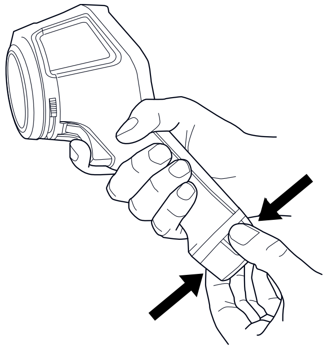

3. Put the battery into the stand-alone battery charger.

NOTE

- The charging time for a fully depleted battery is 2 hours.
- The battery is being charged when the blue LED is flashing.
- The battery is fully charged when the blue LED is continuous.

8.1.3 Charging the battery using a USB cable

Follow this procedure:

1. Connect the camera to a computer using a USB cable.

NOTE

- To charge the camera, the computer must be turned on.
- Charging the camera using a USB cable connected to a computer takes *considerably* longer than using the FLIR power supply or the FLIR stand-alone battery charger.

8.2 Turning on and turning off the camera

- Push the ⏻ button to turn on the camera.
- Push and hold the ⏻ button for less than 5 seconds to put the camera in standby mode. The camera then automatically turns off after 48 hours.
- Push and hold the ⏻ button for more than 10 seconds to turn off the camera.

8.3 Saving an image

8.3.1 General

You can save multiple images to the internal camera memory.

8.3.2 Image capacity

Approximately 500 images can be saved to the internal camera memory.

8.3.3 Naming convention

The naming convention for images is *FLIRxxxx.jpg*, where *xxxx* is a unique counter.

8.3.4 Procedure

Follow this procedure:

1. To save an image, pull the trigger.

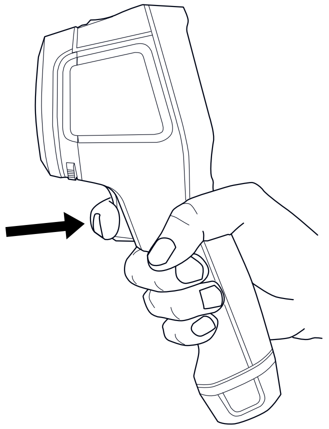

8.4 Recalling an image

8.4.1 General

When you save an image, it is stored in the internal camera memory. To display the image again, you can recall it from the internal camera memory.

8.4.2 Procedure

Follow this procedure:

1. Push the Archive button ▶.
2. Push the navigation pad left/right or up/down to select the image you want to view.
3. Push the center of the navigation pad. This displays the selected image.

4. To return to live mode, push the Cancel button repeatedly or push the Archive button .

8.5 Deleting an image

8.5.1 General

You can delete one or more images from the internal camera memory.

8.5.2 Procedure

Follow this procedure:

1. Push the Archive button .
2. Push the navigation pad left/right or up/down to select the image you want to view.
3. Push the center of the navigation pad. This displays the selected image.
4. Push the center of the navigation pad. This displays a toolbar.
5. On the toolbar, select *Delete* .

8.6 Deleting all images

8.6.1 General

You can delete all images from the internal camera memory.

8.6.2 Procedure

Follow this procedure:

1. Push the center of the navigation pad. This displays a toolbar.
2. On the toolbar, select *Settings* . This displays a dialog box.
3. In the dialog box, select *Device settings*. This displays a dialog box.
4. In the dialog box, select *Reset options*. This displays a dialog box.
5. In the dialog box, select *Delete all saved images*.

8.7 Measuring a temperature using a spotmeter

8.7.1 General

You can measure a temperature using a spotmeter. This will display the temperature at the position of the spotmeter on the screen.

8.7.2 Procedure

Follow this procedure:

1. Push the center of the navigation pad. This displays a toolbar.
2. On the toolbar, select *Measurement* . This displays a toolbar.
3. On the toolbar, select *Center spot* .
   The temperature at the position of the spotmeter will now be displayed in the top left corner of the screen.

8.8 Measuring the hottest temperature within an area

8.8.1 General

You can measure the hottest temperature within an area. This displays a moving spot-meter that indicates the hottest temperature.

8.8.2 Procedure

Follow this procedure:

1. Push the center of the navigation pad. This displays a toolbar.
2. On the toolbar, select *Measurement*. This displays a toolbar.
3. On the toolbar, select *Hot spot*.

8.9 Measuring the coldest temperature within an area

8.9.1 General

You can measure the coldest temperature within an area. This displays a moving spot-meter that indicates the coldest temperature.

8.9.2 Procedure

Follow this procedure:

1. Push the center of the navigation pad. This displays a toolbar.
2. On the toolbar, select *Measurement*. This displays a toolbar.
3. On the toolbar, select *Cold spot*.

8.10 Hiding measurement tools

8.10.1 Procedure

Follow this procedure:

1. Push the center of the navigation pad. This displays a toolbar.
2. On the toolbar, select *Measurement*. This displays a toolbar.
3. On the toolbar, select *No measurements*.

8.11 Changing the color palette

8.11.1 General

You can change the color palette that the camera uses to display different temperatures. A different palette can make it easier to analyze an image.

8.11.2 Procedure

Follow this procedure:

1. Push the center of the navigation pad. This displays a toolbar.
2. On the toolbar, select *Color*. This displays a toolbar.

3. On the toolbar, select a new color palette.

8.12 Working with color alarms

8.12.1 General

By using color alarms (isotherms), anomalies can easily be discovered in an infrared image. The isotherm command applies a contrasting color to all pixels with a temperature above or below the specified temperature level.

8.12.2 Image examples

This table explains the different color alarms (isotherms).

Color alarm	Image
Below alarm	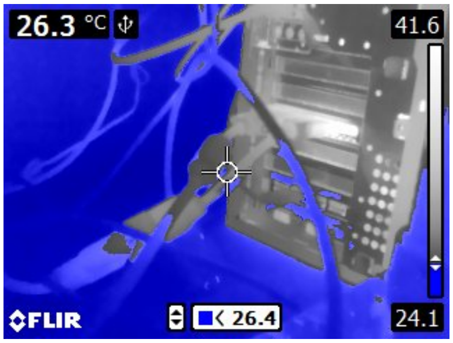
Above alarm	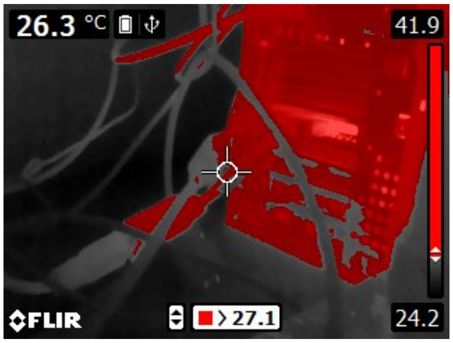

8.12.3 Procedure

Follow this procedure:

1. Push the center of the navigation pad. This displays a toolbar.
2. On the toolbar, select *Color*. This displays a toolbar.
3. On the toolbar, select the type of alarm:
   - *Below alarm*.
   - *Above alarm*.
4. Push the center of the navigation pad. The threshold temperature is displayed at the bottom of the screen.
5. To change the threshold temperature, push the navigation pad up/down.

8.13 Changing image mode

8.13.1 General

The camera can operate in five different image modes:

- *Thermal MSX* (Multi Spectral Dynamic Imaging): The camera displays an infrared image where the edges of the objects are enhanced.

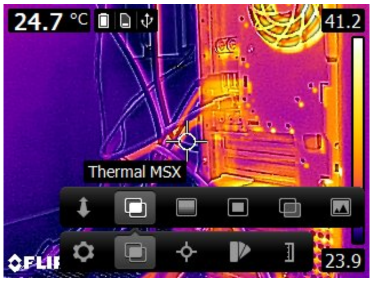

- *Thermal*: The camera displays a fully thermal image.

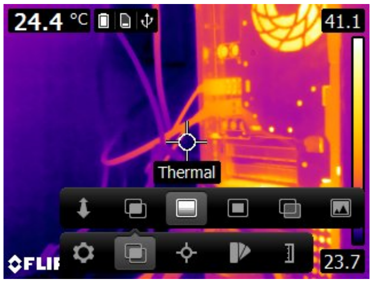

- *Picture-in-picture*: The camera displays a digital camera image with a superimposed infrared image frame.

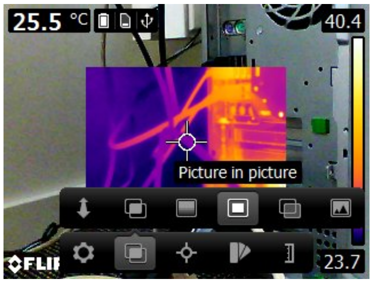

- *Thermal blending*: The camera displays a blended image that uses a mix of infrared pixels and digital photo pixels. The mixing level can be adjusted.

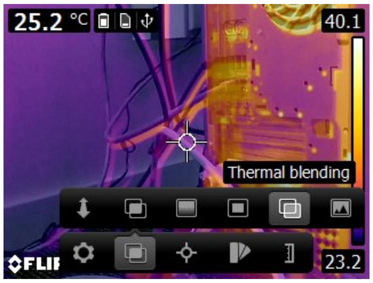

- *Digital camera*: The camera displays a digital camera image.

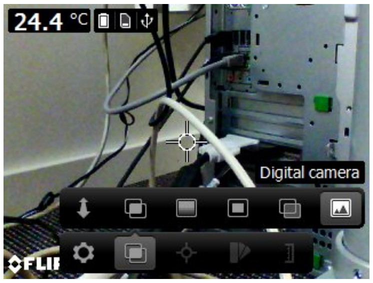

To display a good fusion image (*Thermal MSX*, *Picture-in-picture*, and *Thermal blending* modes), the camera must make adjustments to compensate for the small difference in position between the digital camera lens and the infrared lens. To adjust the image accurately, the camera requires the alignment distance (i.e., the distance to the object).

8.13.2 Procedure

Follow this procedure:

1. Push the center of the navigation pad. This displays a toolbar.
2. On the toolbar, select *Image mode*. This displays a toolbar.
3. On the toolbar, select one of the following:
   - *Thermal MSX*.
   - *Thermal*.
   - *Picture-in-picture*.
   - *Thermal blending*. This displays a dialog box where you can select the mixing level.
   - *Digital camera*.
4. If you have selected the *Thermal MSX*, *Picture-in-picture*, or *Thermal blending* mode, also set the distance to the object by doing the following:
   - On the *Image mode* toolbar, select *Alignment distance*. This displays a dialog box.
   - In the dialog box, select the distance to the object.

8.14 Changing the temperature scale mode

8.14.1 General

The camera can, depending on the camera model, operate in different temperature scale modes:

- *Auto* mode: In this mode, the camera is continuously auto-adjusted for the best image brightness and contrast.
- *Manual* mode: This mode allows manual adjustments of the temperature span and the temperature level.

8.14.2 When to use *Manual* mode

8.14.2.1 Example 1

Here are two infrared images of a building. In the left image, which is auto-adjusted, the large temperature span between the clear sky and the heated building makes a correct

analysis difficult. You can analyze the building in more detail if you change the temperature scale to values close to the temperature of the building.

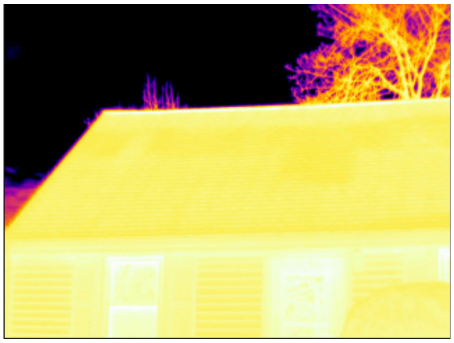
Automatic

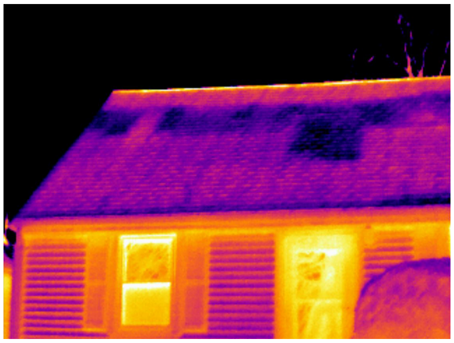
Manual

8.14.2.2 *Example 2*

Here are two infrared images of an isolator in a power line. To make it easier to analyze the temperature variations in the isolator, the temperature scale in the right image has been changed to values close to the temperature of the isolator.

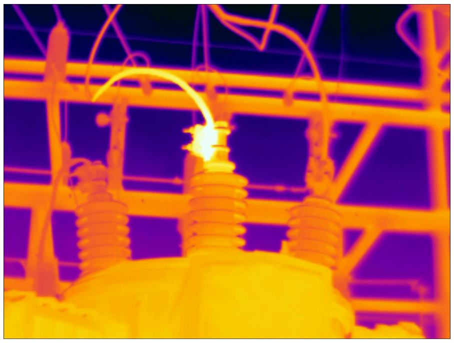
Automatic

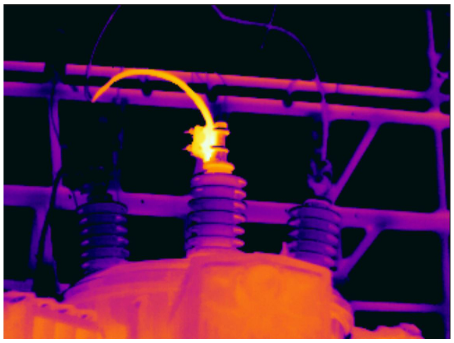
Manual

8.14.3 Procedure

Follow this procedure:

1. Push the center of the navigation pad. This displays a toolbar.
2. On the toolbar, select *Temperature scale* . This displays a toolbar.
3. On the toolbar, select one of the following:
    - *Auto* .
    - *Manual* .
4. To change the temperature span and the temperature level in *Manual* mode, do the following:
    - Push the navigation pad left/right to select (highlight) the maximum and/or minimum temperature.
    - Push the navigation pad up/down to change the value of the highlighted temperature.

8.15 Setting the emissivity as a surface property

8.15.1 General

To measure temperatures accurately, the camera must know what kind of surface you are measuring. You can choose between the following surface properties:

- *Matt*.
- *Semi-matt*.
- *Semi-glossy*.

For more information about emissivity, see section 16 *Thermographic measurement techniques*, page 75.

8.15.2 Procedure

Follow this procedure:

1. Push the center of the navigation pad. This displays a toolbar.
2. On the toolbar, select *Settings*. This displays a dialog box.
3. In the dialog box, select *Measurement parameters*. This displays a dialog box.
4. In the dialog box, select *Emissivity*. This displays a dialog box.
5. In the dialog box, select one of the following:
    - *Matt*.
    - *Semi-matt*.
    - *Semi-glossy*.

8.16 Setting the emissivity as a custom material

8.16.1 General

Instead of specifying a surface property as matt, semi-matt or semi-glossy, you can specify a custom material from a list of materials.

For more information about emissivity, see section 16 *Thermographic measurement techniques*, page 75.

8.16.2 Procedure

Follow this procedure:

1. Push the center of the navigation pad. This displays a toolbar.
2. On the toolbar, select *Settings*. This displays a dialog box.
3. In the dialog box, select *Measurement parameters*. This displays a dialog box.
4. In the dialog box, select *Emissivity*. This displays a dialog box.
5. In the dialog box, select *Custom material*. This displays a list of materials with known emissivities.
6. In the list, select the material.

8.17 Changing the emissivity as a custom value

8.17.1 General

For very precise measurements, you may need to set the emissivity, instead of selecting a surface property or a custom material. You also need to understand how emissivity and reflectivity affect measurements, rather than just simply selecting a surface property.

Emissivity is a property that indicates how much radiation originates from an object as opposed to being reflected by it. A lower value indicates that a larger proportion is being reflected, while a high value indicates that a lower proportion is being reflected.

Polished stainless steel, for example, has an emissivity of 0.14, while a structured PVC floor typically has an emissivity of 0.93.

For more information about emissivity, see section 16 *Thermographic measurement techniques*, page 75.

8.17.2 Procedure

Follow this procedure:

1. Push the center of the navigation pad. This displays a toolbar.
2. On the toolbar, select *Settings* . This displays a dialog box.
3. In the dialog box, select *Measurement parameters*. This displays a dialog box.
4. In the dialog box, select *Emissivity*. This displays a dialog box.
5. In the dialog box, select *Custom value*. This displays a dialog box where you can set a custom value.

8.18 Changing the reflected apparent temperature

8.18.1 General

This parameter is used to compensate for the radiation reflected by the object. If the emissivity is low and the object temperature significantly different from that of the reflected temperature, it will be important to set and compensate for the reflected apparent temperature correctly.

For more information about reflected apparent temperature, see section 16 *Thermographic measurement techniques*, page 75.

8.18.2 Procedure

Follow this procedure:

1. Push the center of the navigation pad. This displays a toolbar.
2. On the toolbar, select *Settings* . This displays a dialog box.
3. In the dialog box, select *Measurement parameters*. This displays a dialog box.
4. In the dialog box, select *Reflected apparent temperature*. This displays a dialog box where you can set a value.

8.19 Changing the distance between the object and the camera

8.19.1 General

To measure temperatures accurately, the camera requires the distance between the camera and the object.

8.19.2 Procedure

Follow this procedure:

1. Push the center of the navigation pad. This displays a toolbar.
2. On the toolbar, select *Settings* . This displays a dialog box.
3. In the dialog box, select *Measurement parameters*. This displays a dialog box.
4. In the dialog box, select *Distance*. This displays a dialog box where you can select a distance.

8.20 Performing a non-uniformity correction (NUC)

8.20.1 What is a non-uniformity correction?

A non-uniformity correction is *an image correction carried out by the camera software to compensate for different sensitivities of detector elements and other optical and geometrical disturbances*[1].

8.20.2 When to perform a non-uniformity correction?

The non-uniformity correction process should be carried out whenever the output image becomes spatially noisy. The output can become spatially noisy when the ambient temperature changes (such as from day to night operation, and vice versa).

8.20.3 Procedure

To perform a non-uniformity correction, push and hold the Image archive button for more than 2 seconds.

8.21 Configuring Wi-Fi

Depending on your camera configuration, you can connect the camera to a wireless local area network (WLAN) using Wi-Fi, or let the camera provide Wi-Fi access to another device.

You can connect the camera in two different ways:

- *Most common use*: Setting up a peer-to-peer connection (also called an *ad hoc* or *P2P* connection). This method is primarily used with other devices, e.g., an iPhone or iPad.
- *Less common use*: Connecting the camera to a WLAN.

1. Definition from the impending international adoption of DIN 54190-3 (Non-destructive testing – Thermographic testing – Part 3: Terms and definitions).

8.21.1 Setting up a peer-to-peer connection (most common use)

Follow this procedure:

1. Push the center of the navigation pad. This displays a toolbar.
2. On the toolbar, select *Settings* ⚙. This displays a dialog box.
3. Select *Device settings* and push the center of the navigation pad.
4. Select *Wi-Fi* and push the center of the navigation pad.
5. Select *Share* and push the center of the navigation pad.
6. (Optional step.) To display and change the parameters, select *Settings* and push the center of the navigation pad.

    - To change the channel (the channel that the camera is broadcasting on), select *Channel* and push the center of the navigation pad.
    - To activate WEP (encryption algorithm), select *WEP* and push the center of the navigation pad. This will check the *WEP* check box.
    - To change the WEP password, select *Password* and push the center of the navigation pad.

    Note These parameters are set for your camera's network. They will be used by the external device to connect that device to the network.

8.21.2 Connecting the camera to a wireless local area network (less common use)

Follow this procedure:

1. Push the center of the navigation pad. This displays a toolbar.
2. On the toolbar, select *Settings* ⚙. This displays a dialog box.
3. Select *Device settings* and push the center of the navigation pad.
4. Select *Wi-Fi* and push the center of the navigation pad.
5. Select *Connect to network* and push the center of the navigation pad.
6. To display a list of the available networks, select *Networks* and push the center of the navigation pad.
7. Select one of the available networks.
   Password-protected networks are indicated with a padlock icon, and for these you will need to enter a password.

Note Some networks do not broadcast their existence. To connect to such a network, select *Add network...* and set all parameters manually according to that network.

8.22 Changing the settings

8.22.1 General

You can change a variety of settings for the camera.

The *Settings* menu includes the following:

- *Measurement parameters*.
- *Save options*.
- *Device settings*.

8.22.1.1 Measurement parameters

- *Emissivity*.
- *Reflected temperature*.
- *Distance*.

8.22.1.2 Save options

- *Photo as separate JPEG*: When this menu command is selected, the digital photo from the visual camera is saved at its full field of view as a separate JPEG image.

8.22.1.3 Device settings

- *Language, time & units*:
  - *Language*.
  - *Temperature unit*.
  - *Distance unit*.
  - *Date & time*.
  - *Date & time format*.
- *Wi-Fi*
  - *Off*
  - *Share*
  - *Connect to network*
    - *Networks*
- *Reset options*:
  - *Reset default camera mode*.
  - *Reset device settings to factory default*.
  - *Delete all saved images*.
- *Auto power off*.
- *Display intensity*.
- *Demonstration mode*: This menu command provides a camera mode that displays various images without any user interventions. The camera mode is intended for demonstration purposes or when displaying the camera in a store.
  - *Off*.
  - *Electrical applications*.
  - *Building applications*.
- *Camera information*: This menu command displays various items of information about the camera, such as the model, serial number, and software version.

8.22.2 Procedure

Follow this procedure:

1. Push the center of the navigation pad. This displays a toolbar.
2. On the toolbar, select *Settings* 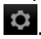. This displays a dialog box.
3. In the dialog box, select the setting that you want to change and use the navigation pad to display additional dialog boxes.

8.23 Updating the camera

8.23.1 General

To take advantage of our latest camera firmware, it is important that you keep your camera updated. You update your camera using FLIR Tools.

8.23.2 Procedure

Follow this procedure:

1. Start FLIR Tools.
2. Start the camera.
3. Connect the camera to the computer using the USB cable.
4. On the *Help* menu in FLIR Tools, click *Check for updates*.
5. Follow the on-screen instructions.

9 Technical data

Table of contents

9.1 Online field-of-view calculator 31
9.2 Note about technical data 31
9.3 Note about authoritative versions 31
9.4 FLIR E4 32
9.5 FLIR E4 (incl. Wi-Fi) 35
9.6 FLIR E5 38
9.7 FLIR E5 (incl. Wi-Fi) 41
9.8 FLIR E6 45
9.9 FLIR E6 (incl. Wi-Fi) 48
9.10 FLIR E8 51
9.11 FLIR E8 (incl. Wi-Fi) 54

9.1 Online field-of-view calculator

Please visit http://support.flir.com and click the photo of the camera series for field-of-view tables for all lens–camera combinations.

9.2 Note about technical data

FLIR Systems reserves the right to change specifications at any time without prior notice. Please check http://support.flir.com for latest changes.

9.3 Note about authoritative versions

The authoritative version of this publication is English. In the event of divergences due to translation errors, the English text has precedence.

Any late changes are first implemented in English.

9.4 FLIR E4

P/N: 63901-0101
Rev.: 41166

General description
The FLIR Ex series cameras are point-and-shoot infrared cameras that give you access to the infrared world. A FLIR Ex series camera is an affordable replacement for an infrared thermometer, providing a thermal image with temperature information in every pixel. The new MSX and visual formats make the cameras incomparably easy to use.<br>The FLIR Ex series cameras are user-friendly, compact, and rugged, for use in harsh environments. The wide field of view makes them the perfect choice for building applications.
Benefits:
• Easy to use: The FLIR Ex series cameras are fully automatic and focus-free with an intuitive interface for simple measurements in thermal, visual, or MSX mode.<br>• Compact and rugged: The FLIR Ex series cameras' low weight of 0.575 kg and the accessory belt pouch make them easy to bring along at all times. Their rugged design can withstand a 2 m drop test, and ensures reliability, even in harsh environments.<br>• Ground breaking affordability: The FLIR Ex series cameras are the most affordable infrared cameras on the market.

Imaging and optical data	
IR resolution	80 × 60 pixels
Thermal sensitivity/NETD	<0.15°C (0.27°F) / <150 mK
Field of view (FOV)	45° × 34°
Minimum focus distance	0.5 m (1.6 ft.)
Spatial resolution (IFOV)	10.3 mrad
F-number	1.5
Image frequency	9 Hz
Focus	Focus free

Detector data	
Detector type	Focal plane array (FPA), uncooled microbolometer
Spectral range	7.5–13 µm

Image presentation	
Display	3.0 in. 320 × 240 color LCD
Image adjustment	Automatic adjust/lock image

Image presentation modes	
Image modes	Thermal MSX, Thermal, Thermal blending, Digital camera.
Multi Spectral Dynamic Imaging (MSX)	IR image with enhanced detail presentation

Measurement	
Object temperature range	–20°C to +250°C (–4°F to +482°F)
Accuracy	±2°C (±3.6°F) or ±2% of reading, for ambient temperature 10°C to 35°C (+50°F to 95°F) and object temperature above +0°C (+32°F)

Measurement analysis	
Spotmeter	Center spot
Emissivity correction	Variable from 0.1 to 1.0
Emissivity table	Emissivity table of predefined materials
Reflected apparent temperature correction	Automatic, based on input of reflected temperature

Set-up	
Color palettes	Black and white, iron and rainbow
Set-up commands	Local adaptation of units, language, date and time formats

Storage of images	
File formats	Standard JPEG, 14-bit measurement data included

Digital camera	
Digital camera, resolution	640 × 480
Digital camera, FOV	55° × 43°

Data communication interfaces	
Interfaces	USB Micro: Data transfer to and from PC and Mac device

Power system	
Battery type	Rechargeable Li ion battery
Battery voltage	3.6 V
Battery operating time	Approx. 4 hours at +25°C (+77°F) ambient temperature and typical use
Charging system	Battery is charged inside the camera or in specific charger.
Charging time	2.5 hours to 90% capacity in camera. 2 hours in charger.
Power management	Automatic shut-down
AC operation	AC adapter, 90–260 VAC input, 5 VDC output to camera

Environmental data	
Operating temperature range	–15°C to +50°C (+5°F to +122°F)
Storage temperature range	–40°C to +70°C (–40°F to +158°F)
Humidity (operating and storage)	IEC 60068-2-30/24 h 95% relative humidity
EMC	• WEEE 2012/19/EC<br>• RoHs 2011/65/EC<br>• C-Tick<br>• EN 61000-6-3<br>• EN 61000-6-2<br>• FCC 47 CFR Part 15 Class B
Encapsulation	IP 54 (IEC 60529)
Shock	25 g (IEC 60068-2-27)
Vibration	2 g (IEC 60068-2-6)
Drop	2 m (6.6 ft.)

Physical data	
Camera weight, incl. battery	0.575 kg (1.27 lb.)
Camera size (L × W × H)	244 × 95 × 140 mm (9.6 × 3.7 × 5.5 in.)
Color	Black and gray

Certifications	
Certification	UL, CSA, CE, PSE and CCC

Shipping information	
Packaging, type	Cardboard box
List of contents	• Infrared camera<br>• Hard transport case<br>• Battery (inside camera)<br>• USB cable<br>• Power supply/charger with EU, UK, US and Australian plugs<br>• Printed documentation
Packaging, weight	2.9 kg (6.4 lb.)
Packaging, size	385 × 165 × 315 mm (15.2 × 6.5 × 12.4 in.)
EAN-13	4743254000995
UPC-12	845188004941
Country of origin	Estonia

Supplies & accessories:

- T911093; Tool belt
- T198528; Hard transport case FLIR Ex-series
- T198530; Battery
- T198531; Battery charger incl power supply
- T198532; Car charger
- T198534; Power supply USB-micro
- T198529; Pouch FLIR Ex and ix series
- T198533; USB cable Std A <-> Micro B
- T199362ACC; Battery Li-ion 3.6 V, 2.6 Ah, 9.4 Wh
- T198583; FLIR Tools+ (download card incl. license key)
- T199233; FLIR Atlas SDK for .NET
- T199234; FLIR Atlas SDK for MATLAB

9.5 FLIR E4 (incl. Wi-Fi)

P/N: 63906-0604
Rev.: 41166

General description
The FLIR Ex series cameras are point-and-shoot infrared cameras that give you access to the infrared world. A FLIR Ex series camera is an affordable replacement for an infrared thermometer, providing a thermal image with temperature information in every pixel. The new MSX and visual formats make the cameras incomparably easy to use.<br>The FLIR Ex series cameras are user-friendly, compact, and rugged, for use in harsh environments. The wide field of view makes them the perfect choice for building applications.
Benefits:
• Easy to use: The FLIR Ex series cameras are fully automatic and focus-free with an intuitive interface for simple measurements in thermal, visual, or MSX mode.<br>• Compact and rugged: The FLIR Ex series cameras' low weight of 0.575 kg and the accessory belt pouch make them easy to bring along at all times. Their rugged design can withstand a 2 m drop test, and ensures reliability, even in harsh environments.<br>• Ground breaking affordability: The FLIR Ex series cameras are the most affordable infrared cameras on the market.

Imaging and optical data	
IR resolution	80 × 60 pixels
Thermal sensitivity/NETD	<0.15°C (0.27°F) / <150 mK
Field of view (FOV)	45° × 34°
Minimum focus distance	0.5 m (1.6 ft.)
Spatial resolution (IFOV)	10.3 mrad
F-number	1.5
Image frequency	9 Hz
Focus	Focus free

Detector data	
Detector type	Focal plane array (FPA), uncooled microbolometer
Spectral range	7.5–13 µm

Image presentation	
Display	3.0 in. 320 × 240 color LCD
Image adjustment	Automatic adjust/lock image

Image presentation modes	
Image modes	Thermal MSX, Thermal, Picture-in-Picture, Thermal blending, Digital camera.
Multi Spectral Dynamic Imaging (MSX)	IR image with enhanced detail presentation
Picture-in-Picture	IR area on visual image

Measurement	
Object temperature range	–20°C to +250°C (–4°F to +482°F)
Accuracy	±2°C (±3.6°F) or ±2% of reading, for ambient temperature 10°C to 35°C (+50°F to 95°F) and object temperature above +0°C (+32°F)

Measurement analysis	
Spotmeter	Center spot
Area	Box with max./min.
Isotherm	Above/below/interval
Emissivity correction	Variable from 0.1 to 1.0
Emissivity table	Emissivity table of predefined materials
Reflected apparent temperature correction	Automatic, based on input of reflected temperature

Set-up	
Color palettes	Black and white, iron and rainbow
Set-up commands	Local adaptation of units, language, date and time formats

Storage of images	
File formats	Standard JPEG, 14-bit measurement data included

Digital camera	
Digital camera, resolution	640 × 480
Digital camera, FOV	55° × 43°

Data communication interfaces	
Interfaces	USB Micro: Data transfer to and from PC and Mac device
Wi-Fi	Peer-to-peer (ad hoc) or infrastructure (network)

Radio	
Wi-Fi	• Standard: 802.11 b/g/n<br>• Frequency range:<br>◦ 2400–2480 MHz<br>◦ 5150–5260 MHz<br>• Max. output power: 15 dBm

Power system	
Battery type	Rechargeable Li ion battery
Battery voltage	3.6 V
Battery operating time	Approx. 4 hours at +25°C (+77°F) ambient temperature and typical use
Charging system	Battery is charged inside the camera or in specific charger.
Charging time	2.5 hours to 90% capacity in camera. 2 hours in charger.
Power management	Automatic shut-down
AC operation	AC adapter, 90–260 VAC input, 5 VDC output to camera

Environmental data	
Operating temperature range	–15°C to +50°C (+5°F to +122°F)
Storage temperature range	–40°C to +70°C (–40°F to +158°F)
Humidity (operating and storage)	IEC 60068-2-30/24 h 95% relative humidity

Environmental data	
EMC	• WEEE 2012/19/EC<br>• RoHs 2011/65/EC<br>• C-Tick<br>• EN 61000-6-3<br>• EN 61000-6-2<br>• FCC 47 CFR Part 15 Class B
Radio spectrum	• ETSI EN 300 328<br>• FCC 47 CSR Part 15<br>• RSS-247 Issue 1
Encapsulation	IP 54 (IEC 60529)
Shock	25 g (IEC 60068-2-27)
Vibration	2 g (IEC 60068-2-6)
Drop	2 m (6.6 ft.)

Physical data	
Camera weight, incl. battery	0.575 kg (1.27 lb.)
Camera size (L × W × H)	244 × 95 × 140 mm (9.6 × 3.7 × 5.5 in.)
Color	Black and gray

Certifications	
Certification	UL, CSA, CE, PSE and CCC

Shipping information	
Packaging, type	Cardboard box
List of contents	• Infrared camera<br>• Hard transport case<br>• Battery (inside camera)<br>• USB cable<br>• Power supply/charger with EU, UK, US and Australian plugs<br>• Printed documentation
Packaging, weight	2.9 kg (6.4 lb.)
Packaging, size	385 × 165 × 315 mm (15.2 × 6.5 × 12.4 in.)
EAN-13	4743254002869
UPC-12	845188014117
Country of origin	Estonia

Supplies & accessories:

- T911093; Tool belt
- T198528; Hard transport case FLIR Ex-series
- T198530; Battery
- T198531; Battery charger incl power supply
- T198532; Car charger
- T198534; Power supply USB-micro
- T198529; Pouch FLIR Ex and ix series
- T198533; USB cable Std A <-> Micro B
- T199362ACC; Battery Li-ion 3.6 V, 2.6 Ah, 9.4 Wh
- T198583; FLIR Tools+ (download card incl. license key)
- T199233; FLIR Atlas SDK for .NET
- T199234; FLIR Atlas SDK for MATLAB

9.6 FLIR E5

P/N: 63905-0501
Rev.: 41166

General description
The FLIR Ex series cameras are point-and-shoot infrared cameras that give you access to the infrared world. A FLIR Ex series camera is an affordable replacement for an infrared thermometer, providing a thermal image with temperature information in every pixel. The new MSX and visual formats make the cameras incomparably easy to use.<br>The FLIR Ex series cameras are user-friendly, compact, and rugged, for use in harsh environments. The wide field of view makes them the perfect choice for building applications.
Benefits:
• Easy to use: The FLIR Ex series cameras are fully automatic and focus-free with an intuitive interface for simple measurements in thermal, visual, or MSX mode.<br>• Compact and rugged: The FLIR Ex series cameras' low weight of 0.575 kg and the accessory belt pouch make them easy to bring along at all times. Their rugged design can withstand a 2 m drop test, and ensures reliability, even in harsh environments.<br>• Ground breaking affordability: The FLIR Ex series cameras are the most affordable infrared cameras on the market.

Imaging and optical data	
IR resolution	120 × 90 pixels
Thermal sensitivity/NETD	<0.10°C (0.27°F) / <100 mK
Field of view (FOV)	45° × 34°
Minimum focus distance	0.5 m (1.6 ft.)
Spatial resolution (IFOV)	6.9 mrad
F-number	1.5
Image frequency	9 Hz
Focus	Focus free

Detector data	
Detector type	Focal plane array (FPA), uncooled microbolometer
Spectral range	7.5–13 µm

Image presentation	
Display	3.0 in. 320 × 240 color LCD
Image adjustment	Automatic adjust/lock image

Image presentation modes	
Image modes	Thermal MSX, Thermal, Thermal blending, Digital camera.
Multi Spectral Dynamic Imaging (MSX)	IR image with enhanced detail presentation

Measurement	
Object temperature range	–20°C to +250°C (–4°F to +482°F)
Accuracy	±2°C (±3.6°F) or ±2% of reading, for ambient temperature 10°C to 35°C (+50°F to 95°F) and object temperature above +0°C (+32°F)

Measurement analysis	
Spotmeter	Center spot
Area	Box with max./min.
Emissivity correction	Variable from 0.1 to 1.0
Emissivity table	Emissivity table of predefined materials
Reflected apparent temperature correction	Automatic, based on input of reflected temperature

Set-up	
Color palettes	Black and white, iron and rainbow
Set-up commands	Local adaptation of units, language, date and time formats

Storage of images	
File formats	Standard JPEG, 14-bit measurement data included

Digital camera	
Digital camera, resolution	640 × 480
Digital camera, FOV	55° × 43°

Data communication interfaces	
Interfaces	USB Micro: Data transfer to and from PC and Mac device

Power system	
Battery type	Rechargeable Li ion battery
Battery voltage	3.6 V
Battery operating time	Approx. 4 hours at +25°C (+77°F) ambient temperature and typical use
Charging system	Battery is charged inside the camera or in specific charger.
Charging time	2.5 hours to 90% capacity in camera. 2 hours in charger.
Power management	Automatic shut-down
AC operation	AC adapter, 90–260 VAC input, 5 VDC output to camera

Environmental data	
Operating temperature range	–15°C to +50°C (+5°F to +122°F)
Storage temperature range	–40°C to +70°C (–40°F to +158°F)
Humidity (operating and storage)	IEC 60068-2-30/24 h 95% relative humidity
EMC	• WEEE 2012/19/EC<br>• RoHs 2011/65/EC<br>• C-Tick<br>• EN 61000-6-3<br>• EN 61000-6-2<br>• FCC 47 CFR Part 15 Class B
Encapsulation	IP 54 (IEC 60529)
Shock	25 g (IEC 60068-2-27)

Environmental data	
Vibration	2 g (IEC 60068-2-6)
Drop	2 m (6.6 ft.)

Physical data	
Camera weight, incl. battery	0.575 kg (1.27 lb.)
Camera size (L × W × H)	244 × 95 × 140 mm (9.6 × 3.7 × 5.5 in.)
Color	Black and gray

Certifications	
Certification	UL, CSA, CE, PSE and CCC

Shipping information	
Packaging, type	Cardboard box
List of contents	• Infrared camera<br>• Hard transport case<br>• Battery (inside camera)<br>• USB cable<br>• Power supply/charger with EU, UK, US and Australian plugs<br>• Printed documentation
Packaging, weight	2.9 kg (6.4 lb.)
Packaging, size	385 × 165 × 315 mm (15.2 × 6.5 × 12.4 in.)
EAN-13	4743254001114
UPC-12	845188005146
Country of origin	Estonia

Supplies & accessories:

- T911093; Tool belt
- T198528; Hard transport case FLIR Ex-series
- T198530; Battery
- T198531; Battery charger incl power supply
- T198532; Car charger
- T198534; Power supply USB-micro
- T198529; Pouch FLIR Ex and ix series
- T198533; USB cable Std A <-> Micro B
- T199362ACC; Battery Li-ion 3.6 V, 2.6 Ah, 9.4 Wh
- T198583; FLIR Tools+ (download card incl. license key)
- T199233; FLIR Atlas SDK for .NET
- T199234; FLIR Atlas SDK for MATLAB

9.7 FLIR E5 (incl. Wi-Fi)

P/N: 63909-0904
Rev.: 41166

General description
The FLIR Ex series cameras are point-and-shoot infrared cameras that give you access to the infrared world. A FLIR Ex series camera is an affordable replacement for an infrared thermometer, providing a thermal image with temperature information in every pixel. The new MSX and visual formats make the cameras incomparably easy to use.<br>The FLIR Ex series cameras are user-friendly, compact, and rugged, for use in harsh environments. The wide field of view makes them the perfect choice for building applications.
Benefits:
• Easy to use: The FLIR Ex series cameras are fully automatic and focus-free with an intuitive interface for simple measurements in thermal, visual, or MSX mode.<br>• Compact and rugged: The FLIR Ex series cameras' low weight of 0.575 kg and the accessory belt pouch make them easy to bring along at all times. Their rugged design can withstand a 2 m drop test, and ensures reliability, even in harsh environments.<br>• Ground breaking affordability: The FLIR Ex series cameras are the most affordable infrared cameras on the market.

Imaging and optical data	
IR resolution	120 × 90 pixels
Thermal sensitivity/NETD	<0.10°C (0.27°F) / <100 mK
Field of view (FOV)	45° × 34°
Minimum focus distance	0.5 m (1.6 ft.)
Spatial resolution (IFOV)	6.9 mrad
F-number	1.5
Image frequency	9 Hz
Focus	Focus free

Detector data	
Detector type	Focal plane array (FPA), uncooled microbolometer
Spectral range	7.5–13 µm

Image presentation	
Display	3.0 in. 320 × 240 color LCD
Image adjustment	Automatic adjust/lock image

Image presentation modes	
Image modes	Thermal MSX, Thermal, Picture-in-Picture, Thermal blending, Digital camera.
Multi Spectral Dynamic Imaging (MSX)	IR image with enhanced detail presentation
Picture-in-Picture	IR area on visual image

Measurement	
Object temperature range	–20°C to +250°C (–4°F to +482°F)
Accuracy	±2°C (±3.6°F) or ±2% of reading, for ambient temperature 10°C to 35°C (+50°F to 95°F) and object temperature above +0°C (+32°F)

Measurement analysis	
Spotmeter	Center spot
Area	Box with max./min.
Isotherm	Above/below/interval
Emissivity correction	Variable from 0.1 to 1.0
Emissivity table	Emissivity table of predefined materials
Reflected apparent temperature correction	Automatic, based on input of reflected temperature

Set-up	
Color palettes	Black and white, iron and rainbow
Set-up commands	Local adaptation of units, language, date and time formats

Storage of images	
File formats	Standard JPEG, 14-bit measurement data included

Digital camera	
Digital camera, resolution	640 × 480
Digital camera, FOV	55° × 43°

Data communication interfaces	
Interfaces	USB Micro: Data transfer to and from PC and Mac device
Wi-Fi	Peer-to-peer (ad hoc) or infrastructure (network)

Radio	
Wi-Fi	• Standard: 802.11 b/g/n<br>• Frequency range:<br>◦ 2400–2480 MHz<br>◦ 5150–5260 MHz<br>• Max. output power: 15 dBm

Power system	
Battery type	Rechargeable Li ion battery
Battery voltage	3.6 V
Battery operating time	Approx. 4 hours at +25°C (+77°F) ambient temperature and typical use
Charging system	Battery is charged inside the camera or in specific charger.
Charging time	2.5 hours to 90% capacity in camera. 2 hours in charger.
Power management	Automatic shut-down
AC operation	AC adapter, 90–260 VAC input, 5 VDC output to camera

Environmental data	
Operating temperature range	–15°C to +50°C (+5°F to +122°F)
Storage temperature range	–40°C to +70°C (–40°F to +158°F)
Humidity (operating and storage)	IEC 60068-2-30/24 h 95% relative humidity

**Environmental data**	
EMC	• WEEE 2012/19/EC<br>• RoHs 2011/65/EC<br>• C-Tick<br>• EN 61000-6-3<br>• EN 61000-6-2<br>• FCC 47 CFR Part 15 Class B
Radio spectrum	• Standard: 802.11 b/g/n<br>• Frequency range:<br>◦ 2400–2480 MHz<br>◦ 5150–5260 MHz<br>• Max. output power: 15 dBm
Encapsulation	IP 54 (IEC 60529)
Shock	25 g (IEC 60068-2-27)
Vibration	2 g (IEC 60068-2-6)
Drop	2 m (6.6 ft.)

**Physical data**	
Camera weight, incl. battery	0.575 kg (1.27 lb.)
Camera size (L × W × H)	244 × 95 × 140 mm (9.6 × 3.7 × 5.5 in.)
Color	Black and gray

**Certifications**	
Certification	UL, CSA, CE, PSE and CCC

**Shipping information**	
Packaging, type	Cardboard box
List of contents	• Infrared camera<br>• Hard transport case<br>• Battery (inside camera)<br>• USB cable<br>• Power supply/charger with EU, UK, US and Australian plugs<br>• Printed documentation
Packaging, weight	2.9 kg (6.4 lb.)
Packaging, size	385 × 165 × 315 mm (15.2 × 6.5 × 12.4 in.)
EAN-13	4743254002876
UPC-12	845188014124
Country of origin	Estonia

Supplies & accessories:

- T911093; Tool belt
- T198528; Hard transport case FLIR Ex-series
- T198530; Battery
- T198531; Battery charger incl power supply
- T198532; Car charger
- T198534; Power supply USB-micro
- T198529; Pouch FLIR Ex and ix series
- T198533; USB cable Std A <-> Micro B
- T199362ACC; Battery Li-ion 3.6 V, 2.6 Ah, 9.4 Wh

- T198583; FLIR Tools+ (download card incl. license key)
- T199233; FLIR Atlas SDK for .NET
- T199234; FLIR Atlas SDK for MATLAB

9.8 FLIR E6

P/N: 63902-0202
Rev.: 41166

General description
The FLIR Ex series cameras are point-and-shoot infrared cameras that give you access to the infrared world. A FLIR Ex series camera is an affordable replacement for an infrared thermometer, providing a thermal image with temperature information in every pixel. The new MSX and visual formats make the cameras incomparably easy to use.<br>The FLIR Ex series cameras are user-friendly, compact, and rugged, for use in harsh environments. The wide field of view makes them the perfect choice for building applications.
Benefits:
• Easy to use: The FLIR Ex series cameras are fully automatic and focus-free with an intuitive interface for simple measurements in thermal, visual, or MSX mode.<br>• Compact and rugged: The FLIR Ex series cameras' low weight of 0.575 kg and the accessory belt pouch make them easy to bring along at all times. Their rugged design can withstand a 2 m drop test, and ensures reliability, even in harsh environments.<br>• Ground breaking affordability: The FLIR Ex series cameras are the most affordable infrared cameras on the market.

Imaging and optical data	
IR resolution	160 × 120 pixels
Thermal sensitivity/NETD	<0.06°C (0.11°F) / <60 mK
Field of view (FOV)	45° × 34°
Minimum focus distance	0.5 m (1.6 ft.)
Spatial resolution (IFOV)	5.2 mrad
F-number	1.5
Image frequency	9 Hz
Focus	Focus free

Detector data	
Detector type	Focal plane array (FPA), uncooled microbolometer
Spectral range	7.5–13 µm

Image presentation	
Display	3.0 in. 320 × 240 color LCD
Image adjustment	Automatic/Manual

Image presentation modes	
Image modes	Thermal MSX, Thermal, Picture-in-Picture, Thermal blending, Digital camera.
Multi Spectral Dynamic Imaging (MSX)	IR image with enhanced detail presentation
Picture in Picture	IR area on visual image

Measurement	
Object temperature range	–20°C to +250°C (–4°F to +482°F)
Accuracy	±2°C (±3.6°F) or ±2% of reading, for ambient temperature 10°C to 35°C (+50°F to 95°F) and object temperature above +0°C (+32°F)

Measurement analysis	
Spotmeter	Center spot
Area	Box with max./min.
Emissivity correction	Variable from 0.1 to 1.0
Emissivity table	Emissivity table of predefined materials
Reflected apparent temperature correction	Automatic, based on input of reflected temperature

Set-up	
Color palettes	Black and white, iron and rainbow
Set-up commands	Local adaptation of units, language, date and time formats

Storage of images	
File formats	Standard JPEG, 14-bit measurement data included

Digital camera	
Digital camera, resolution	640 × 480
Digital camera, FOV	55° × 43°

Data communication interfaces	
Interfaces	USB Micro: Data transfer to and from PC and Mac device

Power system	
Battery type	Rechargeable Li ion battery
Battery voltage	3.6 V
Battery operating time	Approx. 4 hours at +25°C (+77°F) ambient temperature and typical use
Charging system	Battery is charged inside the camera or in specific charger.
Charging time	2.5 hours to 90% capacity in camera. 2 hours in charger.
Power management	Automatic shut-down
AC operation	AC adapter, 90–260 VAC input, 5 VDC output to camera

Environmental data	
Operating temperature range	–15°C to +50°C (+5°F to +122°F)
Storage temperature range	–40°C to +70°C (–40°F to +158°F)
Humidity (operating and storage)	IEC 60068-2-30/24 h 95% relative humidity
EMC	• WEEE 2012/19/EC<br>• RoHs 2011/65/EC<br>• C-Tick<br>• EN 61000-6-3<br>• EN 61000-6-2<br>• FCC 47 CFR Part 15 Class B
Encapsulation	IP 54 (IEC 60529)
Shock	25 g (IEC 60068-2-27)

Environmental data	
Vibration	2 g (IEC 60068-2-6)
Drop	2 m (6.6 ft.)

Physical data	
Camera weight, incl. battery	0.575 kg (1.27 lb.)
Camera size (L × W × H)	244 × 95 × 140 mm (9.6 × 3.7 × 5.5 in.)
Color	Black and gray

Certifications	
Certification	UL, CSA, CE, PSE and CCC

Shipping information	
Packaging, type	Cardboard box
List of contents	• Infrared camera<br>• Hard transport case<br>• Battery (inside camera)<br>• USB cable<br>• Power supply/charger with EU, UK, US and Australian plugs<br>• Printed documentation
Packaging, weight	2.9 kg (6.4 lb.)
Packaging, size	385 × 165 × 315 mm (15.2 × 6.5 × 12.4 in.)
EAN-13	4743254001008
UPC-12	845188004958
Country of origin	Estonia

Supplies & accessories:

- T911093; Tool belt
- T198528; Hard transport case FLIR Ex-series
- T198530; Battery
- T198531; Battery charger incl power supply
- T198532; Car charger
- T198534; Power supply USB-micro
- T198529; Pouch FLIR Ex and ix series
- T198533; USB cable Std A <-> Micro B
- T199362ACC; Battery Li-ion 3.6 V, 2.6 Ah, 9.4 Wh
- T198583; FLIR Tools+ (download card incl. license key)
- T199233; FLIR Atlas SDK for .NET
- T199234; FLIR Atlas SDK for MATLAB

9.9 FLIR E6 (incl. Wi-Fi)

P/N: 63907-0704
Rev.: 41166

General description
The FLIR Ex series cameras are point-and-shoot infrared cameras that give you access to the infrared world. A FLIR Ex series camera is an affordable replacement for an infrared thermometer, providing a thermal image with temperature information in every pixel. The new MSX and visual formats make the cameras incomparably easy to use.<br>The FLIR Ex series cameras are user-friendly, compact, and rugged, for use in harsh environments. The wide field of view makes them the perfect choice for building applications.
Benefits:
• Easy to use: The FLIR Ex series cameras are fully automatic and focus-free with an intuitive interface for simple measurements in thermal, visual, or MSX mode.<br>• Compact and rugged: The FLIR Ex series cameras’ low weight of 0.575 kg and the accessory belt pouch make them easy to bring along at all times. Their rugged design can withstand a 2 m drop test, and ensures reliability, even in harsh environments.<br>• Ground breaking affordability: The FLIR Ex series cameras are the most affordable infrared cameras on the market.

Imaging and optical data	
IR resolution	160 × 120 pixels
Thermal sensitivity/NETD	<0.06°C (0.11°F) / <60 mK
Field of view (FOV)	45° × 34°
Minimum focus distance	0.5 m (1.6 ft.)
Spatial resolution (IFOV)	5.2 mrad
F-number	1.5
Image frequency	9 Hz
Focus	Focus free

Detector data	
Detector type	Focal plane array (FPA), uncooled microbolometer
Spectral range	7.5–13 µm

Image presentation	
Display	3.0 in. 320 × 240 color LCD
Image adjustment	Automatic/Manual

Image presentation modes	
Image modes	Thermal MSX, Thermal, Picture-in-Picture, Thermal blending, Digital camera.
Multi Spectral Dynamic Imaging (MSX)	IR image with enhanced detail presentation
Picture-in-Picture	IR area on visual image

Measurement	
Object temperature range	–20°C to +250°C (–4°F to +482°F)
Accuracy	±2°C (±3.6°F) or ±2% of reading, for ambient temperature 10°C to 35°C (+50°F to 95°F) and object temperature above +0°C (+32°F)

Measurement analysis	
Spotmeter	Center spot
Area	Box with max./min.
Isotherm	Above/below/interval
Emissivity correction	Variable from 0.1 to 1.0
Emissivity table	Emissivity table of predefined materials
Reflected apparent temperature correction	Automatic, based on input of reflected temperature

Set-up	
Color palettes	Black and white, iron and rainbow
Set-up commands	Local adaptation of units, language, date and time formats

Storage of images	
File formats	Standard JPEG, 14-bit measurement data included

Digital camera	
Digital camera, resolution	640 × 480
Digital camera, FOV	55° × 43°

Data communication interfaces	
Interfaces	USB Micro: Data transfer to and from PC and Mac device
Wi-Fi	Peer-to-peer (ad hoc) or infrastructure (network)

Radio	
Wi-Fi	• Standard: 802.11 b/g/n<br>• Frequency range:<br>◦ 2400–2480 MHz<br>◦ 5150–5260 MHz<br>• Max. output power: 15 dBm

Power system	
Battery type	Rechargeable Li ion battery
Battery voltage	3.6 V
Battery operating time	Approx. 4 hours at +25°C (+77°F) ambient temperature and typical use
Charging system	Battery is charged inside the camera or in specific charger.
Charging time	2.5 hours to 90% capacity in camera. 2 hours in charger.
Power management	Automatic shut-down
AC operation	AC adapter, 90–260 VAC input, 5 VDC output to camera

Environmental data	
Operating temperature range	–15°C to +50°C (+5°F to +122°F)
Storage temperature range	–40°C to +70°C (–40°F to +158°F)
Humidity (operating and storage)	IEC 60068-2-30/24 h 95% relative humidity

Environmental data	
EMC	• WEEE 2012/19/EC<br>• RoHs 2011/65/EC<br>• C-Tick<br>• EN 61000-6-3<br>• EN 61000-6-2<br>• FCC 47 CFR Part 15 Class B
Radio spectrum	• ETSI EN 300 328<br>• FCC 47 CSR Part 15<br>• RSS-247 Issue 1
Encapsulation	IP 54 (IEC 60529)
Shock	25 g (IEC 60068-2-27)
Vibration	2 g (IEC 60068-2-6)
Drop	2 m (6.6 ft.)

Physical data	
Camera weight, incl. battery	0.575 kg (1.27 lb.)
Camera size (L × W × H)	244 × 95 × 140 mm (9.6 × 3.7 × 5.5 in.)
Color	Black and gray

Certifications	
Certification	UL, CSA, CE, PSE and CCC

Shipping information	
Packaging, type	Cardboard box
List of contents	• Infrared camera<br>• Hard transport case<br>• Battery (inside camera)<br>• USB cable<br>• Power supply/charger with EU, UK, US and Australian plugs<br>• Printed documentation
Packaging, weight	2.9 kg (6.4 lb.)
Packaging, size	385 × 165 × 315 mm (15.2 × 6.5 × 12.4 in.)
EAN-13	4743254002883
UPC-12	845188014131
Country of origin	Estonia

Supplies & accessories:

- T911093; Tool belt
- T198528; Hard transport case FLIR Ex-series
- T198530; Battery
- T198531; Battery charger incl power supply
- T198532; Car charger
- T198534; Power supply USB-micro
- T198529; Pouch FLIR Ex and ix series
- T198533; USB cable Std A <-> Micro B
- T199362ACC; Battery Li-ion 3.6 V, 2.6 Ah, 9.4 Wh
- T198583; FLIR Tools+ (download card incl. license key)
- T199233; FLIR Atlas SDK for .NET
- T199234; FLIR Atlas SDK for MATLAB

9.10 FLIR E8

P/N: 63903-0303
Rev.: 41166

General description
The FLIR Ex series cameras are point-and-shoot infrared cameras that give you access to the infrared world. A FLIR Ex series camera is an affordable replacement for an infrared thermometer, providing a thermal image with temperature information in every pixel. The new MSX and visual formats make the cameras incomparably easy to use.<br>The FLIR Ex series cameras are user-friendly, compact, and rugged, for use in harsh environments. The wide field of view makes them the perfect choice for building applications.
Benefits:
• Easy to use: The FLIR Ex series cameras are fully automatic and focus-free with an intuitive interface for simple measurements in thermal, visual, or MSX mode.<br>• Compact and rugged: The FLIR Ex series cameras’ low weight of 0.575 kg and the accessory belt pouch make them easy to bring along at all times. Their rugged design can withstand a 2 m drop test, and ensures reliability, even in harsh environments.<br>• Ground breaking affordability: The FLIR Ex series cameras are the most affordable infrared cameras on the market.

Imaging and optical data	
IR resolution	320 × 240 pixels
Thermal sensitivity/NETD	<0.06°C (0.11°F) / <60 mK
Field of view (FOV)	45° × 34°
Minimum focus distance	0.5 m (1.6 ft.)
Spatial resolution (IFOV)	2.6 mrad
F-number	1.5
Image frequency	9 Hz
Focus	Focus free

Detector data	
Detector type	Focal plane array (FPA), uncooled microbolometer
Spectral range	7.5–13 µm

Image presentation	
Display	3.0 in. 320 × 240 color LCD
Image adjustment	Automatic/Manual

Image presentation modes	
Image modes	Thermal MSX, Thermal, Picture-in-Picture, Thermal blending, Digital camera.
Multi Spectral Dynamic Imaging (MSX)	IR image with enhanced detail presentation
Picture in Picture	IR area on visual image

Measurement	
Object temperature range	–20°C to +250°C (–4°F to +482°F)
Accuracy	±2°C (±3.6°F) or ±2% of reading, for ambient temperature 10°C to 35°C (+50°F to 95°F) and object temperature above +0°C (+32°F)

Measurement analysis	
Spotmeter	Center spot
Area	Box with max./min.
Emissivity correction	Variable from 0.1 to 1.0
Emissivity table	Emissivity table of predefined materials
Reflected apparent temperature correction	Automatic, based on input of reflected temperature

Set-up	
Color palettes	Black and white, iron and rainbow
Set-up commands	Local adaptation of units, language, date and time formats

Storage of images	
File formats	Standard JPEG, 14-bit measurement data included

Digital camera	
Digital camera, resolution	640 × 480
Digital camera, FOV	55° × 43°

Data communication interfaces	
Interfaces	USB Micro: Data transfer to and from PC and Mac device

Power system	
Battery type	Rechargeable Li ion battery
Battery voltage	3.6 V
Battery operating time	Approx. 4 hours at +25°C (+77°F) ambient temperature and typical use
Charging system	Battery is charged inside the camera or in specific charger.
Charging time	2.5 hours to 90% capacity in camera. 2 hours in charger.
Power management	Automatic shut-down
AC operation	AC adapter, 90–260 VAC input, 5 VDC output to camera

Environmental data	
Operating temperature range	–15°C to +50°C (+5°F to +122°F)
Storage temperature range	–40°C to +70°C (–40°F to +158°F)
Humidity (operating and storage)	IEC 60068-2-30/24 h 95% relative humidity
EMC	• WEEE 2012/19/EC<br>• RoHs 2011/65/EC<br>• C-Tick<br>• EN 61000-6-3<br>• EN 61000-6-2<br>• FCC 47 CFR Part 15 Class B
Encapsulation	IP 54 (IEC 60529)
Shock	25 g (IEC 60068-2-27)

Environmental data	
Vibration	2 g (IEC 60068-2-6)
Drop	2 m (6.6 ft.)

Physical data	
Camera weight, incl. battery	0.575 kg (1.27 lb.)
Camera size (L × W × H)	244 × 95 × 140 mm (9.6 × 3.7 × 5.5 in.)
Color	Black and gray

Certifications	
Certification	UL, CSA, CE, PSE and CCC

Shipping information	
Packaging, type	Cardboard box
List of contents	• Infrared camera<br>• Hard transport case<br>• Battery (2x)<br>• USB cable<br>• Power supply/charger with EU, UK, US and Australian plugs<br>• Battery charger<br>• Printed documentation
Packaging, weight	3.13 kg (6.9 lb.)
Packaging, size	385 × 165 × 315 mm (15.2 × 6.5 × 12.4 in.)
EAN-13	4743254001015
UPC-12	845188004965
Country of origin	Estonia

Supplies & accessories:

- T911093; Tool belt
- T198528; Hard transport case FLIR Ex-series
- T198530; Battery
- T198531; Battery charger incl power supply
- T198532; Car charger
- T198534; Power supply USB-micro
- T198529; Pouch FLIR Ex and ix series
- T198533; USB cable Std A <-> Micro B
- T199362ACC; Battery Li-ion 3.6 V, 2.6 Ah, 9.4 Wh
- T198583; FLIR Tools+ (download card incl. license key)
- T199233; FLIR Atlas SDK for .NET
- T199234; FLIR Atlas SDK for MATLAB

9.11 FLIR E8 (incl. Wi-Fi)

P/N: 63908-0805
Rev.: 41166

General description
The FLIR Ex series cameras are point-and-shoot infrared cameras that give you access to the infrared world. A FLIR Ex series camera is an affordable replacement for an infrared thermometer, providing a thermal image with temperature information in every pixel. The new MSX and visual formats make the cameras incomparably easy to use.<br>The FLIR Ex series cameras are user-friendly, compact, and rugged, for use in harsh environments. The wide field of view makes them the perfect choice for building applications.
Benefits:
• Easy to use: The FLIR Ex series cameras are fully automatic and focus-free with an intuitive interface for simple measurements in thermal, visual, or MSX mode.<br>• Compact and rugged: The FLIR Ex series cameras' low weight of 0.575 kg and the accessory belt pouch make them easy to bring along at all times. Their rugged design can withstand a 2 m drop test, and ensures reliability, even in harsh environments.<br>• Ground breaking affordability: The FLIR Ex series cameras are the most affordable infrared cameras on the market.

Imaging and optical data	
IR resolution	320 × 240 pixels
Thermal sensitivity/NETD	<0.06°C (0.11°F) / <60 mK
Field of view (FOV)	45° × 34°
Minimum focus distance	0.5 m (1.6 ft.)
Spatial resolution (IFOV)	2.6 mrad
F-number	1.5
Image frequency	9 Hz
Focus	Focus free

Detector data	
Detector type	Focal plane array (FPA), uncooled microbolometer
Spectral range	7.5–13 µm

Image presentation	
Display	3.0 in. 320 × 240 color LCD
Image adjustment	Automatic/Manual

Image presentation modes	
Image modes	Thermal MSX, Thermal, Picture-in-Picture, Thermal blending, Digital camera.
Multi Spectral Dynamic Imaging (MSX)	IR image with enhanced detail presentation
Picture-in-Picture	IR area on visual image

Measurement	
Object temperature range	–20°C to +250°C (–4°F to +482°F)
Accuracy	±2°C (±3.6°F) or ±2% of reading, for ambient temperature 10°C to 35°C (+50°F to 95°F) and object temperature above +0°C (+32°F)

Measurement analysis	
Spotmeter	Center spot
Area	Box with max./min.
Isotherm	Above/below/interval
Emissivity correction	Variable from 0.1 to 1.0
Emissivity table	Emissivity table of predefined materials
Reflected apparent temperature correction	Automatic, based on input of reflected temperature

Set-up	
Color palettes	Black and white, iron and rainbow
Set-up commands	Local adaptation of units, language, date and time formats

Storage of images	
File formats	Standard JPEG, 14-bit measurement data included

Digital camera	
Digital camera, resolution	640 × 480
Digital camera, FOV	55° × 43°

Data communication interfaces	
Interfaces	USB Micro: Data transfer to and from PC and Mac device
Wi-Fi	Peer-to-peer (ad hoc) or infrastructure (network)

Radio	
Wi-Fi	• Standard: 802.11 b/g/n<br>• Frequency range:<br>◦ 2400–2480 MHz<br>◦ 5150–5260 MHz<br>• Max. output power: 15 dBm

Power system	
Battery type	Rechargeable Li ion battery
Battery voltage	3.6 V
Battery operating time	Approx. 4 hours at +25°C (+77°F) ambient temperature and typical use
Charging system	Battery is charged inside the camera or in specific charger.
Charging time	2.5 hours to 90% capacity in camera. 2 hours in charger.
Power management	Automatic shut-down
AC operation	AC adapter, 90–260 VAC input, 5 VDC output to camera

Environmental data	
Operating temperature range	–15°C to +50°C (+5°F to +122°F)
Storage temperature range	–40°C to +70°C (–40°F to +158°F)
Humidity (operating and storage)	IEC 60068-2-30/24 h 95% relative humidity

Environmental data	
EMC	• WEEE 2012/19/EC<br>• RoHs 2011/65/EC<br>• C-Tick<br>• EN 61000-6-3<br>• EN 61000-6-2<br>• FCC 47 CFR Part 15 Class B
Radio spectrum	• ETSI EN 300 328<br>• FCC 47 CSR Part 15<br>• RSS-247 Issue 1
Encapsulation	IP 54 (IEC 60529)
Shock	25 g (IEC 60068-2-27)
Vibration	2 g (IEC 60068-2-6)
Drop	2 m (6.6 ft.)

Physical data	
Camera weight, incl. battery	0.575 kg (1.27 lb.)
Camera size (L × W × H)	244 × 95 × 140 mm (9.6 × 3.7 × 5.5 in.)
Color	Black and gray

Certifications	
Certification	UL, CSA, CE, PSE and CCC

Shipping information	
Packaging, type	Cardboard box
List of contents	• Infrared camera<br>• Hard transport case<br>• Battery (2x)<br>• USB cable<br>• Power supply/charger with EU, UK, US and Australian plugs<br>• Battery charger<br>• Printed documentation
Packaging, weight	3.13 kg (6.9 lb.)
Packaging, size	385 × 165 × 315 mm (15.2 × 6.5 × 12.4 in.)
EAN-13	4743254002890
UPC-12	845188014148
Country of origin	Estonia

Supplies & accessories:

- T911093; Tool belt
- T198528; Hard transport case FLIR Ex-series
- T198530; Battery
- T198531; Battery charger incl power supply
- T198532; Car charger
- T198534; Power supply USB-micro
- T198529; Pouch FLIR Ex and ix series
- T198533; USB cable Std A <-> Micro B
- T199362ACC; Battery Li-ion 3.6 V, 2.6 Ah, 9.4 Wh
- T198583; FLIR Tools+ (download card incl. license key)
- T199233; FLIR Atlas SDK for .NET

- T199234; FLIR Atlas SDK for MATLAB

10 Mechanical drawings

[See next page]

Camera with built-in IR lens f=6,5 mm (45°)

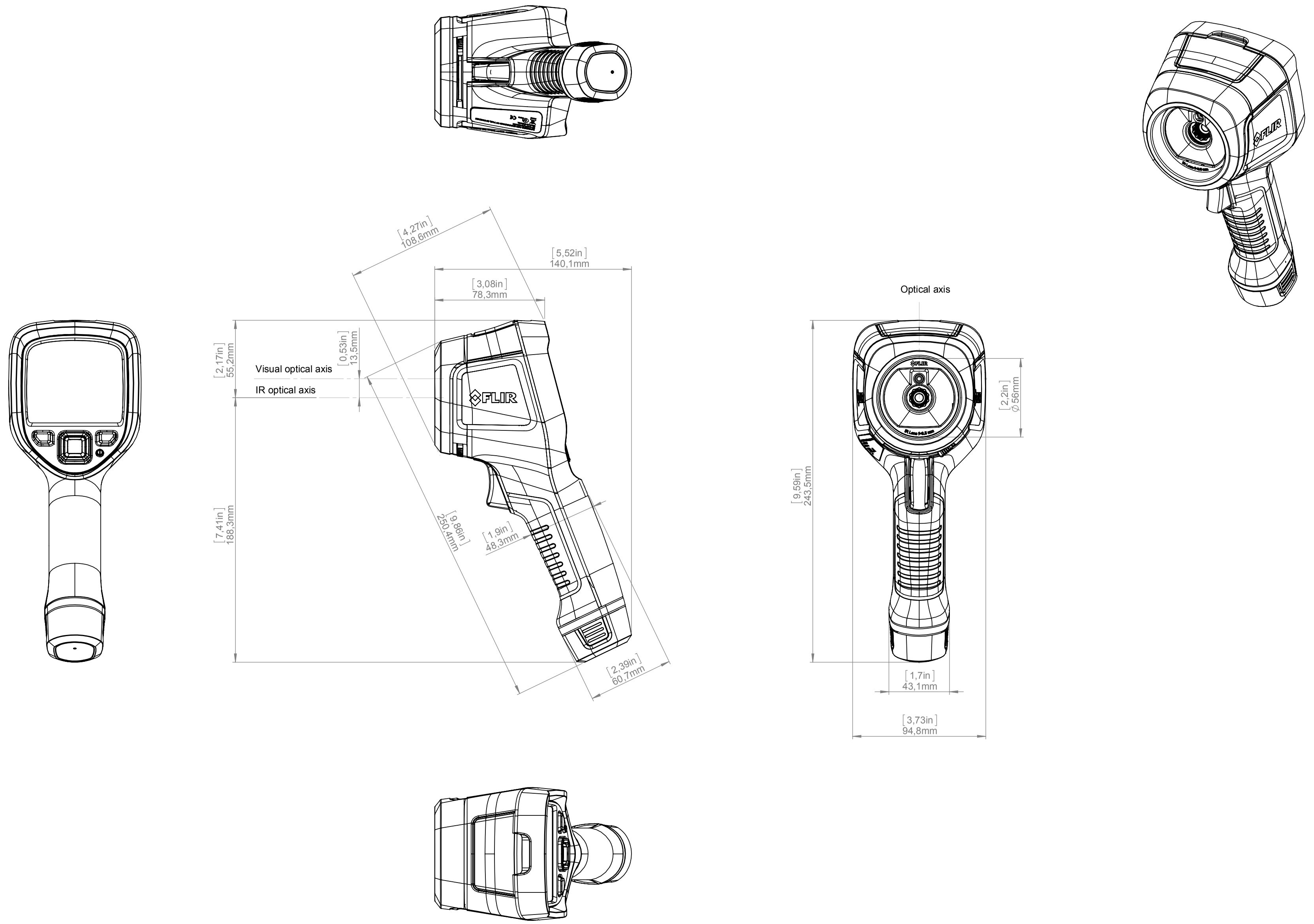

Modified	Check	Drawn by
2013-03-25	CAHA	R&D Thermography

Denomination: Basic dimensions FLIR Ex

Size: A2 | Scale: 1:2 | Sheet: 1(2) | Drawing No. T127831 | Size: A

© 2012, FLIR Systems, Inc. All rights reserved worldwide. No part of this drawing may be reproduced, stored in a retrieval system, or transmitted in any form, or by any means, electronic, mechanical, photocopying, recording, or otherwise, without written permission from FLIR Systems, Inc. Specifications subject to change without further notice. Dimensional data is based on nominal values. Products may be subject to regional market considerations. License procedures may apply. Product may be subject to US Export Regulations. Please refer to exportquestions@flir.com with any questions. Diversion contrary to US law is prohibited.

Charger and Power pack

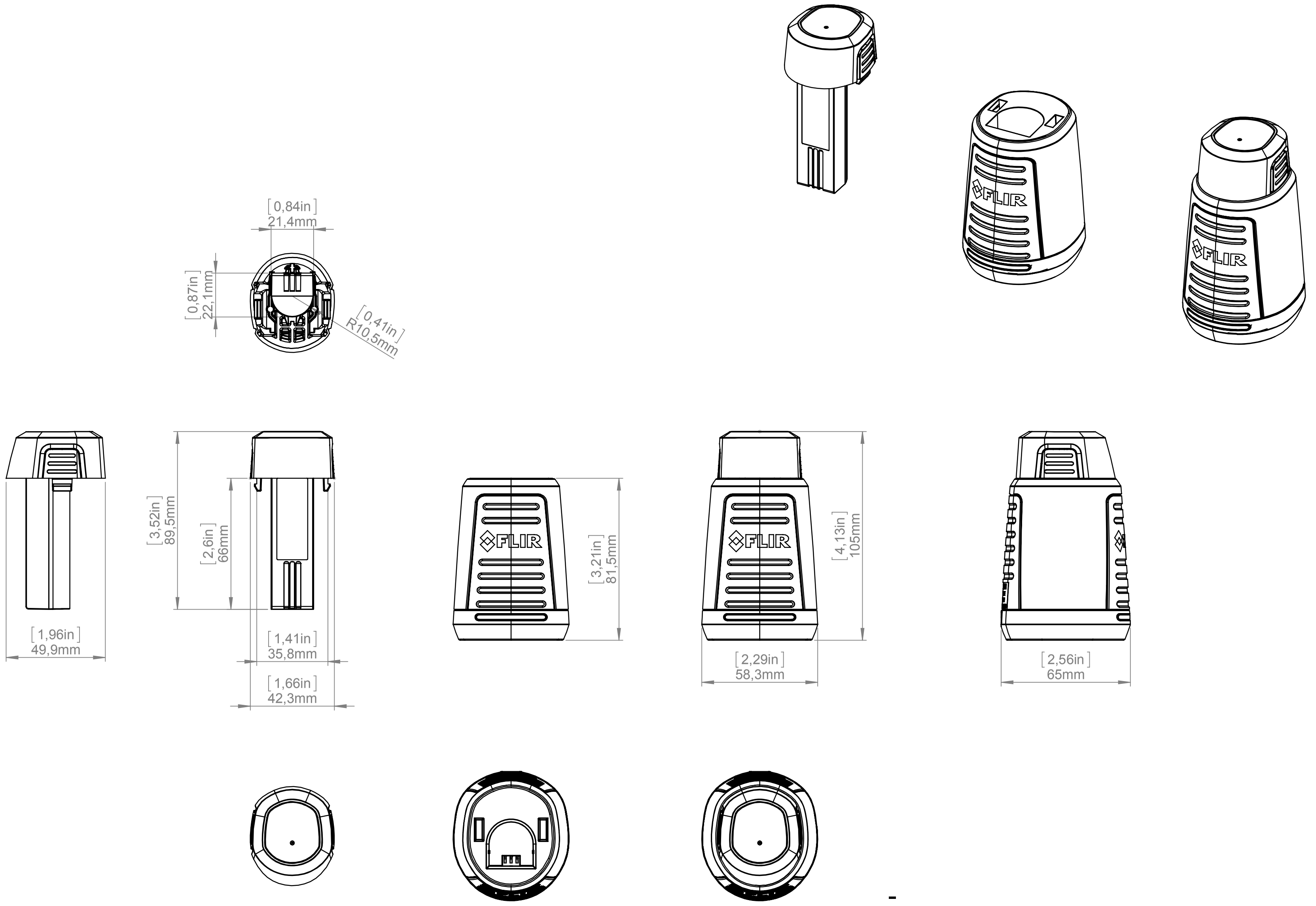

Modified	Check	Drawn by
2013-03-25	CAHA	R&D Thermography

Denomination: Basic dimensions FLIR Ex

Size: A3
Scale: 1:2
Sheet: 2(2)
Drawing No.: T127831
Size: A

© 2012, FLIR Systems, Inc. All rights reserved worldwide. No part of this drawing may be reproduced, stored in a retrieval system, or transmitted in any form, or by any means, electronic, mechanical, photocopying, recording, or otherwise, without written permission from FLIR Systems, Inc. Specifications subject to change without further notice. Dimensional data is based on nominal values. Products may be subject to regional market considerations. License procedures may apply. Product may be subject to US Export Regulations. Please refer to exportquestions@flir.com with any questions. Diversion contrary to US law is prohibited.

11 CE Declaration of conformity

[See next page]

FLIR

August 8, 2013 AQ320035

CE Declaration of Conformity

This is to certify that the Systems listed below have been designed and manufactured to meet the requirements, as applicable, of the following EU-Directives and corresponding harmonising standards. The systems consequently meet the requirements for the CE-mark.

Directives:

**Directive 2004/108/EC;**	**Electromagnetic Compatibility**
**Directive 2006/95/EC;**	**"Low voltage Directive"** (Power Supply)

Standards:

**Emission:**	**EN 61000-6-3;**	**Electro magnetic Compatibility**<br>**Generic standards - Emission**
**Immunity:**	**EN 61000-6-2;**	**Electro magnetic Compatibility;**<br>**Generic standards - Immunity**
**Safety** (Power Supply):	**EN 60950**	(or other)<br>**Safety of information technology equipment**

Systems: **FLIR EX**

FLIR Systems AB
Quality Assurance

Björn Svensson
Director

12 Cleaning the camera

12.1 Camera housing, cables, and other items

12.1.1 Liquids

Use one of these liquids:

- Warm water
- A weak detergent solution

12.1.2 Equipment

A soft cloth

12.1.3 Procedure

Follow this procedure:

1. Soak the cloth in the liquid.
2. Twist the cloth to remove excess liquid.
3. Clean the part with the cloth.

> ⚠ **CAUTION**
>
> Do not apply solvents or similar liquids to the camera, the cables, or other items. This can cause damage.

12.2 Infrared lens

12.2.1 Liquids

Use one of these liquids:

- A commercial lens cleaning liquid with more than 30% isopropyl alcohol.
- 96% ethyl alcohol (C_2H_5OH).

12.2.2 Equipment

Cotton wool

> ⚠ **CAUTION**
>
> If you use a lens cleaning cloth it must be dry. Do not use a lens cleaning cloth with the liquids that are given in section 12.2.1 above. These liquids can cause material on the lens cleaning cloth to become loose. This material can have an unwanted effect on the surface of the lens.

12.2.3 Procedure

Follow this procedure:

1. Soak the cotton wool in the liquid.
2. Twist the cotton wool to remove excess liquid.
3. Clean the lens one time only and discard the cotton wool.

> ⚠ **WARNING**
>
> Make sure that you read all applicable MSDS (Material Safety Data Sheets) and warning labels on containers before you use a liquid: the liquids can be dangerous.

12 Cleaning the camera

> ⚠ **CAUTION**
>
> - Be careful when you clean the infrared lens. The lens has a delicate anti-reflective coating.
> - Do not clean the infrared lens too vigorously. This can damage the anti-reflective coating.

13 Application examples

13.1 Moisture & water damage

13.1.1 General

It is often possible to detect moisture and water damage in a house by using an infrared camera. This is partly because the damaged area has a different heat conduction property and partly because it has a different thermal capacity to store heat than the surrounding material.

Many factors can come into play as to how moisture or water damage will appear in an infrared image.

For example, heating and cooling of these parts takes place at different rates depending on the material and the time of day. For this reason, it is important that other methods are used as well to check for moisture or water damage.

13.1.2 Figure

The image below shows extensive water damage on an external wall where the water has penetrated the outer facing because of an incorrectly installed window ledge.

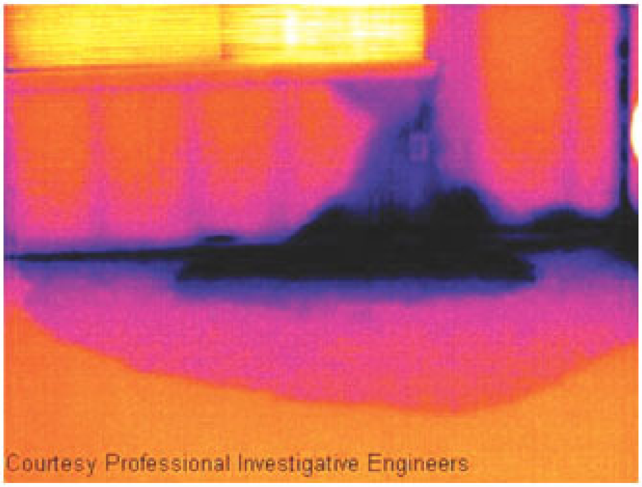

13.2 Faulty contact in socket

13.2.1 General

Depending on the type of connection a socket has, an improperly connected wire can result in local temperature increase. This temperature increase is caused by the reduced contact area between the connection point of the incoming wire and the socket , and can result in an electrical fire.

A socket's construction may differ dramatically from one manufacturer to another. For this reason, different faults in a socket can lead to the same typical appearance in an infrared image.

Local temperature increase can also result from improper contact between wire and socket, or from difference in load.

13.2.2 Figure

The image below shows a connection of a cable to a socket where improper contact in the connection has resulted in local temperature increase.

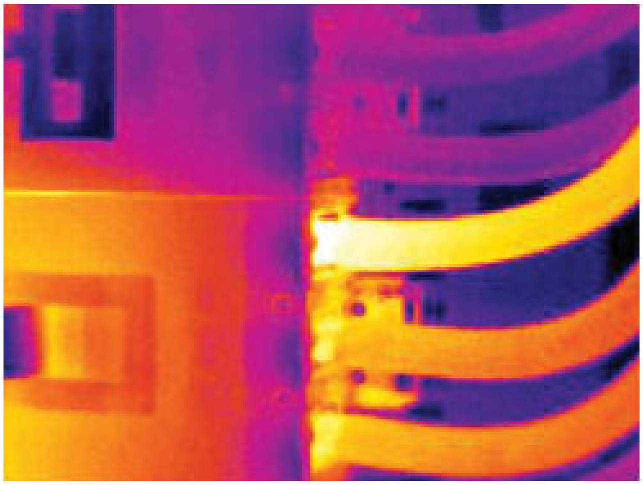

13.3 Oxidized socket

13.3.1 General

Depending on the type of socket and the environment in which the socket is installed, oxides may occur on the socket's contact surfaces. These oxides can lead to locally increased resistance when the socket is loaded, which can be seen in an infrared image as local temperature increase.

A socket’s construction may differ dramatically from one manufacturer to another. For this reason, different faults in a socket can lead to the same typical appearance in an infrared image.

Local temperature increase can also result from improper contact between a wire and socket, or from difference in load.

13.3.2 Figure

The image below shows a series of fuses where one fuse has a raised temperature on the contact surfaces against the fuse holder. Because of the fuse holder’s blank metal, the temperature increase is not visible there, while it is visible on the fuse’s ceramic material.

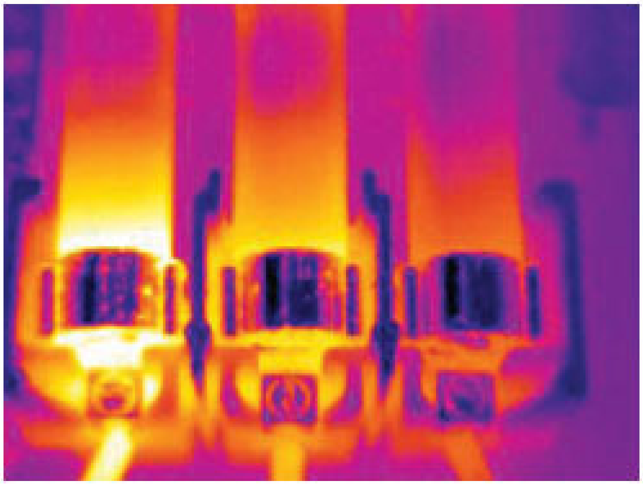

13.4 Insulation deficiencies

13.4.1 General

Insulation deficiencies may result from insulation losing volume over the course of time and thereby not entirely filling the cavity in a frame wall.

An infrared camera allows you to see these insulation deficiencies because they either have a different heat conduction property than sections with correctly installed insulation, and/or show the area where air is penetrating the frame of the building.

When you are inspecting a building, the temperature difference between the inside and outside should be at least 10°C (18°F). Studs, water pipes, concrete columns, and similar components may resemble an insulation deficiency in an infrared image. Minor differences may also occur naturally.

13.4.2 Figure

In the image below, insulation in the roof framing is lacking. Due to the absence of insulation, air has forced its way into the roof structure, which thus takes on a different characteristic appearance in the infrared image.

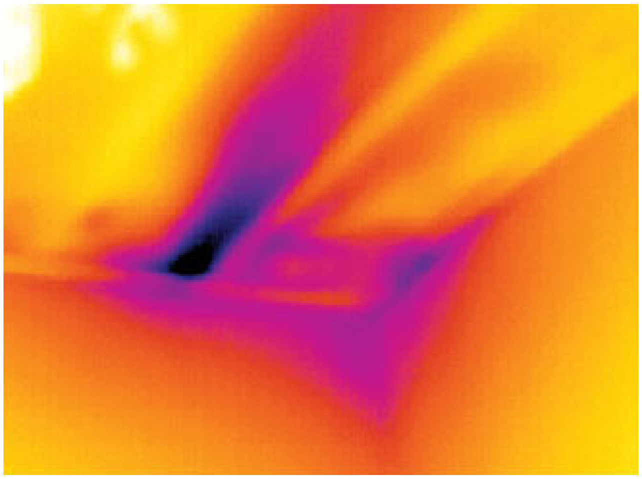

13.5 Draft

13.5.1 General

Draft can be found under baseboards, around door and window casings, and above ceiling trim. This type of draft is often possible to see with an infrared camera, as a cooler airstream cools down the surrounding surface.

When you are investigating draft in a house, there should be sub-atmospheric pressure in the house. Close all doors, windows, and ventilation ducts, and allow the kitchen fan to run for a while before you take the infrared images.

An infrared image of draft often shows a typical stream pattern. You can see this stream pattern clearly in the picture below.

Also keep in mind that drafts can be concealed by heat from floor heating circuits.

13.5.2 Figure

The image below shows a ceiling hatch where faulty installation has resulted in a strong draft.

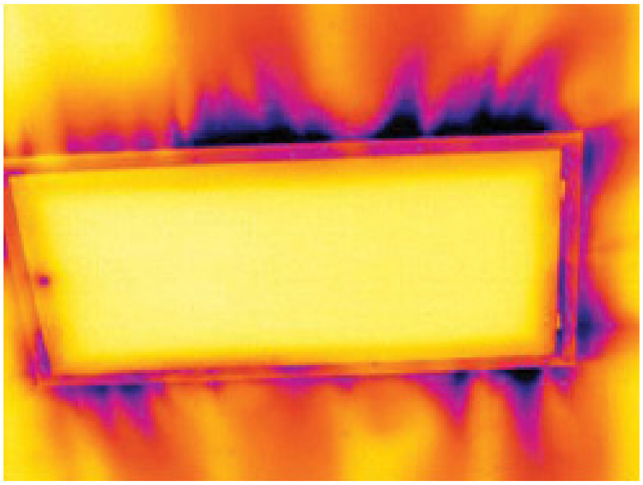

14 About FLIR Systems

FLIR Systems was established in 1978 to pioneer the development of high-performance infrared imaging systems, and is the world leader in the design, manufacture, and marketing of thermal imaging systems for a wide variety of commercial, industrial, and government applications. Today, FLIR Systems embraces five major companies with outstanding achievements in infrared technology since 1958—the Swedish AGEMA Infrared Systems (formerly AGA Infrared Systems), the three United States companies Indigo Systems, FSI, and Inframetrics, and the French company Cedip.

Since 2007, FLIR Systems has acquired several companies with world-leading expertise in sensor technologies:

- Extech Instruments (2007)
- Ifara Tecnologías (2008)
- Salvador Imaging (2009)
- OmniTech Partners (2009)
- Directed Perception (2009)
- Raymarine (2010)
- ICx Technologies (2010)
- TackTick Marine Digital Instruments (2011)
- Aerius Photonics (2011)
- Lorex Technology (2012)
- Traficon (2012)
- MARSS (2013)
- DigitalOptics micro-optics business (2013)
- DVTEL (2015)
- Point Grey Research (2016)
- Prox Dynamics (2016)

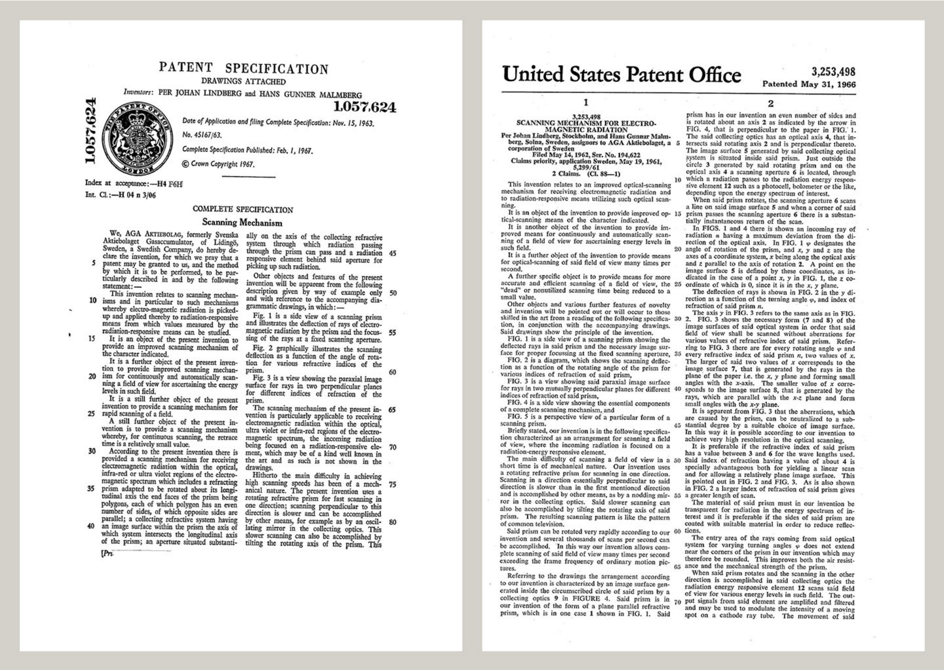
PATENT SPECIFICATION

DRAWINGS ATTACHED

1.057.624

COMPLETE SPECIFICATION

Scanning Mechanism

United States Patent Office

3,253,498

Patented May 31, 1966

SCANNING MECHANISM FOR ELECTRO-MAGNETIC RADIATION

Figure 14.1 Patent documents from the early 1960s

FLIR Systems has three manufacturing plants in the United States (Portland, OR, Boston, MA, Santa Barbara, CA) and one in Sweden (Stockholm). Since 2007 there is also a

manufacturing plant in Tallinn, Estonia. Direct sales offices in Belgium, Brazil, China, France, Germany, Great Britain, Hong Kong, Italy, Japan, Korea, Sweden, and the USA—together with a worldwide network of agents and distributors—support our international customer base.

FLIR Systems is at the forefront of innovation in the infrared camera industry. We anticipate market demand by constantly improving our existing cameras and developing new ones. The company has set milestones in product design and development such as the introduction of the first battery-operated portable camera for industrial inspections, and the first uncooled infrared camera, to mention just two innovations.

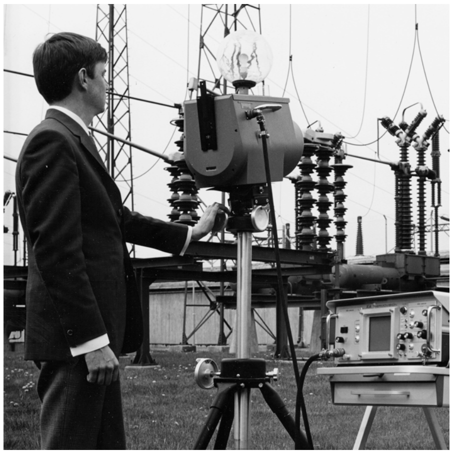

Figure 14.2 1969: Thermovision Model 661. The camera weighed approximately 25 kg (55 lb.), the oscilloscope 20 kg (44 lb.), and the tripod 15 kg (33 lb.). The operator also needed a 220 VAC generator set, and a 10 L (2.6 US gallon) jar with liquid nitrogen. To the left of the oscilloscope the Polaroid attachment (6 kg/13 lb.) can be seen.

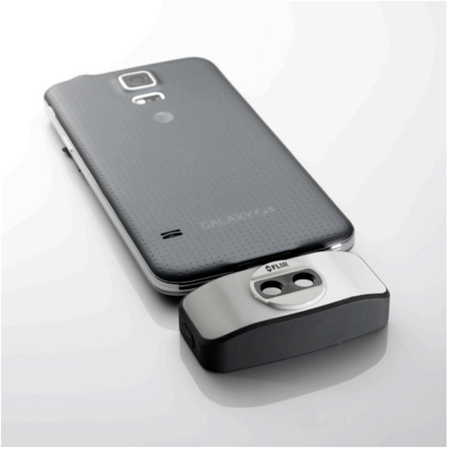

Figure 14.3 2015: FLIR One, an accessory to iPhone and Android mobile phones. Weight: 90 g (3.2 oz.).

FLIR Systems manufactures all vital mechanical and electronic components of the camera systems itself. From detector design and manufacturing, to lenses and system electronics, to final testing and calibration, all production steps are carried out and supervised by our own engineers. The in-depth expertise of these infrared specialists ensures the accuracy and reliability of all vital components that are assembled into your infrared camera.

14.1 More than just an infrared camera

At FLIR Systems we recognize that our job is to go beyond just producing the best infrared camera systems. We are committed to enabling all users of our infrared camera systems to work more productively by providing them with the most powerful camera–software combination. Especially tailored software for predictive maintenance, R & D, and process monitoring is developed in-house. Most software is available in a wide variety of languages.

We support all our infrared cameras with a wide variety of accessories to adapt your equipment to the most demanding infrared applications.

14.2 Sharing our knowledge

Although our cameras are designed to be very user-friendly, there is a lot more to thermography than just knowing how to handle a camera. Therefore, FLIR Systems has founded the Infrared Training Center (ITC), a separate business unit, that provides certified training courses. Attending one of the ITC courses will give you a truly hands-on learning experience.

The staff of the ITC are also there to provide you with any application support you may need in putting infrared theory into practice.

14.3 Supporting our customers

FLIR Systems operates a worldwide service network to keep your camera running at all times. If you discover a problem with your camera, local service centers have all the equipment and expertise to solve it within the shortest possible time. Therefore, there is no need to send your camera to the other side of the world or to talk to someone who does not speak your language.

15 Terms, laws, and definitions

Term	Definition
Absorption and emission[2]	The capacity or ability of an object to absorb incident radiated energy is always the same as the capacity to emit its own energy as radiation
Apparent temperature	uncompensated reading from an infrared instrument, containing all radiation incident on the instrument, regardless of its sources[3]
Color palette	assigns different colors to indicate specific levels of apparent temperature. Palettes can provide high or low contrast, depending on the colors used in them
Conduction	direct transfer of thermal energy from molecule to molecule, caused by collisions between the molecules
Convection	heat transfer mode where a fluid is brought into motion, either by gravity or another force, thereby transferring heat from one place to another
Diagnostics	examination of symptoms and syndromes to determine the nature of faults or failures[4]
Direction of heat transfer[5]	Heat will spontaneously flow from hotter to colder, thereby transferring thermal energy from one place to another[6]
Emissivity	ratio of the power radiated by real bodies to the power that is radiated by a blackbody at the same temperature and at the same wavelength[7]
Energy conservation[8]	The sum of the total energy contents in a closed system is constant
Exitant radiation	radiation that leaves the surface of an object, regardless of its original sources
Heat	thermal energy that is transferred between two objects (systems) due to their difference in temperature
Heat transfer rate[9]	The heat transfer rate under steady state conditions is directly proportional to the thermal conductivity of the object, the cross-sectional area of the object through which the heat flows, and the temperature difference between the two ends of the object. It is inversely proportional to the length, or thickness, of the object[10]
Incident radiation	radiation that strikes an object from its surroundings
IR thermography	process of acquisition and analysis of thermal information from non-contact thermal imaging devices
Isotherm	replaces certain colors in the scale with a contrasting color. It marks an interval of equal apparent temperature[11]

2. Kirchhoff's law of thermal radiation.
3. Based on ISO 18434-1:2008 (en).
4. Based on ISO 13372:2004 (en).
5. 2nd law of thermodynamics.
6. This is a consequence of the 2nd law of thermodynamics, the law itself is more complicated.
7. Based on ISO 16714-3:2016 (en).
8. 1st law of thermodynamics.
9. Fourier's law.
10. This is the one-dimensional form of Fourier's law, valid for steady-state conditions.
11. Based on ISO 18434-1:2008 (en)

Term	Definition
Qualitative thermography	thermography that relies on the analysis of thermal patterns to reveal the existence of and to locate the position of anomalies[12]
Quantitative thermography	thermography that uses temperature measurement to determine the seriousness of an anomaly, in order to establish repair priorities[12]
Radiative heat transfer	Heat transfer by the emission and absorption of thermal radiation
Reflected apparent temperature	apparent temperature of the environment that is reflected by the target into the IR camera[13]
Spatial resolution	ability of an IR camera to resolve small objects or details
Temperature	measure of the average kinetic energy of the molecules and atoms that make up the substance
Thermal energy	total kinetic energy of the molecules that make up the object[14]
Thermal gradient	gradual change in temperature over distance[13]
Thermal tuning	process of putting the colors of the image on the object of analysis, in order to maximize contrast

12. Based on ISO 10878-2013 (en).
13. Based on ISO 16714-3:2016 (en).
14. Thermal energy is part of the internal energy of an object.

16 Thermographic measurement techniques

16.1 Introduction

An infrared camera measures and images the emitted infrared radiation from an object. The fact that radiation is a function of object surface temperature makes it possible for the camera to calculate and display this temperature.

However, the radiation measured by the camera does not only depend on the temperature of the object but is also a function of the emissivity. Radiation also originates from the surroundings and is reflected in the object. The radiation from the object and the reflected radiation will also be influenced by the absorption of the atmosphere.

To measure temperature accurately, it is therefore necessary to compensate for the effects of a number of different radiation sources. This is done on-line automatically by the camera. The following object parameters must, however, be supplied for the camera:

- The emissivity of the object
- The reflected apparent temperature
- The distance between the object and the camera
- The relative humidity
- Temperature of the atmosphere

16.2 Emissivity

The most important object parameter to set correctly is the emissivity which, in short, is a measure of how much radiation is emitted from the object, compared to that from a perfect blackbody of the same temperature.

Normally, object materials and surface treatments exhibit emissivity ranging from approximately 0.1 to 0.95. A highly polished (mirror) surface falls below 0.1, while an oxidized or painted surface has a higher emissivity. Oil-based paint, regardless of color in the visible spectrum, has an emissivity over 0.9 in the infrared. Human skin exhibits an emissivity 0.97 to 0.98.

Non-oxidized metals represent an extreme case of perfect opacity and high reflexivity, which does not vary greatly with wavelength. Consequently, the emissivity of metals is low – only increasing with temperature. For non-metals, emissivity tends to be high, and decreases with temperature.

16.2.1 Finding the emissivity of a sample

16.2.1.1 Step 1: Determining reflected apparent temperature

Use one of the following two methods to determine reflected apparent temperature:

16.2.1.1.1 Method 1: Direct method

Follow this procedure:

1. Look for possible reflection sources, considering that the incident angle = reflection angle (a = b).

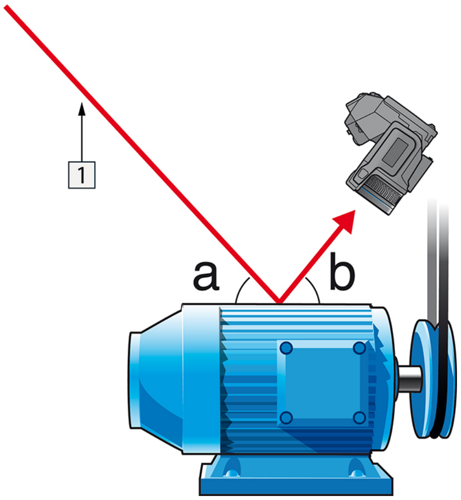

Figure 16.1 1 = Reflection source

2. If the reflection source is a spot source, modify the source by obstructing it using a piece if cardboard.

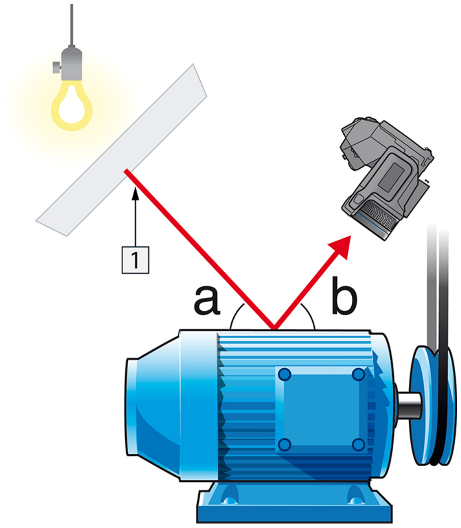

Figure 16.2 1 = Reflection source

3. Measure the radiation intensity (= apparent temperature) from the reflection source using the following settings:
   - Emissivity: 1.0
   - D_{obj}: 0

   You can measure the radiation intensity using one of the following two methods:

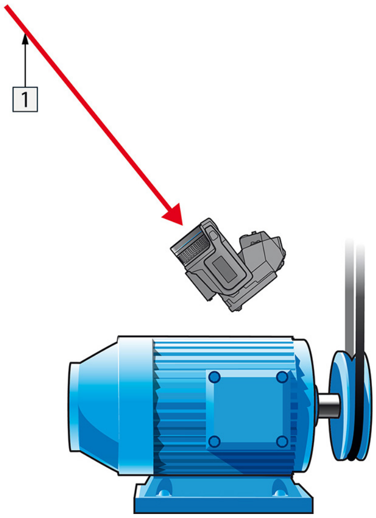

Figure 16.3 1 = Reflection source

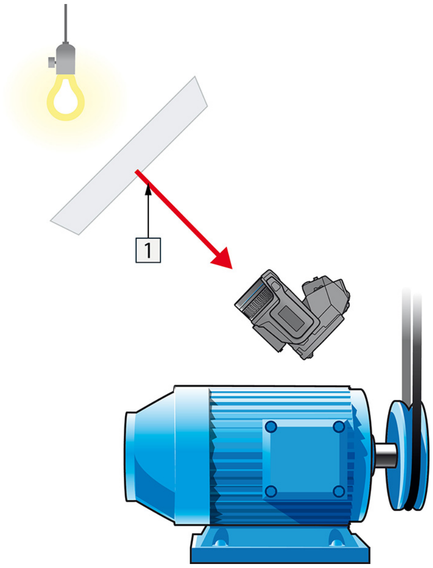

Figure 16.4 1 = Reflection source

You can not use a thermocouple to measure reflected apparent temperature, because a thermocouple measures *temperature*, but apparent temperatrure is *radiation intensity*.

16.2.1.1.2 Method 2: Reflector method

Follow this procedure:

1. Crumble up a large piece of aluminum foil.
2. Uncrumble the aluminum foil and attach it to a piece of cardboard of the same size.
3. Put the piece of cardboard in front of the object you want to measure. Make sure that the side with aluminum foil points to the camera.
4. Set the emissivity to 1.0.

5. Measure the apparent temperature of the aluminum foil and write it down. The foil is considered a perfect reflector, so its apparent temperature equals the reflected apparent temperature from the surroundings.

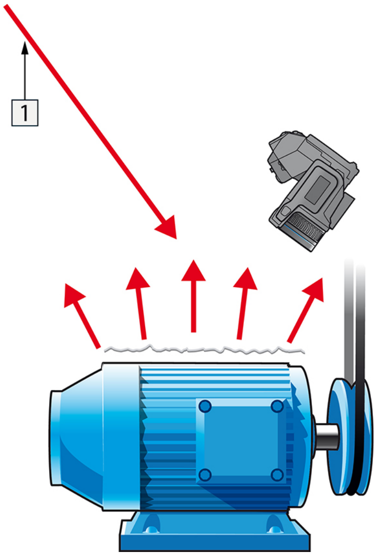

Figure 16.5 Measuring the apparent temperature of the aluminum foil.

16.2.1.2 Step 2: Determining the emissivity

Follow this procedure:

1. Select a place to put the sample.
2. Determine and set reflected apparent temperature according to the previous procedure.
3. Put a piece of electrical tape with known high emissivity on the sample.
4. Heat the sample at least 20 K above room temperature. Heating must be reasonably even.
5. Focus and auto-adjust the camera, and freeze the image.
6. Adjust *Level* and *Span* for best image brightness and contrast.
7. Set emissivity to that of the tape (usually 0.97).
8. Measure the temperature of the tape using one of the following measurement functions:
   - *Isotherm* (helps you to determine both the temperature and how evenly you have heated the sample)
   - *Spot* (simpler)
   - *Box Avg* (good for surfaces with varying emissivity).
9. Write down the temperature.
10. Move your measurement function to the sample surface.
11. Change the emissivity setting until you read the same temperature as your previous measurement.
12. Write down the emissivity.

Note

- Avoid forced convection
- Look for a thermally stable surrounding that will not generate spot reflections
- Use high quality tape that you know is not transparent, and has a high emissivity you are certain of
- This method assumes that the temperature of your tape and the sample surface are the same. If they are not, your emissivity measurement will be wrong.

16.3 Reflected apparent temperature

This parameter is used to compensate for the radiation reflected in the object. If the emissivity is low and the object temperature relatively far from that of the reflected it will be important to set and compensate for the reflected apparent temperature correctly.

16.4 Distance

The distance is the distance between the object and the front lens of the camera. This parameter is used to compensate for the following two facts:

- That radiation from the target is absorbed by the atmosphere between the object and the camera.
- That radiation from the atmosphere itself is detected by the camera.

16.5 Relative humidity

The camera can also compensate for the fact that the transmittance is also dependent on the relative humidity of the atmosphere. To do this set the relative humidity to the correct value. For short distances and normal humidity the relative humidity can normally be left at a default value of 50%.

16.6 Other parameters

In addition, some cameras and analysis programs from FLIR Systems allow you to compensate for the following parameters:

- Atmospheric temperature – *i.e.* the temperature of the atmosphere between the camera and the target
- External optics temperature – *i.e.* the temperature of any external lenses or windows used in front of the camera
- External optics transmittance – *i.e.* the transmission of any external lenses or windows used in front of the camera

17 About calibration

17.1 Introduction

Calibration of a thermal camera is a prerequisite for temperature measurement. The calibration provides the relationship between the input signal and the physical quantity that the user wants to measure. However, despite its widespread and frequent use, the term “calibration” is often misunderstood and misused. Local and national differences as well as translation-related issues create additional confusion.

Unclear terminology can lead to difficulties in communication and erroneous translations, and subsequently to incorrect measurements due to misunderstandings and, in the worst case, even to lawsuits.

17.2 Definition—what is calibration?

The International Bureau of Weights and Measures[15] defines *calibration*[16] in the following way:

an operation that, under specified conditions, in a first step, establishes a relation between the quantity values with measurement uncertainties provided by measurement standards and corresponding indications with associated measurement uncertainties and, in a second step, uses this information to establish a relation for obtaining a measurement result from an indication.

The calibration itself may be expressed in different formats: this can be a statement, calibration function, calibration diagram[17], calibration curve[18], or calibration table.

Often, the first step alone in the above definition is perceived and referred to as being “calibration.” However, this is not (always) sufficient.

Considering the calibration procedure of a thermal camera, the first step establishes the relation between emitted radiation (the quantity value) and the electrical output signal (the indication). This first step of the calibration procedure consists of obtaining a homogeneous (or uniform) response when the camera is placed in front of an extended source of radiation.

As we know the temperature of the reference source emitting the radiation, in the second step the obtained output signal (the indication) can be related to the reference source’s temperature (measurement result). The second step includes drift measurement and compensation.

To be correct, calibration of a thermal camera is, strictly, not expressed through temperature. Thermal cameras are sensitive to infrared radiation: therefore, at first you obtain a radiance correspondence, then a relationship between radiance and temperature. For bolometer cameras used by non-R&D customers, radiance is not expressed: only the temperature is provided.

17.3 Camera calibration at FLIR Systems

Without calibration, an infrared camera would not be able to measure either radiance or temperature. At FLIR Systems, the calibration of uncooled microbolometer cameras with a

15. http://www.bipm.org/en/about-us/ [Retrieved 2017-01-31.]
16. http://jcgm.bipm.org/vim/en/2.39.html [Retrieved 2017-01-31.]
17. http://jcgm.bipm.org/vim/en/4.30.html [Retrieved 2017-01-31.]
18. http://jcgm.bipm.org/vim/en/4.31.html [Retrieved 2017-01-31.]

measurement capability is carried out during both production and service. Cooled cameras with photon detectors are often calibrated by the user with special software. With this type of software, in theory, common handheld uncooled thermal cameras could be calibrated by the user too. However, as this software is not suitable for reporting purposes, most users do not have it. Non-measuring devices that are used for imaging only do not need temperature calibration. Sometimes this is also reflected in camera terminology when talking about infrared or thermal imaging cameras compared with thermography cameras, where the latter are the measuring devices.

The calibration information, no matter if the calibration is done by FLIR Systems or the user, is stored in calibration curves, which are expressed by mathematical functions. As radiation intensity changes with both temperature and the distance between the object and the camera, different curves are generated for different temperature ranges and exchangeable lenses.

17.4 The differences between a calibration performed by a user and that performed directly at FLIR Systems

First, the reference sources that FLIR Systems uses are themselves calibrated and traceable. This means, at each FLIR Systems site performing calibration, that the sources are controlled by an independent national authority. The camera calibration certificate is confirmation of this. It is proof that not only has the calibration been performed by FLIR Systems but that it has also been carried out using calibrated references. Some users own or have access to accredited reference sources, but they are very few in number.

Second, there is a technical difference. When performing a user calibration, the result is often (but not always) not drift compensated. This means that the values do not take into account a possible change in the camera's output when the camera's internal temperature varies. This yields a larger uncertainty. Drift compensation uses data obtained in climate-controlled chambers. All FLIR Systems cameras are drift compensated when they are first delivered to the customer and when they are recalibrated by FLIR Systems service departments.

17.5 Calibration, verification and adjustment

A common misconception is to confuse *calibration* with *verification* or *adjustment*. Indeed, calibration is a prerequisite for *verification*, which provides confirmation that specified requirements are met. Verification provides objective evidence that a given item fulfills specified requirements. To obtain the verification, defined temperatures (emitted radiation) of calibrated and traceable reference sources are measured. The measurement results, including the deviation, are noted in a table. The verification certificate states that these measurement results meet specified requirements. Sometimes, companies or organizations offer and market this verification certificate as a "calibration certificate."

Proper verification—and by extension calibration and/or recalibration—can only be achieved when a validated protocol is respected. The process is more than placing the camera in front of blackbodies and checking if the camera output (as temperature, for instance) corresponds to the original calibration table. It is often forgotten that a camera is not sensitive to temperature but to radiation. Furthermore, a camera is an *imaging* system, not just a single sensor. Consequently, if the optical configuration allowing the camera to "collect" radiance is poor or misaligned, then the "verification" (or calibration or recalibration) is worthless.

For instance, one has to ensure that the distance between the blackbody and the camera as well as the diameter of the blackbody cavity are chosen so as to reduce stray radiation and the size-of-source effect.

To summarize: a validated protocol must comply with the physical laws for *radiance*, and not only those for temperature.

Calibration is also a prerequisite for *adjustment*, which is the set of operations carried out on a measuring system such that the system provides prescribed indications corresponding to given values of quantities to be measured, typically obtained from measurement standards. Simplified, adjustment is a manipulation that results in instruments that measure correctly within their specifications. In everyday language, the term “calibration” is widely used instead of “adjustment” for measuring devices.

17.6 Non-uniformity correction

When the thermal camera displays ”Calibrating…” it is adjusting for the deviation in response of each individual detector element (pixel). In thermography, this is called a ”non-uniformity correction” (NUC). It is an offset update, and the gain remains unchanged.

The European standard EN 16714-3, Non-destructive Testing—Thermographic Testing—Part 3: Terms and Definitions, defines an NUC as “Image correction carried out by the camera software to compensate for different sensitivities of detector elements and other optical and geometrical disturbances.”

During the NUC (the offset update), a shutter (internal flag) is placed in the optical path, and all the detector elements are exposed to the same amount of radiation originating from the shutter. Therefore, in an ideal situation, they should all give the same output signal. However, each individual element has its own response, so the output is not uniform. This deviation from the ideal result is calculated and used to mathematically perform an image correction, which is essentially a correction of the displayed radiation signal. Some cameras do not have an internal flag. In this case, the offset update must be performed manually using special software and an external uniform source of radiation.

An NUC is performed, for example, at start-up, when changing a measurement range, or when the environment temperature changes. Some cameras also allow the user to trigger it manually. This is useful when you have to perform a critical measurement with as little image disturbance as possible.

17.7 Thermal image adjustment (thermal tuning)

Some people use the term “image calibration” when adjusting the thermal contrast and brightness in the image to enhance specific details. During this operation, the temperature interval is set in such a way that all available colors are used to show only (or mainly) the temperatures in the region of interest. The correct term for this manipulation is “thermal image adjustment” or “thermal tuning”, or, in some languages, “thermal image optimization.” You must be in manual mode to undertake this, otherwise the camera will set the lower and upper limits of the displayed temperature interval automatically to the coldest and hottest temperatures in the scene.

18 History of infrared technology

Before the year 1800, the existence of the infrared portion of the electromagnetic spectrum wasn't even suspected. The original significance of the infrared spectrum, or simply 'the infrared' as it is often called, as a form of heat radiation is perhaps less obvious today than it was at the time of its discovery by Herschel in 1800.

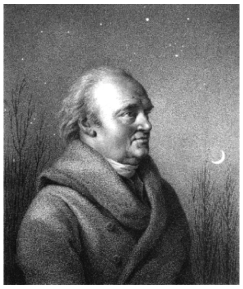

Figure 18.1 Sir William Herschel (1738–1822)

The discovery was made accidentally during the search for a new optical material. Sir William Herschel – Royal Astronomer to King George III of England, and already famous for his discovery of the planet Uranus – was searching for an optical filter material to reduce the brightness of the sun's image in telescopes during solar observations. While testing different samples of colored glass which gave similar reductions in brightness he was intrigued to find that some of the samples passed very little of the sun's heat, while others passed so much heat that he risked eye damage after only a few seconds' observation.

Herschel was soon convinced of the necessity of setting up a systematic experiment, with the objective of finding a single material that would give the desired reduction in brightness as well as the maximum reduction in heat. He began the experiment by actually repeating Newton's prism experiment, but looking for the heating effect rather than the visual distribution of intensity in the spectrum. He first blackened the bulb of a sensitive mercury-in-glass thermometer with ink, and with this as his radiation detector he proceeded to test the heating effect of the various colors of the spectrum formed on the top of a table by passing sunlight through a glass prism. Other thermometers, placed outside the sun's rays, served as controls.

As the blackened thermometer was moved slowly along the colors of the spectrum, the temperature readings showed a steady increase from the violet end to the red end. This was not entirely unexpected, since the Italian researcher, Landriani, in a similar experiment in 1777 had observed much the same effect. It was Herschel, however, who was the first to recognize that there must be a point where the heating effect reaches a maximum, and that measurements confined to the visible portion of the spectrum failed to locate this point.

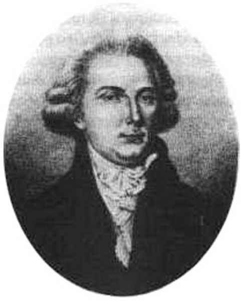

Figure 18.2 Marsilio Landriani (1746–1815)

Moving the thermometer into the dark region beyond the red end of the spectrum, Herschel confirmed that the heating continued to increase. The maximum point, when he found it, lay well beyond the red end – in what is known today as the ‘infrared wavelengths’.

When Herschel revealed his discovery, he referred to this new portion of the electromagnetic spectrum as the ‘thermometrical spectrum’. The radiation itself he sometimes referred to as ‘dark heat’, or simply ‘the invisible rays’. Ironically, and contrary to popular opinion, it wasn't Herschel who originated the term ‘infrared’. The word only began to appear in print around 75 years later, and it is still unclear who should receive credit as the originator.

Herschel’s use of glass in the prism of his original experiment led to some early controversies with his contemporaries about the actual existence of the infrared wavelengths. Different investigators, in attempting to confirm his work, used various types of glass indiscriminately, having different transparencies in the infrared. Through his later experiments, Herschel was aware of the limited transparency of glass to the newly-discovered thermal radiation, and he was forced to conclude that optics for the infrared would probably be doomed to the use of reflective elements exclusively (i.e. plane and curved mirrors). Fortunately, this proved to be true only until 1830, when the Italian investigator, Melloni, made his great discovery that naturally occurring rock salt ($NaCl$) – which was available in large enough natural crystals to be made into lenses and prisms – is remarkably transparent to the infrared. The result was that rock salt became the principal infrared optical material, and remained so for the next hundred years, until the art of synthetic crystal growing was mastered in the 1930’s.

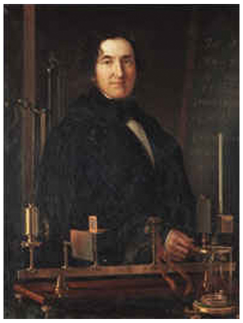

Figure 18.3 Macedonio Melloni (1798–1854)

Thermometers, as radiation detectors, remained unchallenged until 1829, the year Nobili invented the thermocouple. (Herschel’s own thermometer could be read to 0.2 °C (0.036 °F), and later models were able to be read to 0.05 °C (0.09 °F)). Then a breakthrough occurred; Melloni connected a number of thermocouples in series to form the first thermopile. The new device was at least 40 times as sensitive as the best thermometer of the day for detecting heat radiation – capable of detecting the heat from a person standing three meters away.

The first so-called ‘heat-picture’ became possible in 1840, the result of work by Sir John Herschel, son of the discoverer of the infrared and a famous astronomer in his own right. Based upon the differential evaporation of a thin film of oil when exposed to a heat pattern focused upon it, the thermal image could be seen by reflected light where the interference effects of the oil film made the image visible to the eye. Sir John also managed to obtain a primitive record of the thermal image on paper, which he called a ‘thermograph’.

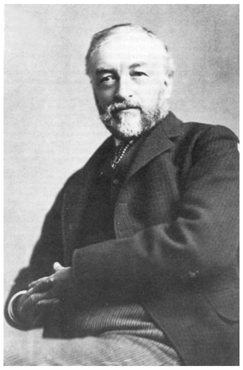

Figure 18.4 Samuel P. Langley (1834–1906)

The improvement of infrared-detector sensitivity progressed slowly. Another major breakthrough, made by Langley in 1880, was the invention of the bolometer. This consisted of a thin blackened strip of platinum connected in one arm of a Wheatstone bridge circuit upon which the infrared radiation was focused and to which a sensitive galvanometer responded. This instrument is said to have been able to detect the heat from a cow at a distance of 400 meters.

An English scientist, Sir James Dewar, first introduced the use of liquefied gases as cooling agents (such as liquid nitrogen with a temperature of –196°C (–320.8°F)) in low temperature research. In 1892 he invented a unique vacuum insulating container in which it is possible to store liquefied gases for entire days. The common 'thermos bottle', used for storing hot and cold drinks, is based upon his invention.

Between the years 1900 and 1920, the inventors of the world 'discovered' the infrared. Many patents were issued for devices to detect personnel, artillery, aircraft, ships – and even icebergs. The first operating systems, in the modern sense, began to be developed during the 1914–18 war, when both sides had research programs devoted to the military exploitation of the infrared. These programs included experimental systems for enemy intrusion/detection, remote temperature sensing, secure communications, and 'flying torpedo' guidance. An infrared search system tested during this period was able to detect an approaching airplane at a distance of 1.5 km (0.94 miles), or a person more than 300 meters (984 ft.) away.

The most sensitive systems up to this time were all based upon variations of the bolometer idea, but the period between the two wars saw the development of two revolutionary new infrared detectors: the image converter and the photon detector. At first, the image converter received the greatest attention by the military, because it enabled an observer for the first time in history to literally 'see in the dark'. However, the sensitivity of the image converter was limited to the near infrared wavelengths, and the most interesting military targets (i.e. enemy soldiers) had to be illuminated by infrared search beams. Since this involved the risk of giving away the observer's position to a similarly-equipped enemy observer, it is understandable that military interest in the image converter eventually faded.

The tactical military disadvantages of so-called 'active' (i.e. search beam-equipped) thermal imaging systems provided impetus following the 1939–45 war for extensive secret military infrared-research programs into the possibilities of developing 'passive' (no search beam) systems around the extremely sensitive photon detector. During this period, military secrecy regulations completely prevented disclosure of the status of infrared-imaging technology. This secrecy only began to be lifted in the middle of the 1950's, and from that time adequate thermal-imaging devices finally began to be available to civilian science and industry.

19 Theory of thermography

19.1 Introduction

The subjects of infrared radiation and the related technique of thermography are still new to many who will use an infrared camera. In this section the theory behind thermography will be given.

19.2 The electromagnetic spectrum

The electromagnetic spectrum is divided arbitrarily into a number of wavelength regions, called *bands*, distinguished by the methods used to produce and detect the radiation. There is no fundamental difference between radiation in the different bands of the electromagnetic spectrum. They are all governed by the same laws and the only differences are those due to differences in wavelength.

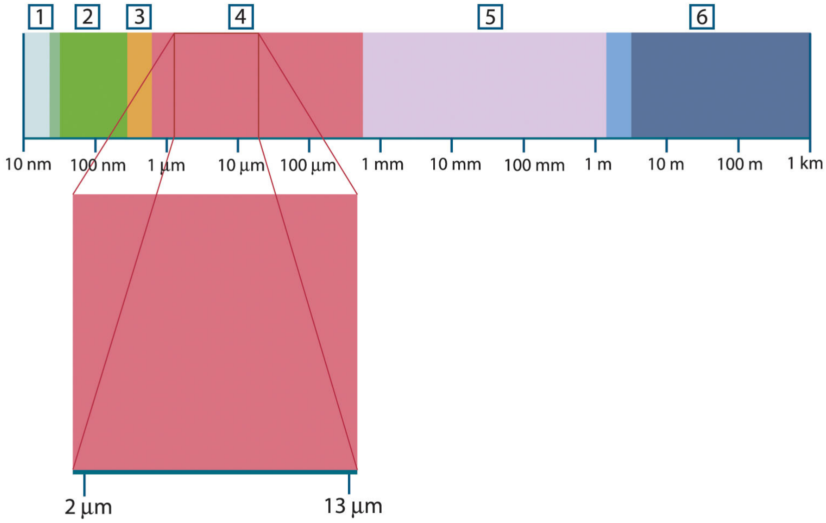

Figure 19.1 The electromagnetic spectrum. 1: X-ray; 2: UV; 3: Visible; 4: IR; 5: Microwaves; 6: Radiowaves.

Thermography makes use of the infrared spectral band. At the short-wavelength end the boundary lies at the limit of visual perception, in the deep red. At the long-wavelength end it merges with the microwave radio wavelengths, in the millimeter range.

The infrared band is often further subdivided into four smaller bands, the boundaries of which are also arbitrarily chosen. They include: the *near infrared* (0.75–3 µm), the *middle infrared* (3–6 µm), the *far infrared* (6–15 µm) and the *extreme infrared* (15–100 µm). Although the wavelengths are given in µm (micrometers), other units are often still used to measure wavelength in this spectral region, *e.g.* nanometer (nm) and Ångström (Å).

The relationships between the different wavelength measurements is:

$$10\ 000\ \text{Å} = 1\ 000\ \text{nm} = 1\ \mu = 1\ \mu\text{m}$$

19.3 Blackbody radiation

A blackbody is defined as an object which absorbs all radiation that impinges on it at any wavelength. The apparent misnomer *black* relating to an object emitting radiation is explained by Kirchhoff's Law (after *Gustav Robert Kirchhoff*, 1824–1887), which states that a body capable of absorbing all radiation at any wavelength is equally capable in the emission of radiation.

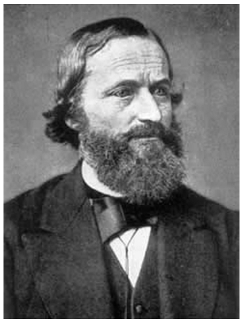

Figure 19.2 Gustav Robert Kirchhoff (1824–1887)

The construction of a blackbody source is, in principle, very simple. The radiation characteristics of an aperture in an isotherm cavity made of an opaque absorbing material represents almost exactly the properties of a blackbody. A practical application of the principle to the construction of a perfect absorber of radiation consists of a box that is light tight except for an aperture in one of the sides. Any radiation which then enters the hole is scattered and absorbed by repeated reflections so only an infinitesimal fraction can possibly escape. The blackness which is obtained at the aperture is nearly equal to a blackbody and almost perfect for all wavelengths.

By providing such an isothermal cavity with a suitable heater it becomes what is termed a *cavity radiator*. An isothermal cavity heated to a uniform temperature generates blackbody radiation, the characteristics of which are determined solely by the temperature of the cavity. Such cavity radiators are commonly used as sources of radiation in temperature reference standards in the laboratory for calibrating thermographic instruments, such as a FLIR Systems camera for example.

If the temperature of blackbody radiation increases to more than 525°C (977°F), the source begins to be visible so that it appears to the eye no longer black. This is the incipient red heat temperature of the radiator, which then becomes orange or yellow as the temperature increases further. In fact, the definition of the so-called *color temperature* of an object is the temperature to which a blackbody would have to be heated to have the same appearance.

Now consider three expressions that describe the radiation emitted from a blackbody.

19.3.1 Planck's law

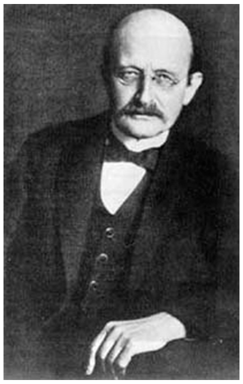

Figure 19.3 Max Planck (1858–1947)

Max Planck (1858–1947) was able to describe the spectral distribution of the radiation from a blackbody by means of the following formula:

$$W_{\lambda b} = \frac{2\pi hc^2}{\lambda^5 \left(e^{hc/\lambda kT} - 1\right)} \times 10^{-6} [Watt / m^2, \mu m]$$

where:

$W_{\lambda b}$	Blackbody spectral radiant emittance at wavelength λ.
c	Velocity of light = 3×10^8 m/s
h	Planck's constant = 6.6×10^{-34} Joule sec.
k	Boltzmann's constant = 1.4×10^{-23} Joule/K.
T	Absolute temperature (K) of a blackbody.
λ	Wavelength (μm).

Note The factor 10^{-6} is used since spectral emittance in the curves is expressed in Watt/ m^2, μm.

Planck's formula, when plotted graphically for various temperatures, produces a family of curves. Following any particular Planck curve, the spectral emittance is zero at $\lambda = 0$, then increases rapidly to a maximum at a wavelength λ_{max} and after passing it approaches zero again at very long wavelengths. The higher the temperature, the shorter the wavelength at which maximum occurs.

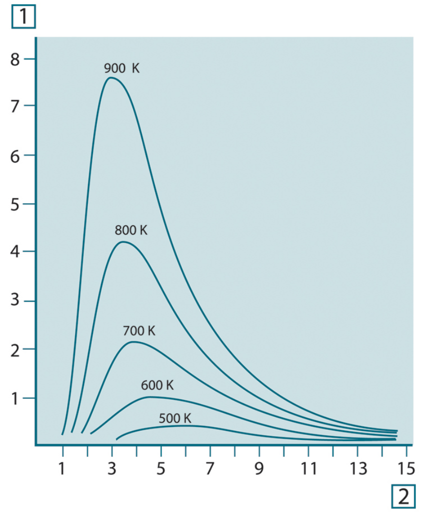

Figure 19.4 Blackbody spectral radiant emittance according to Planck's law, plotted for various absolute temperatures. 1: Spectral radiant emittance (W/cm² × 10³(μm)); 2: Wavelength (μm)

19.3.2 Wien's displacement law

By differentiating Planck's formula with respect to λ, and finding the maximum, we have:

$$\lambda_{\mathrm{max}} = \frac{2898}{T}[\mu m]$$

This is Wien's formula (after *Wilhelm Wien*, 1864–1928), which expresses mathematically the common observation that colors vary from red to orange or yellow as the temperature of a thermal radiator increases. The wavelength of the color is the same as the wavelength calculated for λ_{max}. A good approximation of the value of λ_{max} for a given blackbody temperature is obtained by applying the rule-of-thumb 3 000/T μm. Thus, a very hot star such as Sirius (11 000 K), emitting bluish-white light, radiates with the peak of spectral radiant emittance occurring within the invisible ultraviolet spectrum, at wavelength 0.27 μm.

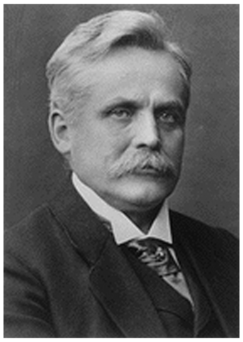

Figure 19.5 Wilhelm Wien (1864–1928)

The sun (approx. 6 000 K) emits yellow light, peaking at about 0.5 μm in the middle of the visible light spectrum.

At room temperature (300 K) the peak of radiant emittance lies at 9.7 µm, in the far infrared, while at the temperature of liquid nitrogen (77 K) the maximum of the almost insignificant amount of radiant emittance occurs at 38 µm, in the extreme infrared wavelengths.

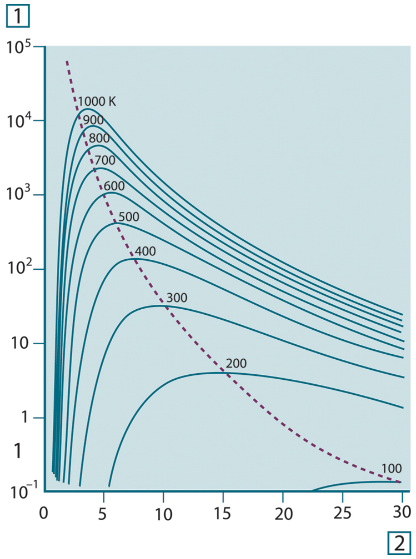

Figure 19.6 Planckian curves plotted on semi-log scales from 100 K to 1000 K. The dotted line represents the locus of maximum radiant emittance at each temperature as described by Wien's displacement law. 1: Spectral radiant emittance (W/cm² (µm)); 2: Wavelength (µm).

19.3.3 Stefan-Boltzmann's law

By integrating Planck's formula from λ = 0 to λ = ∞, we obtain the total radiant emittance (W_b) of a blackbody:

$$W_b = \sigma T^4 \ [\text{Watt/m}^2]$$

This is the Stefan-Boltzmann formula (after *Josef Stefan*, 1835–1893, and *Ludwig Boltzmann*, 1844–1906), which states that the total emissive power of a blackbody is proportional to the fourth power of its absolute temperature. Graphically, W_b represents the area below the Planck curve for a particular temperature. It can be shown that the radiant emittance in the interval λ = 0 to λ_{max} is only 25% of the total, which represents about the amount of the sun's radiation which lies inside the visible light spectrum.

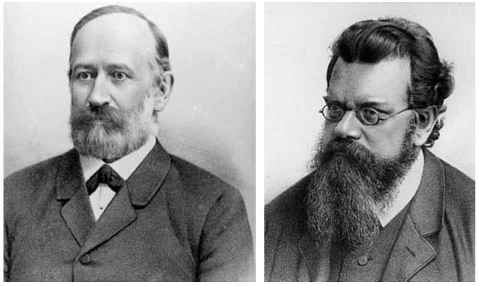

Figure 19.7 Josef Stefan (1835–1893), and Ludwig Boltzmann (1844–1906)

Using the Stefan-Boltzmann formula to calculate the power radiated by the human body, at a temperature of 300 K and an external surface area of approx. 2 m^2, we obtain 1 kW. This power loss could not be sustained if it were not for the compensating absorption of radiation from surrounding surfaces, at room temperatures which do not vary too drastically from the temperature of the body – or, of course, the addition of clothing.

19.3.4 Non-blackbody emitters

So far, only blackbody radiators and blackbody radiation have been discussed. However, real objects almost never comply with these laws over an extended wavelength region – although they may approach the blackbody behavior in certain spectral intervals. For example, a certain type of white paint may appear perfectly *white* in the visible light spectrum, but becomes distinctly *gray* at about 2 µm, and beyond 3 µm it is almost *black*.

There are three processes which can occur that prevent a real object from acting like a blackbody: a fraction of the incident radiation α may be absorbed, a fraction ρ may be reflected, and a fraction τ may be transmitted. Since all of these factors are more or less wavelength dependent, the subscript λ is used to imply the spectral dependence of their definitions. Thus:

- The spectral absorptance α_λ= the ratio of the spectral radiant power absorbed by an object to that incident upon it.
- The spectral reflectance ρ_λ = the ratio of the spectral radiant power reflected by an object to that incident upon it.
- The spectral transmittance τ_λ = the ratio of the spectral radiant power transmitted through an object to that incident upon it.

The sum of these three factors must always add up to the whole at any wavelength, so we have the relation:

$$\alpha_\lambda + \rho_\lambda + \tau_\lambda = 1$$

For opaque materials τ_λ = 0 and the relation simplifies to:

$$\varepsilon_\lambda + \rho_\lambda = 1$$

Another factor, called the emissivity, is required to describe the fraction ε of the radiant emittance of a blackbody produced by an object at a specific temperature. Thus, we have the definition:

The spectral emissivity ε_λ= the ratio of the spectral radiant power from an object to that from a blackbody at the same temperature and wavelength.

Expressed mathematically, this can be written as the ratio of the spectral emittance of the object to that of a blackbody as follows:

$$\varepsilon_\lambda = \frac{W_{\lambda o}}{W_{\lambda b}}$$

Generally speaking, there are three types of radiation source, distinguished by the ways in which the spectral emittance of each varies with wavelength.

- A blackbody, for which $\varepsilon_\lambda = \varepsilon = 1$
- A graybody, for which $\varepsilon_\lambda = \varepsilon$ = constant less than 1
- A selective radiator, for which ε varies with wavelength

According to Kirchhoff's law, for any material the spectral emissivity and spectral absorptance of a body are equal at any specified temperature and wavelength. That is:

$$\varepsilon_\lambda = \alpha_\lambda$$

From this we obtain, for an opaque material (since $\alpha_\lambda + \rho_\lambda = 1$):

$$\varepsilon_\lambda + \rho_\lambda = 1$$

For highly polished materials ελ approaches zero, so that for a perfectly reflecting material (*i.e.* a perfect mirror) we have:

$$\rho_\lambda = 1$$

For a graybody radiator, the Stefan-Boltzmann formula becomes:

$$W = \varepsilon\sigma T^4 \ [\mathrm{Watt/m^2}]$$

This states that the total emissive power of a graybody is the same as a blackbody at the same temperature reduced in proportion to the value of ε from the graybody.

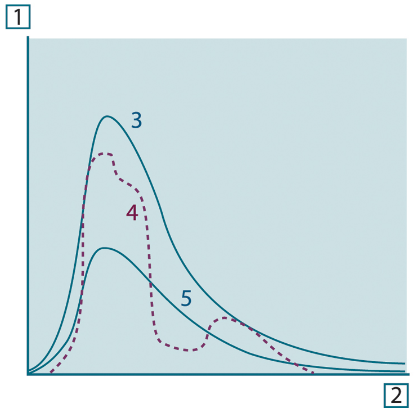

Figure 19.8 Spectral radiant emittance of three types of radiators. 1: Spectral radiant emittance; 2: Wavelength; 3: Blackbody; 4: Selective radiator; 5: Graybody.

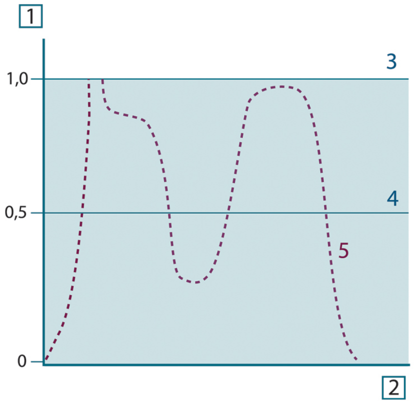

Figure 19.9 Spectral emissivity of three types of radiators. 1: Spectral emissivity; 2: Wavelength; 3: Blackbody; 4: Graybody; 5: Selective radiator.

19.4 Infrared semi-transparent materials

Consider now a non-metallic, semi-transparent body – let us say, in the form of a thick flat plate of plastic material. When the plate is heated, radiation generated within its volume must work its way toward the surfaces through the material in which it is partially absorbed. Moreover, when it arrives at the surface, some of it is reflected back into the interior. The back-reflected radiation is again partially absorbed, but some of it arrives at the other surface, through which most of it escapes; part of it is reflected back again. Although the progressive reflections become weaker and weaker they must all be added up when the total emittance of the plate is sought. When the resulting geometrical series is summed, the effective emissivity of a semi-transparent plate is obtained as:

$$\varepsilon_\lambda = \frac{(1-\rho_\lambda)(1-\tau_\lambda)}{1-\rho_\lambda\tau_\lambda}$$

When the plate becomes opaque this formula is reduced to the single formula:

$$\varepsilon_\lambda = 1-\rho_\lambda$$

This last relation is a particularly convenient one, because it is often easier to measure reflectance than to measure emissivity directly.

20 The measurement formula

As already mentioned, when viewing an object, the camera receives radiation not only from the object itself. It also collects radiation from the surroundings reflected via the object surface. Both these radiation contributions become attenuated to some extent by the atmosphere in the measurement path. To this comes a third radiation contribution from the atmosphere itself.

This description of the measurement situation, as illustrated in the figure below, is so far a fairly true description of the real conditions. What has been neglected could for instance be sun light scattering in the atmosphere or stray radiation from intense radiation sources outside the field of view. Such disturbances are difficult to quantify, however, in most cases they are fortunately small enough to be neglected. In case they are not negligible, the measurement configuration is likely to be such that the risk for disturbance is obvious, at least to a trained operator. It is then his responsibility to modify the measurement situation to avoid the disturbance e.g. by changing the viewing direction, shielding off intense radiation sources etc.

Accepting the description above, we can use the figure below to derive a formula for the calculation of the object temperature from the calibrated camera output.

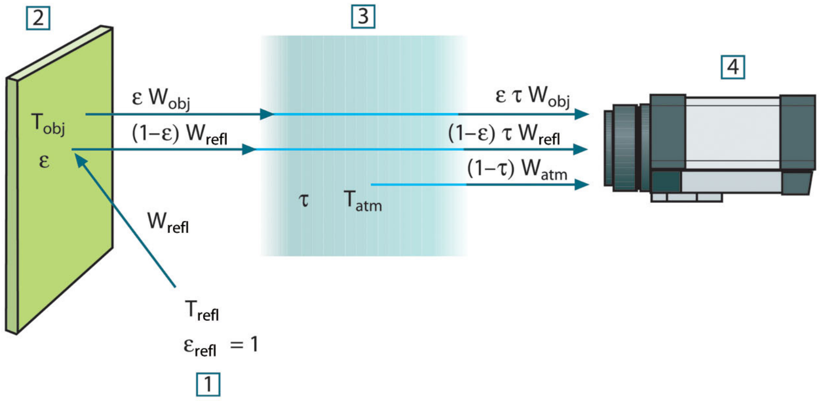

Figure 20.1 A schematic representation of the general thermographic measurement situation.1: Surroundings; 2: Object; 3: Atmosphere; 4: Camera

Assume that the received radiation power W from a blackbody source of temperature T_{source} on short distance generates a camera output signal U_{source} that is proportional to the power input (power linear camera). We can then write (Equation 1):

$$U_{source} = CW(T_{source})$$

or, with simplified notation:

$$U_{source} = CW_{source}$$

where C is a constant.

Should the source be a graybody with emittance ε, the received radiation would consequently be εW_{source}.

We are now ready to write the three collected radiation power terms:

1. *Emission from the object* = $\varepsilon\tau W_{obj}$, where ε is the emittance of the object and τ is the transmittance of the atmosphere. The object temperature is T_{obj}.

2. *Reflected emission from ambient sources* = $(1 - \varepsilon)\tau W_{refl}$, where $(1 - \varepsilon)$ is the reflectance of the object. The ambient sources have the temperature T_{refl}.
   It has here been assumed that the temperature T_{refl} is the same for all emitting surfaces within the halfsphere seen from a point on the object surface. This is of course sometimes a simplification of the true situation. It is, however, a necessary simplification in order to derive a workable formula, and T_{refl} can – at least theoretically – be given a value that represents an efficient temperature of a complex surrounding.

   Note also that we have assumed that the emittance for the surroundings = 1. This is correct in accordance with Kirchhoff's law: All radiation impinging on the surrounding surfaces will eventually be absorbed by the same surfaces. Thus the emittance = 1. (Note though that the latest discussion requires the complete sphere around the object to be considered.)
3. *Emission from the atmosphere* = $(1 - \tau)\tau W_{atm}$, where $(1 - \tau)$ is the emittance of the atmosphere. The temperature of the atmosphere is T_{atm}.

The total received radiation power can now be written (Equation 2):

$$W_{tot} = \varepsilon\tau W_{obj} + (1-\varepsilon)\tau W_{rcfl} + (1-\tau)W_{atm}$$

We multiply each term by the constant C of Equation 1 and replace the CW products by the corresponding U according to the same equation, and get (Equation 3):

$$U_{tot} = \varepsilon\tau U_{obj} + (1-\varepsilon)\tau U_{rcfl} + (1-\tau)U_{atm}$$

Solve Equation 3 for U_{obj} (Equation 4):

$$U_{obj} = \frac{1}{\varepsilon\tau}U_{tot} - \frac{1-\varepsilon}{\varepsilon}U_{rcfl} - \frac{1-\tau}{\varepsilon\tau}U_{atm}$$

This is the general measurement formula used in all the FLIR Systems thermographic equipment. The voltages of the formula are:

Table 20.1 Voltages

U_{obj}	Calculated camera output voltage for a blackbody of temperature T_{obj} i.e. a voltage that can be directly converted into true requested object temperature.
U_{tot}	Measured camera output voltage for the actual case.
U_{refl}	Theoretical camera output voltage for a blackbody of temperature T_{refl} according to the calibration.
U_{atm}	Theoretical camera output voltage for a blackbody of temperature T_{atm} according to the calibration.

The operator has to supply a number of parameter values for the calculation:

- the object emittance ε,
- the relative humidity,
- T_{atm}
- object distance (D_{obj})
- the (effective) temperature of the object surroundings, or the reflected ambient temperature T_{refl}, and
- the temperature of the atmosphere T_{atm}

This task could sometimes be a heavy burden for the operator since there are normally no easy ways to find accurate values of emittance and atmospheric transmittance for the

actual case. The two temperatures are normally less of a problem provided the surroundings do not contain large and intense radiation sources.

A natural question in this connection is: How important is it to know the right values of these parameters? It could though be of interest to get a feeling for this problem already here by looking into some different measurement cases and compare the relative magnitudes of the three radiation terms. This will give indications about when it is important to use correct values of which parameters.

The figures below illustrates the relative magnitudes of the three radiation contributions for three different object temperatures, two emittances, and two spectral ranges: SW and LW. Remaining parameters have the following fixed values:

- $\tau = 0.88$
- T_{refl} = +20°C (+68°F)
- T_{atm} = +20°C (+68°F)

It is obvious that measurement of low object temperatures are more critical than measuring high temperatures since the ‘disturbing’ radiation sources are relatively much stronger in the first case. Should also the object emittance be low, the situation would be still more difficult.

We have finally to answer a question about the importance of being allowed to use the calibration curve above the highest calibration point, what we call extrapolation. Imagine that we in a certain case measure U_{tot} = 4.5 volts. The highest calibration point for the camera was in the order of 4.1 volts, a value unknown to the operator. Thus, even if the object happened to be a blackbody, i.e. $U_{obj} = U_{tot}$, we are actually performing extrapolation of the calibration curve when converting 4.5 volts into temperature.

Let us now assume that the object is not black, it has an emittance of 0.75, and the transmittance is 0.92. We also assume that the two second terms of Equation 4 amount to 0.5 volts together. Computation of U_{obj} by means of Equation 4 then results in U_{obj} = 4.5 / 0.75 / 0.92 – 0.5 = 6.0. This is a rather extreme extrapolation, particularly when considering that the video amplifier might limit the output to 5 volts! Note, though, that the application of the calibration curve is a theoretical procedure where no electronic or other limitations exist. We trust that if there had been no signal limitations in the camera, and if it had been calibrated far beyond 5 volts, the resulting curve would have been very much the same as our real curve extrapolated beyond 4.1 volts, provided the calibration algorithm is based on radiation physics, like the FLIR Systems algorithm. Of course there must be a limit to such extrapolations.

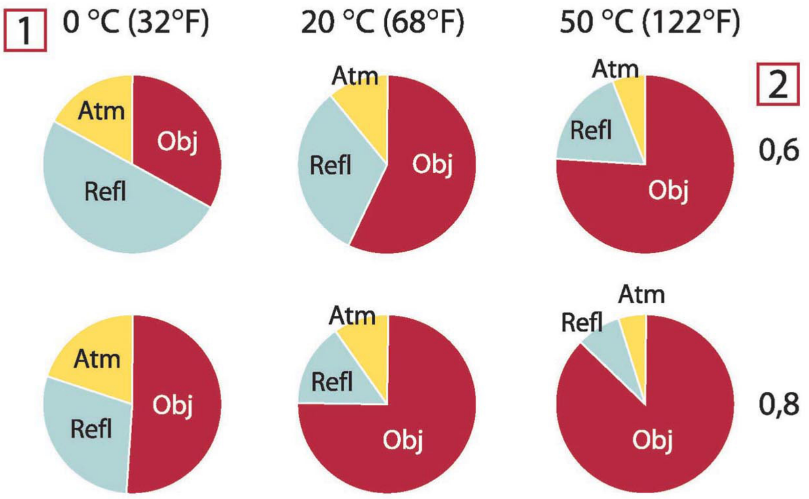

Figure 20.2 Relative magnitudes of radiation sources under varying measurement conditions (SW camera). 1: Object temperature; 2: Emittance; Obj: Object radiation; Refl: Reflected radiation; Atm: atmosphere radiation. Fixed parameters: τ = 0.88; T_{refl} = 20°C (+68°F); T_{atm} = 20°C (+68°F).

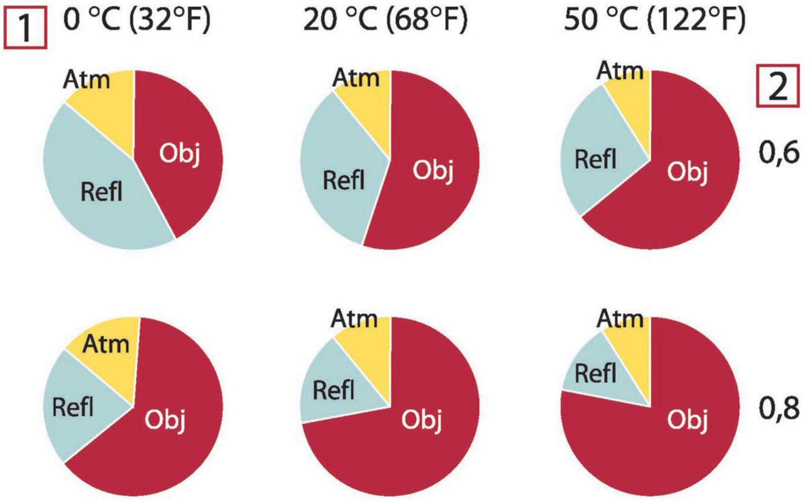

Figure 20.3 Relative magnitudes of radiation sources under varying measurement conditions (LW camera). 1: Object temperature; 2: Emittance; Obj: Object radiation; Refl: Reflected radiation; Atm: atmosphere radiation. Fixed parameters: τ = 0.88; T_{refl} = 20°C (+68°F); T_{atm} = 20°C (+68°F).

21 Emissivity tables

This section presents a compilation of emissivity data from the infrared literature and measurements made by FLIR Systems.

21.1 References

1. Mikaél A. Bramson: *Infrared Radiation, A Handbook for Applications*, Plenum press, N. Y.
2. William L. Wolfe, George J. Zissis: *The Infrared Handbook*, Office of Naval Research, Department of Navy, Washington, D.C.
3. Madding, R. P.: *Thermographic Instruments and systems*. Madison, Wisconsin: University of Wisconsin – Extension, Department of Engineering and Applied Science.
4. William L. Wolfe: *Handbook of Military Infrared Technology*, Office of Naval Research, Department of Navy, Washington, D.C.
5. Jones, Smith, Probert: *External thermography of buildings...,* Proc. of the Society of Photo-Optical Instrumentation Engineers, vol.110, Industrial and Civil Applications of Infrared Technology, June 1977 London.
6. Paljak, Pettersson: *Thermography of Buildings*, Swedish Building Research Institute, Stockholm 1972.
7. Vlcek, J: *Determination of emissivity with imaging radiometers and some emissivities at λ = 5 µm.* Photogrammetric Engineering and Remote Sensing.
8. Kern: *Evaluation of infrared emission of clouds and ground as measured by weather satellites*, Defence Documentation Center, AD 617 417.
9. Öhman, Claes: *Emittansmätningar med AGEMA E-Box*. Teknisk rapport, AGEMA 1999. (Emittance measurements using AGEMA E-Box. Technical report, AGEMA 1999.)
10. Matteï, S., Tang-Kwor, E: *Emissivity measurements for Nextel Velvet coating 811-21 between –36°C AND 82°C.*
11. Lohrengel & Todtenhaupt (1996)
12. ITC Technical publication 32.
13. ITC Technical publication 29.
14. Schuster, Norbert and Kolobrodov, Valentin G. *Infrarotthermographie*. Berlin: Wiley-VCH, 2000.

Note The emissivity values in the table below are recorded using a shortwave (SW) camera. The values should be regarded as recommendations only and used with caution.

21.2 Tables

Table 21.1 T: Total spectrum; SW: 2–5 µm; LW: 8–14 µm, LLW: 6.5–20 µm; 1: Material; 2: Specification; 3: Temperature in °C; 4: Spectrum; 5: Emissivity: 6:Reference

1	2	3	4	5	6
3M type 35	Vinyl electrical tape (several colors)	< 80	LW	≈ 0.96	13
3M type 88	Black vinyl electrical tape	< 105	LW	≈ 0.96	13
3M type 88	Black vinyl electrical tape	< 105	MW	< 0.96	13
3M type Super 33 +	Black vinyl electrical tape	< 80	LW	≈ 0.96	13
Aluminum	anodized sheet	100	T	0.55	2

Table 21.1 T: Total spectrum; SW: 2–5 µm; LW: 8–14 µm, LLW: 6.5–20 µm; 1: Material; 2: Specification; 3: Temperature in °C; 4: Spectrum; 5: Emissivity: 6:Reference (continued)

1	2	3	4	5	6
Aluminum	anodized, black, dull	70	SW	0.67	9
Aluminum	anodized, black, dull	70	LW	0.95	9
Aluminum	anodized, light gray, dull	70	SW	0.61	9
Aluminum	anodized, light gray, dull	70	LW	0.97	9
Aluminum	as received, plate	100	T	0.09	4
Aluminum	as received, sheet	100	T	0.09	2
Aluminum	cast, blast cleaned	70	SW	0.47	9
Aluminum	cast, blast cleaned	70	LW	0.46	9
Aluminum	dipped in HNO_3, plate	100	T	0.05	4
Aluminum	foil	27	10 µm	0.04	3
Aluminum	foil	27	3 µm	0.09	3
Aluminum	oxidized, strongly	50–500	T	0.2–0.3	1
Aluminum	polished	50–100	T	0.04–0.06	1
Aluminum	polished plate	100	T	0.05	4
Aluminum	polished, sheet	100	T	0.05	2
Aluminum	rough surface	20–50	T	0.06–0.07	1
Aluminum	roughened	27	10 µm	0.18	3
Aluminum	roughened	27	3 µm	0.28	3
Aluminum	sheet, 4 samples differently scratched	70	SW	0.05–0.08	9
Aluminum	sheet, 4 samples differently scratched	70	LW	0.03–0.06	9
Aluminum	vacuum deposited	20	T	0.04	2
Aluminum	weathered, heavily	17	SW	0.83–0.94	5
Aluminum bronze		20	T	0.60	1
Aluminum hydroxide	powder		T	0.28	1
Aluminum oxide	activated, powder		T	0.46	1
Aluminum oxide	pure, powder (alumina)		T	0.16	1
Asbestos	board	20	T	0.96	1
Asbestos	fabric		T	0.78	1
Asbestos	floor tile	35	SW	0.94	7
Asbestos	paper	40–400	T	0.93–0.95	1

Table 21.1 T: Total spectrum; SW: 2–5 µm; LW: 8–14 µm, LLW: 6.5–20 µm; 1: Material; 2: Specification; 3: Temperature in °C; 4: Spectrum; 5: Emissivity: 6:Reference (continued)

1	2	3	4	5	6
Asbestos	powder		T	0.40–0.60	1
Asbestos	slate	20	T	0.96	1
Asphalt paving		4	LLW	0.967	8
Brass	dull, tarnished	20–350	T	0.22	1
Brass	oxidized	100	T	0.61	2
Brass	oxidized	70	SW	0.04–0.09	9
Brass	oxidized	70	LW	0.03–0.07	9
Brass	oxidized at 600°C	200–600	T	0.59–0.61	1
Brass	polished	200	T	0.03	1
Brass	polished, highly	100	T	0.03	2
Brass	rubbed with 80-grit emery	20	T	0.20	2
Brass	sheet, rolled	20	T	0.06	1
Brass	sheet, worked with emery	20	T	0.2	1
Brick	alumina	17	SW	0.68	5
Brick	common	17	SW	0.86–0.81	5
Brick	Dinas silica, glazed, rough	1100	T	0.85	1
Brick	Dinas silica, refractory	1000	T	0.66	1
Brick	Dinas silica, un-glazed, rough	1000	T	0.80	1
Brick	firebrick	17	SW	0.68	5
Brick	fireclay	1000	T	0.75	1
Brick	fireclay	1200	T	0.59	1
Brick	fireclay	20	T	0.85	1
Brick	masonry	35	SW	0.94	7
Brick	masonry, plastered	20	T	0.94	1
Brick	red, common	20	T	0.93	2
Brick	red, rough	20	T	0.88–0.93	1
Brick	refractory, corundum	1000	T	0.46	1
Brick	refractory, magnesite	1000–1300	T	0.38	1
Brick	refractory, strongly radiating	500–1000	T	0.8–0.9	1
Brick	refractory, weakly radiating	500–1000	T	0.65–0.75	1
Brick	silica, 95% SiO_2	1230	T	0.66	1
Brick	sillimanite, 33% SiO_2, 64% Al_2O_3	1500	T	0.29	1

Table 21.1 T: Total spectrum; SW: 2–5 µm; LW: 8–14 µm, LLW: 6.5–20 µm; 1: Material; 2: Specification; 3: Temperature in °C; 4: Spectrum; 5: Emissivity: 6:Reference (continued)

1	2	3	4	5	6
Brick	waterproof	17	SW	0.87	5
Bronze	phosphor bronze	70	SW	0.08	9
Bronze	phosphor bronze	70	LW	0.06	9
Bronze	polished	50	T	0.1	1
Bronze	porous, rough	50–150	T	0.55	1
Bronze	powder		T	0.76–0.80	1
Carbon	candle soot	20	T	0.95	2
Carbon	charcoal powder		T	0.96	1
Carbon	graphite powder		T	0.97	1
Carbon	graphite, filed surface	20	T	0.98	2
Carbon	lampblack	20–400	T	0.95–0.97	1
Chipboard	untreated	20	SW	0.90	6
Chromium	polished	50	T	0.10	1
Chromium	polished	500–1000	T	0.28–0.38	1
Clay	fired	70	T	0.91	1
Cloth	black	20	T	0.98	1
Concrete		20	T	0.92	2
Concrete	dry	36	SW	0.95	7
Concrete	rough	17	SW	0.97	5
Concrete	walkway	5	LLW	0.974	8
Copper	commercial, burnished	20	T	0.07	1
Copper	electrolytic, care-fully polished	80	T	0.018	1
Copper	electrolytic, polished	–34	T	0.006	4
Copper	molten	1100–1300	T	0.13–0.15	1
Copper	oxidized	50	T	0.6–0.7	1
Copper	oxidized to blackness		T	0.88	1
Copper	oxidized, black	27	T	0.78	4
Copper	oxidized, heavily	20	T	0.78	2
Copper	polished	50–100	T	0.02	1
Copper	polished	100	T	0.03	2
Copper	polished, commercial	27	T	0.03	4
Copper	polished, mechanical	22	T	0.015	4
Copper	pure, carefully prepared surface	22	T	0.008	4
Copper	scraped	27	T	0.07	4

Table 21.1 T: Total spectrum; SW: 2–5 µm; LW: 8–14 µm, LLW: 6.5–20 µm; 1: Material; 2: Specification; 3: Temperature in °C; 4: Spectrum; 5: Emissivity: 6:Reference (continued)

1	2	3	4	5	6
Copper dioxide	powder		T	0.84	1
Copper oxide	red, powder		T	0.70	1
Ebonite			T	0.89	1
Emery	coarse	80	T	0.85	1
Enamel		20	T	0.9	1
Enamel	lacquer	20	T	0.85–0.95	1
Fiber board	hard, untreated	20	SW	0.85	6
Fiber board	masonite	70	SW	0.75	9
Fiber board	masonite	70	LW	0.88	9
Fiber board	particle board	70	SW	0.77	9
Fiber board	particle board	70	LW	0.89	9
Fiber board	porous, untreated	20	SW	0.85	6
Glass pane (float glass)	non-coated	20	LW	0.97	14
Gold	polished	130	T	0.018	1
Gold	polished, carefully	200–600	T	0.02–0.03	1
Gold	polished, highly	100	T	0.02	2
Granite	polished	20	LLW	0.849	8
Granite	rough	21	LLW	0.879	8
Granite	rough, 4 different samples	70	SW	0.95–0.97	9
Granite	rough, 4 different samples	70	LW	0.77–0.87	9
Gypsum		20	T	0.8–0.9	1
Ice: See Water					
Iron and steel	cold rolled	70	SW	0.20	9
Iron and steel	cold rolled	70	LW	0.09	9
Iron and steel	covered with red rust	20	T	0.61–0.85	1
Iron and steel	electrolytic	100	T	0.05	4
Iron and steel	electrolytic	22	T	0.05	4
Iron and steel	electrolytic	260	T	0.07	4
Iron and steel	electrolytic, carefully polished	175–225	T	0.05–0.06	1
Iron and steel	freshly worked with emery	20	T	0.24	1
Iron and steel	ground sheet	950–1100	T	0.55–0.61	1
Iron and steel	heavily rusted sheet	20	T	0.69	2
Iron and steel	hot rolled	130	T	0.60	1
Iron and steel	hot rolled	20	T	0.77	1
Iron and steel	oxidized	100	T	0.74	4

Table 21.1 T: Total spectrum; SW: 2–5 µm; LW: 8–14 µm, LLW: 6.5–20 µm; 1: Material; 2: Specification; 3: Temperature in °C; 4: Spectrum; 5: Emissivity: 6:Reference (continued)

1	2	3	4	5	6
Iron and steel	oxidized	100	T	0.74	1
Iron and steel	oxidized	1227	T	0.89	4
Iron and steel	oxidized	125–525	T	0.78–0.82	1
Iron and steel	oxidized	200	T	0.79	2
Iron and steel	oxidized	200–600	T	0.80	1
Iron and steel	oxidized strongly	50	T	0.88	1
Iron and steel	oxidized strongly	500	T	0.98	1
Iron and steel	polished	100	T	0.07	2
Iron and steel	polished	400–1000	T	0.14–0.38	1
Iron and steel	polished sheet	750–1050	T	0.52–0.56	1
Iron and steel	rolled sheet	50	T	0.56	1
Iron and steel	rolled, freshly	20	T	0.24	1
Iron and steel	rough, plane surface	50	T	0.95–0.98	1
Iron and steel	rusted red, sheet	22	T	0.69	4
Iron and steel	rusted, heavily	17	SW	0.96	5
Iron and steel	rusty, red	20	T	0.69	1
Iron and steel	shiny oxide layer, sheet,	20	T	0.82	1
Iron and steel	shiny, etched	150	T	0.16	1
Iron and steel	wrought, carefully polished	40–250	T	0.28	1
Iron galvanized	heavily oxidized	70	SW	0.64	9
Iron galvanized	heavily oxidized	70	LW	0.85	9
Iron galvanized	sheet	92	T	0.07	4
Iron galvanized	sheet, burnished	30	T	0.23	1
Iron galvanized	sheet, oxidized	20	T	0.28	1
Iron tinned	sheet	24	T	0.064	4
Iron, cast	casting	50	T	0.81	1
Iron, cast	ingots	1000	T	0.95	1
Iron, cast	liquid	1300	T	0.28	1
Iron, cast	machined	800–1000	T	0.60–0.70	1
Iron, cast	oxidized	100	T	0.64	2
Iron, cast	oxidized	260	T	0.66	4
Iron, cast	oxidized	38	T	0.63	4
Iron, cast	oxidized	538	T	0.76	4
Iron, cast	oxidized at 600°C	200–600	T	0.64–0.78	1
Iron, cast	polished	200	T	0.21	1
Iron, cast	polished	38	T	0.21	4
Iron, cast	polished	40	T	0.21	2

Table 21.1 T: Total spectrum; SW: 2–5 µm; LW: 8–14 µm, LLW: 6.5–20 µm; 1: Material; 2: Specification; 3: Temperature in °C; 4: Spectrum; 5: Emissivity: 6:Reference (continued)

1	2	3	4	5	6
Iron, cast	unworked	900–1100	T	0.87–0.95	1
Krylon Ultra-flat black 1602	Flat black	Room temperature up to 175	LW	≈ 0.96	12
Krylon Ultra-flat black 1602	Flat black	Room temperature up to 175	MW	≈ 0.97	12
Lacquer	3 colors sprayed on Aluminum	70	SW	0.50–0.53	9
Lacquer	3 colors sprayed on Aluminum	70	LW	0.92–0.94	9
Lacquer	Aluminum on rough surface	20	T	0.4	1
Lacquer	bakelite	80	T	0.83	1
Lacquer	black, dull	40–100	T	0.96–0.98	1
Lacquer	black, matte	100	T	0.97	2
Lacquer	black, shiny, sprayed on iron	20	T	0.87	1
Lacquer	heat–resistant	100	T	0.92	1
Lacquer	white	100	T	0.92	2
Lacquer	white	40–100	T	0.8–0.95	1
Lead	oxidized at 200°C	200	T	0.63	1
Lead	oxidized, gray	20	T	0.28	1
Lead	oxidized, gray	22	T	0.28	4
Lead	shiny	250	T	0.08	1
Lead	unoxidized, polished	100	T	0.05	4
Lead red		100	T	0.93	4
Lead red, powder		100	T	0.93	1
Leather	tanned		T	0.75–0.80	1
Lime			T	0.3–0.4	1
Magnesium		22	T	0.07	4
Magnesium		260	T	0.13	4
Magnesium		538	T	0.18	4
Magnesium	polished	20	T	0.07	2
Magnesium powder			T	0.86	1
Molybdenum		1500–2200	T	0.19–0.26	1
Molybdenum		600–1000	T	0.08–0.13	1
Molybdenum	filament	700–2500	T	0.1–0.3	1
Mortar		17	SW	0.87	5
Mortar	dry	36	SW	0.94	7
Nextel Velvet 811-21 Black	Flat black	–60–150	LW	> 0.97	10 and 11

Table 21.1 T: Total spectrum; SW: 2–5 µm; LW: 8–14 µm, LLW: 6.5–20 µm; 1: Material; 2: Specification; 3: Temperature in °C; 4: Spectrum; 5: Emissivity: 6:Reference (continued)

1	2	3	4	5	6
Nichrome	rolled	700	T	0.25	1
Nichrome	sandblasted	700	T	0.70	1
Nichrome	wire, clean	50	T	0.65	1
Nichrome	wire, clean	500–1000	T	0.71–0.79	1
Nichrome	wire, oxidized	50–500	T	0.95–0.98	1
Nickel	bright matte	122	T	0.041	4
Nickel	commercially pure, polished	100	T	0.045	1
Nickel	commercially pure, polished	200–400	T	0.07–0.09	1
Nickel	electrolytic	22	T	0.04	4
Nickel	electrolytic	260	T	0.07	4
Nickel	electrolytic	38	T	0.06	4
Nickel	electrolytic	538	T	0.10	4
Nickel	electroplated on iron, polished	22	T	0.045	4
Nickel	electroplated on iron, unpolished	20	T	0.11–0.40	1
Nickel	electroplated on iron, unpolished	22	T	0.11	4
Nickel	electroplated, polished	20	T	0.05	2
Nickel	oxidized	1227	T	0.85	4
Nickel	oxidized	200	T	0.37	2
Nickel	oxidized	227	T	0.37	4
Nickel	oxidized at 600°C	200–600	T	0.37–0.48	1
Nickel	polished	122	T	0.045	4
Nickel	wire	200–1000	T	0.1–0.2	1
Nickel oxide		1000–1250	T	0.75–0.86	1
Nickel oxide		500–650	T	0.52–0.59	1
Oil, lubricating	0.025 mm film	20	T	0.27	2
Oil, lubricating	0.050 mm film	20	T	0.46	2
Oil, lubricating	0.125 mm film	20	T	0.72	2
Oil, lubricating	film on Ni base: Ni base only	20	T	0.05	2
Oil, lubricating	thick coating	20	T	0.82	2
Paint	8 different colors and qualities	70	SW	0.88–0.96	9
Paint	8 different colors and qualities	70	LW	0.92–0.94	9
Paint	Aluminum, various ages	50–100	T	0.27–0.67	1
Paint	cadmium yellow		T	0.28–0.33	1

Table 21.1 T: Total spectrum; SW: 2–5 µm; LW: 8–14 µm, LLW: 6.5–20 µm; 1: Material; 2: Specification; 3: Temperature in °C; 4: Spectrum; 5: Emissivity: 6:Reference (continued)

1	2	3	4	5	6
Paint	chrome green		T	0.65–0.70	1
Paint	cobalt blue		T	0.7–0.8	1
Paint	oil	17	SW	0.87	5
Paint	oil based, average of 16 colors	100	T	0.94	2
Paint	oil, black flat	20	SW	0.94	6
Paint	oil, black gloss	20	SW	0.92	6
Paint	oil, gray flat	20	SW	0.97	6
Paint	oil, gray gloss	20	SW	0.96	6
Paint	oil, various colors	100	T	0.92–0.96	1
Paint	plastic, black	20	SW	0.95	6
Paint	plastic, white	20	SW	0.84	6
Paper	4 different colors	70	SW	0.68–0.74	9
Paper	4 different colors	70	LW	0.92–0.94	9
Paper	black		T	0.90	1
Paper	black, dull		T	0.94	1
Paper	black, dull	70	SW	0.86	9
Paper	black, dull	70	LW	0.89	9
Paper	blue, dark		T	0.84	1
Paper	coated with black lacquer		T	0.93	1
Paper	green		T	0.85	1
Paper	red		T	0.76	1
Paper	white	20	T	0.7–0.9	1
Paper	white bond	20	T	0.93	2
Paper	white, 3 different glosses	70	SW	0.76–0.78	9
Paper	white, 3 different glosses	70	LW	0.88–0.90	9
Paper	yellow		T	0.72	1
Plaster		17	SW	0.86	5
Plaster	plasterboard, untreated	20	SW	0.90	6
Plaster	rough coat	20	T	0.91	2
Plastic	glass fibre laminate (printed circ. board)	70	SW	0.94	9
Plastic	glass fibre laminate (printed circ. board)	70	LW	0.91	9
Plastic	polyurethane isolation board	70	LW	0.55	9

Table 21.1 T: Total spectrum; SW: 2–5 µm; LW: 8–14 µm, LLW: 6.5–20 µm; 1: Material; 2: Specification; 3: Temperature in °C; 4: Spectrum; 5: Emissivity: 6:Reference (continued)

1	2	3	4	5	6
Plastic	polyurethane isolation board	70	SW	0.29	9
Plastic	PVC, plastic floor, dull, structured	70	SW	0.94	9
Plastic	PVC, plastic floor, dull, structured	70	LW	0.93	9
Platinum		100	T	0.05	4
Platinum		1000–1500	T	0.14–0.18	1
Platinum		1094	T	0.18	4
Platinum		17	T	0.016	4
Platinum		22	T	0.03	4
Platinum		260	T	0.06	4
Platinum		538	T	0.10	4
Platinum	pure, polished	200–600	T	0.05–0.10	1
Platinum	ribbon	900–1100	T	0.12–0.17	1
Platinum	wire	1400	T	0.18	1
Platinum	wire	500–1000	T	0.10–0.16	1
Platinum	wire	50–200	T	0.06–0.07	1
Porcelain	glazed	20	T	0.92	1
Porcelain	white, shiny		T	0.70–0.75	1
Rubber	hard	20	T	0.95	1
Rubber	soft, gray, rough	20	T	0.95	1
Sand			T	0.60	1
Sand		20	T	0.90	2
Sandstone	polished	19	LLW	0.909	8
Sandstone	rough	19	LLW	0.935	8
Silver	polished	100	T	0.03	2
Silver	pure, polished	200–600	T	0.02–0.03	1
Skin	human	32	T	0.98	2
Slag	boiler	0–100	T	0.97–0.93	1
Slag	boiler	1400–1800	T	0.69–0.67	1
Slag	boiler	200–500	T	0.89–0.78	1
Slag	boiler	600–1200	T	0.76–0.70	1
Snow: See Water					
Soil	dry	20	T	0.92	2
Soil	saturated with water	20	T	0.95	2
Stainless steel	alloy, 8% Ni, 18% Cr	500	T	0.35	1
Stainless steel	rolled	700	T	0.45	1
Stainless steel	sandblasted	700	T	0.70	1
Stainless steel	sheet, polished	70	SW	0.18	9

Table 21.1 T: Total spectrum; SW: 2–5 µm; LW: 8–14 µm, LLW: 6.5–20 µm; 1: Material; 2: Specification; 3: Temperature in °C; 4: Spectrum; 5: Emissivity: 6:Reference (continued)

1	2	3	4	5	6
Stainless steel	sheet, polished	70	LW	0.14	9
Stainless steel	sheet, untreated, somewhat scratched	70	SW	0.30	9
Stainless steel	sheet, untreated, somewhat scratched	70	LW	0.28	9
Stainless steel	type 18-8, buffed	20	T	0.16	2
Stainless steel	type 18-8, oxi-dized at 800°C	60	T	0.85	2
Stucco	rough, lime	10–90	T	0.91	1
Styrofoam	insulation	37	SW	0.60	7
Tar			T	0.79–0.84	1
Tar	paper	20	T	0.91–0.93	1
Tile	glazed	17	SW	0.94	5
Tin	burnished	20–50	T	0.04–0.06	1
Tin	tin–plated sheet iron	100	T	0.07	2
Titanium	oxidized at 540°C	1000	T	0.60	1
Titanium	oxidized at 540°C	200	T	0.40	1
Titanium	oxidized at 540°C	500	T	0.50	1
Titanium	polished	1000	T	0.36	1
Titanium	polished	200	T	0.15	1
Titanium	polished	500	T	0.20	1
Tungsten		1500–2200	T	0.24–0.31	1
Tungsten		200	T	0.05	1
Tungsten		600–1000	T	0.1–0.16	1
Tungsten	filament	3300	T	0.39	1
Varnish	flat	20	SW	0.93	6
Varnish	on oak parquet floor	70	SW	0.90	9
Varnish	on oak parquet floor	70	LW	0.90–0.93	9
Wallpaper	slight pattern, light gray	20	SW	0.85	6
Wallpaper	slight pattern, red	20	SW	0.90	6
Water	distilled	20	T	0.96	2
Water	frost crystals	–10	T	0.98	2
Water	ice, covered with heavy frost	0	T	0.98	1
Water	ice, smooth	0	T	0.97	1
Water	ice, smooth	–10	T	0.96	2
Water	layer >0.1 mm thick	0–100	T	0.95–0.98	1

Table 21.1 T: Total spectrum; SW: 2–5 µm; LW: 8–14 µm, LLW: 6.5–20 µm; 1: Material; 2: Specification; 3: Temperature in °C; 4: Spectrum; 5: Emissivity: 6:Reference (continued)

1	2	3	4	5	6
Water	snow		T	0.8	1
Water	snow	–10	T	0.85	2
Wood		17	SW	0.98	5
Wood		19	LLW	0.962	8
Wood	ground		T	0.5–0.7	1
Wood	pine, 4 different samples	70	SW	0.67–0.75	9
Wood	pine, 4 different samples	70	LW	0.81–0.89	9
Wood	planed	20	T	0.8–0.9	1
Wood	planed oak	20	T	0.90	2
Wood	planed oak	70	SW	0.77	9
Wood	planed oak	70	LW	0.88	9
Wood	plywood, smooth, dry	36	SW	0.82	7
Wood	plywood, untreated	20	SW	0.83	6
Wood	white, damp	20	T	0.7–0.8	1
Zinc	oxidized at 400°C	400	T	0.11	1
Zinc	oxidized surface	1000–1200	T	0.50–0.60	1
Zinc	polished	200–300	T	0.04–0.05	1
Zinc	sheet	50	T	0.20	1

A note on the technical production of this publication

This publication was produced using XML — the eXtensible Markup Language. For more information about XML, please visit http://www.w3.org/XML/

A note on the typeface used in this publication

This publication was typeset using Linotype Helvetica™ World. Helvetica™ was designed by Max Miedinger (1910–1980)

LOEF (List Of Effective Files)

T501027.xml; en-US; AL; 42258; 2017-04-27
T505552.xml; en-US; 9599; 2013-11-05
T505469.xml; en-US; 39689; 2017-01-25
T505013.xml; en-US; 39689; 2017-01-25
T505545.xml; en-US; 39841; 2017-01-30
T505547.xml; en-US; 39841; 2017-01-30
T505550.xml; en-US; 40804; 2017-03-02
T505786.xml; en-US; 40282; 2017-02-14
T505470.xml; en-US; 39513; 2017-01-18
T505012.xml; en-US; 41563; 2017-03-23
T505007.xml; en-US; 39512; 2017-01-18
T506125.xml; en-US; 40753; 2017-03-02
T505000.xml; en-US; 39687; 2017-01-25
T506051.xml; en-US; 40460; 2017-02-20
T505005.xml; en-US; 41563; 2017-03-23
T505001.xml; en-US; 41563; 2017-03-23
T505006.xml; en-US; 41563; 2017-03-23
T505002.xml; en-US; 39512; 2017-01-18

Website
http://www.flir.com

Customer support
http://support.flir.com

Copyright
© 2017, FLIR Systems, Inc. All rights reserved worldwide.

Disclaimer
Specifications subject to change without further notice. Models and accessories subject to regional market considerations. License procedures may apply. Products described herein may be subject to US Export Regulations. Please refer to exportquestions@flir.com with any questions.

Publ. No.:	T559828
Release:	AL
Commit:	42258
Head:	42258
Language:	en-US
Modified:	2017-04-27
Formatted:	2017-05-02
